# Species conservation profiles of a random sample of world spiders IV: Scytodidae to Zoropsidae

**DOI:** 10.3897/BDJ.6.e30842

**Published:** 2018-12-14

**Authors:** Sini Seppälä, Sérgio Henriques, Michael L Draney, Stefan Foord, Alastair T Gibbons, Luz A Gomez, Sarah Kariko, Jagoba Malumbres-Olarte, Marc Milne, Cor J Vink, Pedro Cardoso

**Affiliations:** 1 LIBRe - Laboratory for Integrative Biodiversity Research, Finnish Museum of Natural History, University of Helsinki, Helsinki, Finland LIBRe - Laboratory for Integrative Biodiversity Research, Finnish Museum of Natural History, University of Helsinki Helsinki Finland; 2 IUCN SSC Spider & Scorpion Specialist Group, Helsinki, Finland IUCN SSC Spider & Scorpion Specialist Group Helsinki Finland; 3 University College London, London, United Kingdom University College London London United Kingdom; 4 University of Wisconsin-Green Bay, Green Bay, United States of America University of Wisconsin-Green Bay Green Bay United States of America; 5 University of Venda, Thohyandou, South Africa University of Venda Thohyandou South Africa; 6 University of Nottingham, Nottingham, United Kingdom University of Nottingham Nottingham United Kingdom; 7 Universidad Nacional de Colombia, Bogotá, Colombia Universidad Nacional de Colombia Bogotá Colombia; 8 Museum of Comparative Zoology, Harvard University, Cambridge, United States of America Museum of Comparative Zoology, Harvard University Cambridge United States of America; 9 cE3c - Centre for Ecology, Evolution and Environmental Changes, University of the Azores, Angra do Heroísmo, Portugal cE3c - Centre for Ecology, Evolution and Environmental Changes, University of the Azores Angra do Heroísmo Portugal; 10 University of Barcelona, Barcelona, Spain University of Barcelona Barcelona Spain; 11 University of Copenhagen, Copenhagen, Denmark University of Copenhagen Copenhagen Denmark; 12 University of Indianapolis, Indianapolis, United States of America University of Indianapolis Indianapolis United States of America; 13 Canterbury Museum, Christchurch, New Zealand Canterbury Museum Christchurch New Zealand

**Keywords:** Araneae, Arthropoda, conservation, endangered species, extinction risk, geographic range, IUCN

## Abstract

**Background:**

The IUCN Red List of Threatened Species is the most widely used information source on the extinction risk of species. One of the uses of the Red List is to evaluate and monitor the state of biodiversity and a possible approach for this purpose is the Red List Index (RLI). For many taxa, mainly hyperdiverse groups, it is not possible within available resources to assess all known species. In such cases, a random sample of species might be selected for assessment and the results derived from it extrapolated for the entire group - the Sampled Red List Index (SRLI). The current contribution is the final in four papers that will constitute the baseline of a future spider SRLI encompassing 200 species distributed across the world.

**New information:**

A sample of 200 species of spiders were randomly selected from the World Spider Catalogue, an updated global database containing all recognised species names for the group. The selected species were classified taxonomically at the family level and the familes were ordered alphabetically. In this publication, we present the conservation profiles of 50 species belonging to the families alphabetically arranged between Scytodidae and Zoropsidae, which encompassed Scytodidae, Selenopidae, Sicariidae, Sparassidae, Tetrablemmidae, Tetragnathidae, Theraphosidae, Theridiidae, Theridiosomatidae, Thomisidae, Trochanteriidae, Zodariidae and Zoropsidae.

## Introduction

The IUCN Red List of Threatened Species is the most widely used information source on the extinction risk of species (Lamoreux et al. 2003, Rodrigues et al. 2006, Mace et al. 2008 but see Cardoso et al. 2011b, Cardoso et al. 2012). It is based on a number of objective criteria, which are relatively easy to apply when adequate information is available (IUCN 2001). The Red List has been used to raise awareness about threatened species, guide conservation efforts and funding, set priorities for protection, measure site irreplaceability and vulnerability and influence environmental policies and legislation (Gardenfors et al. 2001, Rodrigues et al. 2006, Mace et al. 2008, Martin-Lopez et al. 2009).

One of the uses of the Red List is to evaluate and monitor the state of biodiversity and a possible approach for this purpose is the Red List Index (RLI). The RLI helps to develop a better understanding of which taxa, regions or ecosystems are declining or improving their conservation status. It provides policy-makers, stakeholders, conservation practitioners and the general public with sound knowledge of biodiversity status and change and tools to make informed decisions. The RLI uses weight scores based on the Red List status of each of the assessed species. These scores range from 0 (Least Concern) to 5 (Extinct/Extinct in the Wild). Summing these scores across all species, relating them to the worst-case scenario - all species extinct and comparing two or more points in time, gives us an indication of how biodiversity is doing. At a global level, the RLI has been calculated for birds (Butchart et al. 2004, Hoffmann et al. 2010), mammals (Hoffmann et al. 2011), amphibians (Hoffmann et al. 2010), corals (Butchart et al. 2010) and cycads (United Nations 2015).

For many taxa, mainly hyperdiverse groups, it is not possible within available resources to assess all known species. In such cases, a random sample of species might be selected for assessment and the results derived from it extrapolated to the entire group - the Sampled Red List Index (SRLI, Baillie et al. 2008). The SRLI is now being developed for plants (Brummitt et al. 2015) and efforts towards a SRLI of butterflies (Lewis and Senior 2010) and Odonata are also in progress (Clausnitzer et al. 2009).

Spiders currently comprise over 47000 species described at the global level (World Spider Catalog 2018). Of these, only 200 species (0.4%) have been assessed (www.redlist.org), of which the vast majority are from the Seychelles Islands or belong to the golden-orb weavers, Nephilidae. To these, a large number will be added in the near future, such as 55 species endemic to the Madeira and Selvagens archipelagos and 25 endemic to the Azores, all in Portugal (Cardoso et al. 2017, Borges et al. submitted). The vast majority of spiders assessed to date are therefore either regionally or taxonomically clustered and do not represent the group as a whole. The current contribution is the final in four papers (Seppälä et al. 2018a, Seppälä et al. 2018b, Seppälä et al. 2018c) that constitute the baseline of a future spider SRLI encompassing 200 species distributed across the world. All the assessments will, in the future, be included in the IUCN Red List of Threatened Species (www.redlist.org).

## Methods

A sample of 200 species of spiders were randomly selected from the World Spider Catalog (2018), an updated global database containing all recognised species names for the group. The 200 selected species were divided taxonomically to the family level and those families were ordered alphabetically. In this publication, we present the conservation profiles of 58 species belonging to the families alphabetically arranged between Scytodidae and Zoropsidae, which encompassed Scytodidae, Selenopidae, Sicariidae, Sparassidae, Tetrablemmidae, Tetragnathidae, Theraphosidae, Theridiidae, Theridiosomatidae, Thomisidae, Trochanteriidae, Zodariidae and Zoropsidae.

Species data were collected from all taxonomic bibliography available at the World Spider Catalog (2018), complemented by data in other publications found through Google Scholar and georeferenced points made available through the Global Biodiversity Information Facility (www.gbif.org) and also other sources (https://www.biodiversitylibrary.org; https://login.webofknowledge.com; http://srs.britishspiders.org.uk; http://symbiota4.acis.ufl.edu/scan/portal; https://lepus.unine.ch; http://www.tuite.nl/iwg/Araneae/SpiBenelux/?species; https://atlas.arages.de; https://arachnology.cz/rad/araneae-1.html; http://www.ennor.org/iberia). Whenever possible, with each species record, we also collected additional information, namely habitat type and spatial error of coordinates.

For all analyses, we used the R package 'red' - IUCN red-listing tools (Cardoso 2017). This package performs a number of spatial analyses based on either observed occurrences or estimated ranges. Functions include calculating Extent of Occurrence (EOO), Area of Occupancy (AOO), mapping species ranges, species distribution modelling using climate and land cover, calculating the Red List Index for groups of species, amongst others. In this work, the EOO and AOO were calculated in one of two ways:

- For extremely range-restricted species for which we assumed to know the full range, these values were classified as observed, the minimum convex polygon encompassing all observations used to calculate the EOO and the 2 km x 2 km cells known to be occupied were used to calculate the AOO. When the EOO was smaller than the AOO, it was made equal as per the IUCN guidelines (IUCN Standards and Petitions Subcommittee 2017).

- For widespread species or those for which we did not have confidence to know the full range, we performed species distribution modelling (SDM). This was done based on both climatic (Fick and Hijmans 2017) and landcover (Tuanmu and Jetz 2014) datasets, at an approximately 1 x 1 km resolution. Before modelling, the world layers were cropped to the region of interest to each species and reduced to four layers through a PCA to avoid overfitting. In addition, latitude and longitude were used as two extra layers to prevent the models from predicting presences far beyond the known region following the precautionary principle. We then used the Maxent method (Phillips et al. 2006) implemented in the R package 'red'. Isolated patches outside the original distribution polygon were excluded from maps to avoid overestimation of EOO and AOO values. All final maps and values were checked and validated by the authors. KMLs derived from these maps were also produced using the red package. The cells (2x2 km), predicted to be occupied, were used to calculate the AOO. When the EOO was smaller than the AOO, it was made equal as per the IUCN guidelines (IUCN Standards and Petitions Subcommittee 2017).

To infer possible changes in range and/or abundance and for forest species only, we also consulted the Global Forest Watch portal (World Resources Institute 2014), looking for changes in forest cover during the last 10 years that could have affected the species.

Species sizes are total body size in mm and include the ranges for both males and females when known.

## Species Conservation Profiles

### Dictis denticulata

#### Species information

Scientific name: Dictis denticulata

Species authority: Dankittipakul & Singtripop, 2010

Kingdom: Animalia

Phylum: Arthropoda

Class: Arachnida

Order: Araneae

Family: Scytodidae

Region for assessment: Global

#### Geographic range

Biogeographic realm: Indomalayan

Countries: ThailandLao People's Democratic RepublicMyanmar

Map of records (Google Earth): Suppl. material 1

Basis of EOO and AOO: Species Distribution Model

Basis (narrative): Given the high number of recent records (Dankittipakul and Singtripop 2010), it was possible to perform species distribution modelling (see methods for details).

Min Elevation/Depth (m): 150

Max Elevation/Depth (m): 790

Range description: This species has been recorded from multiple localities in northern Thailand. The species distribution model predicts this species could also be present in northwestern Laos and eastern Myanmar.

#### New occurrences

#### Extent of occurrence

EOO (km2): 257202

Trend: Stable

Justification for trend: As it is a relatively widespread species with no known threats, we infer the trend to be stable.

Causes ceased?: Yes

Causes understood?: Yes

Causes reversible?: Yes

Extreme fluctuations?: No

#### Area of occupancy

Trend: Stable

Justification for trend: As it is a relatively widespread species with no known threats, we infer the trend to be stable.

Causes ceased?: Yes

Causes understood?: Yes

Causes reversible?: Yes

Extreme fluctuations?: No

AOO (km2): 104152

#### Locations

Number of locations: Not applicable

Justification for number of locations: No known threats to the species.

Trend: Stable

Extreme fluctuations?: No

#### Population

Number of individuals: Unknown

Trend: Stable

Justification for trend: As it is a relatively widespread species, we infer the trend to be stable.

Causes ceased?: Yes

Causes understood?: Yes

Causes reversible?: Yes

Extreme fluctuations?: No

Population Information (Narrative): No population size estimates exist.

#### Subpopulations

Number of subpopulations: Unknown

Trend: Stable

Justification for trend: As it is a relatively widespread species with no known threats, we infer the trend to be stable.

Extreme fluctuations?: No

Severe fragmentation?: No

#### Habitat

System: Terrestrial

Habitat specialist: No

Habitat (narrative): This species has been observed in deciduous dipterocarp forests. Observations have also been made around human infrastructures and from siamese tulip fields (Dankittipakul and Singtripop 2010).

Trend in extent, area or quality?: Stable

Justification for trend: This species seems not to be restricted to any particular habitat type and to be tolerant to human disturbance.

##### Habitat

Habitat importance: Major Importance

Habitats: 1.6. Forest - Subtropical/Tropical Moist Lowland

##### Habitat

Habitat importance: Suitable

Habitats: 14.3. Artificial/Terrestrial - Plantations14.5. Artificial/Terrestrial - Urban Areas

#### Habitat

Habitat importance: Major Importance

Habitats: 1.6. Forest - Subtropical/Tropical Moist Lowland

#### Habitat

Habitat importance: Suitable

Habitats: 14.3. Artificial/Terrestrial - Plantations14.5. Artificial/Terrestrial - Urban Areas

#### Ecology

Size: 4 mm

Generation length (yr): 1

Dependency of single sp?: Unknown

Ecology and traits (narrative): Both females and males of this species were collected by Malaise traps. This suggest they are free-living ground-dwellers hunting actively (Dankittipakul and Singtripop 2010). Scytodids, spitting spiders, in general are cursorial and nocturnal hunters that have specialised prey catching techniques. These spiders are also the only ones that are known to have prosomal glands that secrete not only venom but also silk. Scytodids are able to squirt a mixture of venom and gluey silk towards its prey which then gets stuck in the substrate, the venom causing a paralysis. The female lays eggs in a silken retreat and the eggs are carried in the chelicerae and pulled together with a couple of silk threads (Dippenaar-Schoeman and Jocqué 1997).

#### Threats

Justification for threats: No known threats to the species.

##### Threats

Threat type: Past

Threats: 12. Other options - Other threat

#### Threats

Threat type: Past

Threats: 12. Other options - Other threat

#### Conservation

Justification for conservation actions: There are several protected areas inside the range of this species (United Nations Environment World Conservation Monitoring Centre 2017).

##### Conservation actions

Conservation action type: In Place

Conservation actions: 1.1. Land/water protection - Site/area protection1.2. Land/water protection - Resource & habitat protection

#### Conservation actions

Conservation action type: In Place

Conservation actions: 1.1. Land/water protection - Site/area protection1.2. Land/water protection - Resource & habitat protection

#### Other

##### Use and trade

Use type: International

##### Ecosystem services

Ecosystem service type: Very important

##### Research needed

Research needed: 3.1. Monitoring - Population trends3.4. Monitoring - Habitat trends

Justification for research needed: Monitoring is needed to confirm current habitat and population trends.

#### Use and trade

Use type: International

#### Ecosystem services

Ecosystem service type: Very important

#### Research needed

Research needed: 3.1. Monitoring - Population trends3.4. Monitoring - Habitat trends

Justification for research needed: Monitoring is needed to confirm current habitat and population trends.

#### Viability analysis

### Scytodes cogu

#### Species information

Scientific name: Scytodes cogu

Species authority: Brescovit & Rheims, 2001

Kingdom: Animalia

Phylum: Arthropoda

Class: Arachnida

Order: Araneae

Family: Scytodidae

Region for assessment: Global

#### Geographic range

Biogeographic realm: Neotropical

Countries: PanamaCosta Rica

Map of records (Google Earth): Suppl. material 2

Basis of EOO and AOO: Species Distribution Model

Basis (narrative): Given the reasonable number of records (Brescovit and Rheims 2001, Valerio 1981), it was possible to perform species distribution modelling (see methods for details).

Min Elevation/Depth (m): 0

Max Elevation/Depth (m): 3730

Range description: This species has been recorded from four localities in Costa Rica. In 2001, it was reported from San Jose (Brescovit and Rheims 2001) and in 1980s from La Gloria, Guanacaste Province and Tilaran (Valerio 1981). The species distribution model predicts this species could also be present in Panama.

#### New occurrences

#### Extent of occurrence

EOO (km2): 52355

Trend: Unknown

Causes ceased?: Unknown

Causes understood?: Unknown

Causes reversible?: Unknown

Extreme fluctuations?: Unknown

#### Area of occupancy

Trend: Unknown

Causes ceased?: Unknown

Causes understood?: Unknown

Causes reversible?: Unknown

Extreme fluctuations?: Unknown

AOO (km2): 33872

#### Locations

Number of locations: Unknown

Trend: Unknown

Extreme fluctuations?: Unknown

#### Population

Number of individuals: Unknown

Trend: Unknown

Causes ceased?: Unknown

Causes understood?: Unknown

Causes reversible?: Unknown

Extreme fluctuations?: Unknown

Population Information (Narrative): No population size estimates exist.

#### Subpopulations

Number of subpopulations: Unknown

Trend: Unknown

Extreme fluctuations?: Unknown

Severe fragmentation?: Unknown

#### Habitat

System: Terrestrial

Habitat specialist: Unknown

Habitat (narrative): This species is known to live in lowland tropical forest (Valerio 1981) but the SDMs indicate it might be able to occupy higher altitudes.

Trend in extent, area or quality?: Unknown

##### Habitat

Habitat importance: Major Importance

Habitats: 1.6. Forest - Subtropical/Tropical Moist Lowland

#### Habitat

Habitat importance: Major Importance

Habitats: 1.6. Forest - Subtropical/Tropical Moist Lowland

#### Ecology

Size: 3.38 - 4.63 mm

Generation length (yr): 1

Dependency of single sp?: Unknown

Ecology and traits (narrative): Scytodids, spitting spiders, in general are cursorial and nocturnal hunters that have specialised prey catching techniques. These spiders are also the only ones that are known to have prosomal glands that secrete not only venom but also silk. Scytodids are able to squirt a mixture of venom and gluey silk towards its prey which then gets stuck in the substrate, the venom causing paralysis. Females lay eggs in a silken retreat and the eggs are carried in the chelicerae and pulled together with a couple of silk threads (Dippenaar-Schoeman and Jocqué 1997). *Scytodes* females may tolerate the presence of their offspring for some time after hatching (Nentwig 1985).

#### Threats

Justification for threats: There has been a forest loss of 199,007 ha in Costa Rica between the years 2001 and 2016 (Global Forest Watch 2014). In southern Costa Rica in particular, almost three quarters of its forested habitat has been lost. Regeneration has offset the deforestation to some point, however, the continued fragmentation of forests and increasing edge habitats can finally lead to a decline in habitat quality and therefore in population size (Zahawi et al. 2015). However, we do not know for certain whether this species is dependent on these forests and what are the effects to its survival in case the decline continues.

##### Threats

Threat type: Ongoing

Threats: 2.1. Agriculture & aquaculture - Annual & perennial non-timber crops2.2. Agriculture & aquaculture - Wood & pulp plantations2.3. Agriculture & aquaculture - Livestock farming & ranching

#### Threats

Threat type: Ongoing

Threats: 2.1. Agriculture & aquaculture - Annual & perennial non-timber crops2.2. Agriculture & aquaculture - Wood & pulp plantations2.3. Agriculture & aquaculture - Livestock farming & ranching

#### Conservation

Justification for conservation actions: There are several protected areas within the range of this species, for example La Amistad National Park (United Nations Environment World Conservation Monitoring Centre 2017).

##### Conservation actions

Conservation action type: In Place

Conservation actions: 1.1. Land/water protection - Site/area protection1.2. Land/water protection - Resource & habitat protection

#### Conservation actions

Conservation action type: In Place

Conservation actions: 1.1. Land/water protection - Site/area protection1.2. Land/water protection - Resource & habitat protection

#### Other

##### Use and trade

Use type: International

Use and trade: 18. Unknown

##### Ecosystem services

Ecosystem service type: Very important

##### Research needed

Research needed: 1.5. Research - Threats3.1. Monitoring - Population trends3.4. Monitoring - Habitat trends

Justification for research needed: Monitoring is needed to know current population and habitat trends and to explore whether the continuing forest loss is a plausible threat to this species.

#### Use and trade

Use type: International

Use and trade: 18. Unknown

#### Ecosystem services

Ecosystem service type: Very important

#### Research needed

Research needed: 1.5. Research - Threats3.1. Monitoring - Population trends3.4. Monitoring - Habitat trends

Justification for research needed: Monitoring is needed to know current population and habitat trends and to explore whether the continuing forest loss is a plausible threat to this species.

#### Viability analysis

### Selenops candidus

#### Species information

Scientific name: Selenops candidus

Species authority: Muma, 1953

Kingdom: Animalia

Phylum: Arthropoda

Class: Arachnida

Order: Araneae

Family: Selenopidae

Region for assessment: Global

#### Geographic range

Biogeographic realm: Neotropical

Countries: Jamaica

Map of records (Google Earth): Suppl. material 3

Basis of EOO and AOO: Species Distribution Model

Basis (narrative): Given the relatively high number of records (Muma 1953, Crews 2011), it was possible to perform species distribution modelling (see methods for details).

Min Elevation/Depth (m): 0

Max Elevation/Depth (m): 500

Range description: This species has been recorded from Jamaica only, first prior to 1935 (Muma 1953) and then several records in 2006 (Crews 2011). This species is endemic to Jamaica, although it has been transported on bananas to New York (Crews 2011).

#### New occurrences

#### Extent of occurrence

EOO (km2): 9654

Trend: Stable

Justification for trend: As it is a relatively widespread species with no known threats, able to live in different habitat types, we infer the trend to be stable.

Causes ceased?: Yes

Causes understood?: Yes

Causes reversible?: Yes

Extreme fluctuations?: No

#### Area of occupancy

Trend: Stable

Justification for trend: As it is a relatively widespread species with no known threats, able to live in different habitat types, we infer the trend to be stable.

Causes ceased?: Yes

Causes understood?: Yes

Causes reversible?: Yes

Extreme fluctuations?: No

AOO (km2): 4200

#### Locations

Number of locations: Not applicable

Justification for number of locations: No known threats to the species.

Trend: Stable

Extreme fluctuations?: No

#### Population

Number of individuals: Unknown

Trend: Stable

Justification for trend: As it is a relatively widespread species with no known threats, able to live in different habitat types, we infer the trend to be stable.

Causes ceased?: Yes

Causes understood?: Yes

Causes reversible?: Yes

Extreme fluctuations?: No

Population Information (Narrative): No population size estimates exist.

#### Subpopulations

Number of subpopulations: Unknown

Trend: Stable

Justification for trend: As it is a relatively widespread species with no known threats, able to live in different habitat types, we infer the trend to be stable.

Extreme fluctuations?: No

Severe fragmentation?: No

#### Habitat

System: Terrestrial

Habitat specialist: No

Habitat (narrative): This species has been reported from dry coastal limestone forests, inland dry forests and banana plantations from sea level to 500 m altitude (Crews 2011).

Trend in extent, area or quality?: Stable

Justification for trend: This is a ground-dwelling species hiding in crevices, found in different forest types and plantations and assumed not to be affected by forest loss.

##### Habitat

Habitat importance: Major Importance

Habitats: 1.5. Forest - Subtropical/Tropical Dry

##### Habitat

Habitat importance: Suitable

Habitats: 14.3. Artificial/Terrestrial - Plantations

#### Habitat

Habitat importance: Major Importance

Habitats: 1.5. Forest - Subtropical/Tropical Dry

#### Habitat

Habitat importance: Suitable

Habitats: 14.3. Artificial/Terrestrial - Plantations

#### Ecology

Size: 18.85 - 19.70 mm

Generation length (yr): 1

Dependency of single sp?: No

Ecology and traits (narrative): Selenopids, commonly known as flatties or wall spiders, are wandering spiders living free and usually found on walls or under rocks. Due to their flat habitus, they can hide inside narrow crevices. When disturbed these spiders flee moving sideways to hide. Egg sacs of this species are single and flat, paper-like and they are attached under bark where the female guards them (Dippenaar-Schoeman and Jocqué 1997, Crews 2011). This species seems to do quite well in transformed landscapes and has been collected on *Eucalyptus* and banana plantations (Crews 2011).

#### Threats

Justification for threats: No known threats to the species.

##### Threats

Threat type: Past

Threats: 12. Other options - Other threat

#### Threats

Threat type: Past

Threats: 12. Other options - Other threat

#### Conservation

Justification for conservation actions: There is at least one protected area, Portland Bight, within the range of this species (United Nations Environment World Conservation Monitoring Centre 2017).

##### Conservation actions

Conservation action type: In Place

Conservation actions: 1.1. Land/water protection - Site/area protection1.2. Land/water protection - Resource & habitat protection

#### Conservation actions

Conservation action type: In Place

Conservation actions: 1.1. Land/water protection - Site/area protection1.2. Land/water protection - Resource & habitat protection

#### Other

##### Use and trade

Use type: International

##### Ecosystem services

Ecosystem service type: Very important

##### Research needed

Research needed: 3.1. Monitoring - Population trends3.4. Monitoring - Habitat trends

Justification for research needed: Monitoring is needed to confirm habitat and population trends.

#### Use and trade

Use type: International

#### Ecosystem services

Ecosystem service type: Very important

#### Research needed

Research needed: 3.1. Monitoring - Population trends3.4. Monitoring - Habitat trends

Justification for research needed: Monitoring is needed to confirm habitat and population trends.

#### Viability analysis

### Selenops shevaroyensis

#### Species information

Scientific name: Selenops shevaroyensis

Species authority: Gravely, 1931

Kingdom: Animalia

Phylum: Arthropoda

Class: Arachnida

Order: Araneae

Family: Selenopidae

Taxonomic notes: According to Gravely (1931), *S. shevaroyensis* resembles *S. radiatus.* Taxonomic clarification would be essential.

Region for assessment: Global

#### Geographic range

Biogeographic realm: Indomalayan

Countries: India

Map of records (Google Earth): Suppl. material 4

Basis of EOO and AOO: Unknown

Basis (narrative): Unknown EOO or AOO.

Min Elevation/Depth (m): 1350

Max Elevation/Depth (m): 1350

Range description: A single specimen is known from the type locality in Yercaud, India, recorded prior to 1931 (Gravely 1931).

#### New occurrences

#### Extent of occurrence

EOO (km2): Unknown

Trend: Unknown

Causes ceased?: Unknown

Causes understood?: Unknown

Causes reversible?: Unknown

Extreme fluctuations?: Unknown

#### Area of occupancy

Trend: Unknown

Causes ceased?: Unknown

Causes understood?: Unknown

Causes reversible?: Unknown

Extreme fluctuations?: Unknown

AOO (km2): Unknown

#### Locations

Number of locations: Unknown

Trend: Unknown

Extreme fluctuations?: Unknown

#### Population

Number of individuals: Unknown

Trend: Unknown

Causes ceased?: Unknown

Causes understood?: Unknown

Causes reversible?: Unknown

Extreme fluctuations?: Unknown

Population Information (Narrative): Population size and trend are unknown.

#### Subpopulations

Number of subpopulations: Unknown

Trend: Unknown

Extreme fluctuations?: Unknown

Severe fragmentation?: Unknown

#### Habitat

System: Terrestrial

Habitat specialist: Unknown

Habitat (narrative): Yercaud belongs to the ecoregion of tropical and subtropical dry broadleaf forests (Olson et al. 2001). Otherwise, with only one over 80 years old record, the preferred habitat remains unknown.

Trend in extent, area or quality?: Unknown

##### Habitat

Habitat importance: Major Importance

Habitats: 18. Unknown

#### Habitat

Habitat importance: Major Importance

Habitats: 18. Unknown

#### Ecology

Size: Unknown

Generation length (yr): 1

Dependency of single sp?: Unknown

Ecology and traits (narrative): Selenopids, commonly known as flatties or wall spiders, are wandering spiders usually found on walls or under rocks. Due to their flat habitus, they can hide inside narrow crevices. When disturbed, these spiders flee moving sideways to hide. Egg sacs of congeners are single and flat, paper-like and they are attached under bark where the female guards them (Dippenaar-Schoeman and Jocqué 1997, Crews 2011).

#### Threats

Justification for threats: No known threats.

##### Threats

Threat type: Past

Threats: 12. Other options - Other threat

#### Threats

Threat type: Past

Threats: 12. Other options - Other threat

#### Conservation

##### Conservation actions

#### Conservation actions

#### Other

##### Use and trade

Use type: International

Use and trade: 18. Unknown

##### Ecosystem services

Ecosystem service type: Very important

##### Research needed

Research needed: 1.1. Research - Taxonomy1.2. Research - Population size, distribution & trends1.3. Research - Life history & ecology1.5. Research - Threats

Justification for research needed: According to Gravely (1931), *S. shevaroyensis* resembles *S. radiatus.* Taxonomic clarification would be essential. Basic research is needed to know current distribution and population size and trends, ecology and traits of the species along with possible threats.

#### Use and trade

Use type: International

Use and trade: 18. Unknown

#### Ecosystem services

Ecosystem service type: Very important

#### Research needed

Research needed: 1.1. Research - Taxonomy1.2. Research - Population size, distribution & trends1.3. Research - Life history & ecology1.5. Research - Threats

Justification for research needed: According to Gravely (1931), *S. shevaroyensis* resembles *S. radiatus.* Taxonomic clarification would be essential. Basic research is needed to know current distribution and population size and trends, ecology and traits of the species along with possible threats.

#### Viability analysis

### Loxosceles devia

#### Species information

Scientific name: Loxosceles devia

Species authority: Gertsch & Mulaik, 1940

Common names: Texas recluse

Kingdom: Animalia

Phylum: Arthropoda

Class: Arachnida

Order: Araneae

Family: Sicariidae

Region for assessment: Global

#### Geographic range

Biogeographic realm: Nearctic

Countries: MexicoUnited States

Map of records (Google Earth): Suppl. material 5

Basis of EOO and AOO: Species Distribution Model

Basis (narrative): Given the relatively high number of records (Gertsch and Mulaik 1940, Gertsch 1958, Gertsch and Ennik 1983, GBIF.org 2018b), it was possible to perform species distribution modelling (see methods for details).

Min Elevation/Depth (m): 0

Max Elevation/Depth (m): 2030

Range description: This species is present near the Gulf of Mexico on Mexico and Texas, USA (Gertsch and Mulaik 1940, Gertsch 1958, Gertsch and Ennik 1983, GBIF.org 2018b).

#### New occurrences

#### Extent of occurrence

EOO (km2): 703860

Trend: Stable

Justification for trend: As it is a relatively widespread species with no known threats, able to live in different habitat types, we infer the trend to be stable.

Causes ceased?: Yes

Causes understood?: Yes

Causes reversible?: Yes

Extreme fluctuations?: No

#### Area of occupancy

Trend: Stable

Justification for trend: As it is a relatively widespread species with no known threats, able to live in different habitat types, we infer the trend to be stable.

Causes ceased?: Yes

Causes understood?: Yes

Causes reversible?: Yes

Extreme fluctuations?: No

AOO (km2): 385844

#### Locations

Number of locations: Not applicable

Justification for number of locations: No known threats to the species.

Trend: Stable

Extreme fluctuations?: No

#### Population

Number of individuals: Unknown

Trend: Stable

Justification for trend: As it is a relatively widespread species with no known threats, able to live in different habitat types, we infer the trend to be stable.

Causes ceased?: Yes

Causes understood?: Yes

Causes reversible?: Yes

Extreme fluctuations?: No

Population Information (Narrative): No population size estimates exist.

#### Subpopulations

Number of subpopulations: Unknown

Trend: Stable

Justification for trend: As it is a relatively widespread species with no known threats, able to live in different habitat types, we infer the trend to be stable.

Extreme fluctuations?: No

Severe fragmentation?: No

#### Habitat

System: Terrestrial

Habitat specialist: No

Habitat (narrative): This species thrives in arid, desert-like habitats in Texas and Mexico (Gertsch 1958, Gertsch and Ennik 1983, GBIF.org 2018b) commonly living under rocks, in caves, shrubland, sand dunes and occasionally in artificial habitats such as palm groves and road cuts (Gertsch and Mulaik 1940, Gertsch 1958, Gertsch and Ennik 1983, GBIF.org 2018b).

Trend in extent, area or quality?: Increase

Justification for trend: It preferred habitat, desert-like arid, is increasing in extent (United States Department of Agriculture 2003).

##### Habitat

Habitat importance: Major Importance

Habitats: 3.5. Shrubland - Subtropical/Tropical Dry7.1. Caves and Subterranean Habitats (non-aquatic) - Caves8.1. Desert - Hot

#### Habitat

Habitat importance: Major Importance

Habitats: 3.5. Shrubland - Subtropical/Tropical Dry7.1. Caves and Subterranean Habitats (non-aquatic) - Caves8.1. Desert - Hot

#### Ecology

Size: 5 - 10 mm

Generation length (yr): 1

Dependency of single sp?: No

Ecology and traits (narrative): The spiders of the genus *Loxosceles* are nocturnal ground-dwelling hunters that live under stones and other ground objects. Loxoscelids build a retreat with irregular webs. The webs of these species have been described as white, adhesive and flocculent. Many species in this genus have been reported to have strong venom since the haematoxins in the venom of *Loxosceles* destroys the cells of the skin after biting often resulting in necrosis (Gertsch 1958).

#### Threats

Justification for threats: No known threats.

##### Threats

Threat type: Ongoing

Threats: 12. Other options - Other threat

#### Threats

Threat type: Ongoing

Threats: 12. Other options - Other threat

#### Conservation

Justification for conservation actions: Many different nature reserves, wilderness areas, national parks and other protected lands fall within this species range (United Nations Environment World Conservation Monitoring Centre 2017).

##### Conservation actions

Conservation action type: In Place

Conservation actions: 1.1. Land/water protection - Site/area protection

#### Conservation actions

Conservation action type: In Place

Conservation actions: 1.1. Land/water protection - Site/area protection

#### Other

##### Use and trade

Use type: International

##### Ecosystem services

Ecosystem service type: Very important

##### Research needed

Research needed: 3.1. Monitoring - Population trends3.4. Monitoring - Habitat trends

Justification for research needed: Monitoring is needed to confirm inferred habitat and populations trends.

#### Use and trade

Use type: International

#### Ecosystem services

Ecosystem service type: Very important

#### Research needed

Research needed: 3.1. Monitoring - Population trends3.4. Monitoring - Habitat trends

Justification for research needed: Monitoring is needed to confirm inferred habitat and populations trends.

#### Viability analysis

### Heteropoda jiangxiensis

#### Species information

Scientific name: Heteropoda jiangxiensis

Species authority: Li, 1991

Kingdom: Animalia

Phylum: Arthropoda

Class: Arachnida

Order: Araneae

Family: Sparassidae

Region for assessment: Global

#### Geographic range

Biogeographic realm: Palearctic

Countries: China

Map of records (Google Earth): Suppl. material 6

Basis of EOO and AOO: Unknown

Basis (narrative): Unknown EOO or AOO.

Min Elevation/Depth (m): 160

Max Elevation/Depth (m): 160

Range description: This species is known only from the type locality in Jiangxi, China, recorded in 1989 (Li 1991).

#### New occurrences

#### Extent of occurrence

EOO (km2): Unknown

Trend: Unknown

Causes ceased?: Unknown

Causes understood?: Unknown

Causes reversible?: Unknown

Extreme fluctuations?: Unknown

#### Area of occupancy

Trend: Unknown

Causes ceased?: Unknown

Causes understood?: Unknown

Causes reversible?: Unknown

Extreme fluctuations?: Unknown

AOO (km2): Unknown

#### Locations

Number of locations: Unknown

Trend: Unknown

Extreme fluctuations?: Unknown

#### Population

Number of individuals: Unknown

Trend: Unknown

Causes ceased?: Unknown

Causes understood?: Unknown

Causes reversible?: Unknown

Extreme fluctuations?: Unknown

Population Information (Narrative): No population size estimates exist.

#### Subpopulations

Number of subpopulations: Unknown

Trend: Unknown

Extreme fluctuations?: Unknown

Severe fragmentation?: Unknown

#### Habitat

System: Terrestrial

Habitat specialist: Unknown

Habitat (narrative): The type locality falls in the region of tropical and subtropical moist broadleaf forest (Olson et al. 2001). Otherwise the preferred habitat of this species remains unknown.

Trend in extent, area or quality?: Unknown

##### Habitat

Habitat importance: Major Importance

Habitats: 18. Unknown

#### Habitat

Habitat importance: Major Importance

Habitats: 18. Unknown

#### Ecology

Size: 15.5 mm

Generation length (yr): 1

Dependency of single sp?: No

Ecology and traits (narrative): Heteropodids are nocturnal, wandering spiders which do not build webs but a retreat from silk (Song et al. 1999).

#### Threats

Justification for threats: Unknown threats.

##### Threats

Threat type: Past

Threats: 12. Other options - Other threat

#### Threats

Threat type: Past

Threats: 12. Other options - Other threat

#### Conservation

##### Conservation actions

#### Conservation actions

#### Other

##### Use and trade

Use type: International

##### Ecosystem services

Ecosystem service type: Very important

##### Research needed

Research needed: 1.2. Research - Population size, distribution & trends1.3. Research - Life history & ecology1.5. Research - Threats

Justification for research needed: Basic research is needed to know the current distribution and population size and trends, ecology and traits of the species along with possible threats.

#### Use and trade

Use type: International

#### Ecosystem services

Ecosystem service type: Very important

#### Research needed

Research needed: 1.2. Research - Population size, distribution & trends1.3. Research - Life history & ecology1.5. Research - Threats

Justification for research needed: Basic research is needed to know the current distribution and population size and trends, ecology and traits of the species along with possible threats.

#### Viability analysis

### Isopeda echuca

#### Species information

Scientific name: Isopeda echuca

Species authority: Hirst, 1992

Kingdom: Animalia

Phylum: Arthropoda

Class: Arachnida

Order: Araneae

Family: Sparassidae

Region for assessment: Global

#### Geographic range

Biogeographic realm: Australasian

Countries: Australia

Map of records (Google Earth): Suppl. material 7

Basis of EOO and AOO: Species Distribution Model

Basis (narrative): Given the relatively high number of records (Hirst 1992), it was possible to perform species distribution modelling (see methods for details).

Min Elevation/Depth (m): 70

Max Elevation/Depth (m): 450

Range description: This species is known from New South Wales and Victoria in Australia and the latest known record date was in 1988 (Hirst 1992).

#### New occurrences

#### Extent of occurrence

EOO (km2): 118216

Trend: Decline (inferred)

Justification for trend: There is decline in habitat quality with consequent inferred decline in EOO as there has been reported decline in the rainfall and increase in the daily maximum temperature within the species range (Murphy and Timbal 2008).

Causes ceased?: No

Causes understood?: Yes

Causes reversible?: No

Extreme fluctuations?: No

#### Area of occupancy

Trend: Decline (inferred)

Justification for trend: There is decline in habitat quality with consequent inferred decline in AOO as there has been reported decline in the rainfall and increase in the daily maximum temperature within the species range (Murphy and Timbal 2008).

Causes ceased?: No

Causes understood?: Yes

Causes reversible?: No

Extreme fluctuations?: No

AOO (km2): 96180

#### Locations

Number of locations: Unknown

Trend: Decline (inferred)

Justification for trend: There is decline in habitat quality with consequent inferred decline in number of locations as there has been reported decline in the rainfall and increase in the daily maximum temperature within the species range (Murphy and Timbal 2008).

Extreme fluctuations?: No

#### Population

Number of individuals: Unknown

Trend: Decline (inferred)

Justification for trend: Inferred from decline in AOO and habitat quality.

Causes ceased?: No

Causes understood?: Yes

Causes reversible?: No

Extreme fluctuations?: No

Population Information (Narrative): No population size estimates exist.

#### Subpopulations

Number of subpopulations: Unknown

Trend: Decline (inferred)

Justification for trend: There is decline in habitat quality with consequent inferred decline in number of subpopulations as there has been reported decline in the rainfall and increase in the daily maximum temperature within the species range (Murphy and Timbal 2008).

Extreme fluctuations?: No

Severe fragmentation?: Unknown

#### Habitat

System: Terrestrial

Habitat specialist: Unknown

Habitat (narrative): *Isopeda* seems to be present only in areas with rainfall above 500 mm. One of the records was reported from Hardings swamp (Hirst 1992).

Trend in extent, area or quality?: Decline (inferred)

Justification for trend: There is decline in habitat quality as there has been reported decline in the rainfall and increase in the daily maximum temperature (Murphy and Timbal 2008).

##### Habitat

Habitat importance: Major Importance

Habitats: 5.4. Wetlands (inland) - Bogs, Marshes, Swamps, Fens, Peatlands

#### Habitat

Habitat importance: Major Importance

Habitats: 5.4. Wetlands (inland) - Bogs, Marshes, Swamps, Fens, Peatlands

#### Ecology

Size: 21.3 - 28.55 mm

Generation length (yr): 1

Dependency of single sp?: No

Ecology and traits (narrative): Males of *I. echuca* have been observed from August to June, although it has been suggested both males and females may be present throughout the year and the existing gaps may be due to insufficient sampling and inactivity of the spiders (Hirst 1992). Spiders of the family Sparassidae in general are nocturnal and wandering hunters living on the soil surface or on plants (Jocqué and Dippenaar-Schoeman 2006).

#### Threats

Justification for threats: Since this species seems to prefer moist habitats, the ongoing climate change is affecting the habitat quality as there has been reported decline in the rainfall and daily maximum temperatures are rising (Murphy and Timbal 2008).

##### Threats

Threat type: Ongoing

Threats: 11.2. Climate change & severe weather - Droughts

#### Threats

Threat type: Ongoing

Threats: 11.2. Climate change & severe weather - Droughts

#### Conservation

Justification for conservation actions: At least part of the range of this species is inside protected areas, namely Barmah National Park and Heathcote-Graytown National Park (United Nations Environment World Conservation Monitoring Centre 2017). Given the effects of the ongoing climate change, it would be essential to manage the sites where this species is known to occur.

##### Conservation actions

Conservation action type: In Place

Conservation actions: 1.1. Land/water protection - Site/area protection1.2. Land/water protection - Resource & habitat protection

##### Conservation actions

Conservation action type: Needed

Conservation actions: 2.1. Land/water management - Site/area management

#### Conservation actions

Conservation action type: In Place

Conservation actions: 1.1. Land/water protection - Site/area protection1.2. Land/water protection - Resource & habitat protection

#### Conservation actions

Conservation action type: Needed

Conservation actions: 2.1. Land/water management - Site/area management

#### Other

##### Use and trade

Use type: International

##### Ecosystem services

Ecosystem service type: Very important

##### Research needed

Research needed: 2.2. Conservation Planning - Area-based Management Plan3.1. Monitoring - Population trends3.4. Monitoring - Habitat trends

Justification for research needed: Monitoring is needed to confirm population and habitat trends and also conservation planning could take place given the continuing decline in habitat quality.

#### Use and trade

Use type: International

#### Ecosystem services

Ecosystem service type: Very important

#### Research needed

Research needed: 2.2. Conservation Planning - Area-based Management Plan3.1. Monitoring - Population trends3.4. Monitoring - Habitat trends

Justification for research needed: Monitoring is needed to confirm population and habitat trends and also conservation planning could take place given the continuing decline in habitat quality.

#### Viability analysis

### Pseudopoda parvipunctata

#### Species information

Scientific name: Pseudopoda parvipunctata

Species authority: Jäger, 2001

Kingdom: Animalia

Phylum: Arthropoda

Class: Arachnida

Order: Araneae

Family: Sparassidae

Region for assessment: Global

#### Geographic range

Biogeographic realm: Indomalayan

Countries: Thailand

Map of records (Google Earth): Suppl. material 8

Basis of EOO and AOO: Unknown

Basis (narrative): Unknown EOO or AOO.

Min Elevation/Depth (m): 500

Max Elevation/Depth (m): 1460

Range description: This species is known from only two localities, Doi Suthep and Doi Pui in Thailand, both recorded in 1986 (Jäger 2001).

#### New occurrences

#### Extent of occurrence

EOO (km2): Unknown

Trend: Unknown

Causes ceased?: Unknown

Causes understood?: Unknown

Causes reversible?: Unknown

Extreme fluctuations?: Unknown

#### Area of occupancy

Trend: Unknown

Causes ceased?: Unknown

Causes understood?: Unknown

Causes reversible?: Unknown

Extreme fluctuations?: Unknown

AOO (km2): Unknown

#### Locations

Number of locations: Unknown

Trend: Unknown

Extreme fluctuations?: Unknown

#### Population

Number of individuals: Unknown

Trend: Unknown

Causes ceased?: Unknown

Causes understood?: Unknown

Causes reversible?: Unknown

Extreme fluctuations?: Unknown

Population Information (Narrative): No population size estimates exist.

#### Subpopulations

Number of subpopulations: Unknown

Trend: Unknown

Extreme fluctuations?: Unknown

Severe fragmentation?: Unknown

#### Habitat

System: Terrestrial

Habitat specialist: Unknown

Habitat (narrative): There is no recorded habitat data for this species. The localities fall somewhere between tropical and subtropical moist broadeaf forest and tropical and subtropical dry broadleaf forest areas (Olson et al. 2001).

Trend in extent, area or quality?: Unknown

##### Habitat

Habitat importance: Major Importance

Habitats: 18. Unknown

#### Habitat

Habitat importance: Major Importance

Habitats: 18. Unknown

#### Ecology

Size: 5.0 - 10.2mm

Generation length (yr): 1

Dependency of single sp?: Unknown

Ecology and traits (narrative): Spiders of the family Sparassidae, in general, are nocturnal and wandering spiders live on the soil surface or on plants. They are sometimes also found in caves (Jocqué and Dippenaar-Schoeman 2006).

#### Threats

Justification for threats: Unknown threats.

##### Threats

Threat type: Past

Threats: 12. Other options - Other threat

#### Threats

Threat type: Past

Threats: 12. Other options - Other threat

#### Conservation

Justification for conservation actions: There are only few records for this species, however, they seem to be within protected areas or, at least, near protected areas in Thailand, e.g. Salawin Wildlife Sancturary (United Nations Environment World Conservation Monitoring Centre 2017).

##### Conservation actions

Conservation action type: In Place

Conservation actions: 1.1. Land/water protection - Site/area protection1.2. Land/water protection - Resource & habitat protection

#### Conservation actions

Conservation action type: In Place

Conservation actions: 1.1. Land/water protection - Site/area protection1.2. Land/water protection - Resource & habitat protection

#### Other

##### Use and trade

Use type: International

##### Ecosystem services

Ecosystem service type: Very important

##### Research needed

Research needed: 1.2. Research - Population size, distribution & trends1.3. Research - Life history & ecology1.5. Research - Threats

Justification for research needed: Basic research is needed to know the current distribution, population trends, habitat fidelity of the species and possible threats.

#### Use and trade

Use type: International

#### Ecosystem services

Ecosystem service type: Very important

#### Research needed

Research needed: 1.2. Research - Population size, distribution & trends1.3. Research - Life history & ecology1.5. Research - Threats

Justification for research needed: Basic research is needed to know the current distribution, population trends, habitat fidelity of the species and possible threats.

#### Viability analysis

### Sinopoda sitkao

#### Species information

Scientific name: Sinopoda sitkao

Species authority: Jäger, 2012

Kingdom: Animalia

Phylum: Arthropoda

Class: Arachnida

Order: Araneae

Family: Sparassidae

Region for assessment: Global

#### Geographic range

Biogeographic realm: Indomalayan

Countries: Lao People's Democratic Republic

Map of records (Google Earth): Suppl. material 9

Basis of EOO and AOO: Unknown

Basis (narrative): Unknown EOO or AOO.

Min Elevation/Depth (m): 430

Max Elevation/Depth (m): 430

Range description: Known only from the type locality in Luang Prabang Province, Laos, recorded in 2012 (Jäger 2012).

#### New occurrences

#### Extent of occurrence

EOO (km2): Unknown

Trend: Unknown

Causes ceased?: Unknown

Causes understood?: Unknown

Causes reversible?: Unknown

Extreme fluctuations?: Unknown

#### Area of occupancy

Trend: Unknown

Causes ceased?: Unknown

Causes understood?: Unknown

Causes reversible?: Unknown

Extreme fluctuations?: Unknown

AOO (km2): Unknown

#### Locations

Number of locations: Unknown

Trend: Unknown

Extreme fluctuations?: Unknown

#### Population

Number of individuals: Unknown

Trend: Unknown

Causes ceased?: Unknown

Causes understood?: Unknown

Causes reversible?: Unknown

Extreme fluctuations?: Unknown

#### Subpopulations

Number of subpopulations: Unknown

Trend: Unknown

Extreme fluctuations?: Unknown

Severe fragmentation?: Unknown

#### Habitat

System: Terrestrial

Habitat specialist: Yes

Habitat (narrative): A single specimen was found in a limestone cave (Jäger 2012) and we assume the species to be exclusive to this habitat type.

Trend in extent, area or quality?: Unknown

##### Habitat

Habitat importance: Major Importance

Habitats: 7.1. Caves and Subterranean Habitats (non-aquatic) - Caves

#### Habitat

Habitat importance: Major Importance

Habitats: 7.1. Caves and Subterranean Habitats (non-aquatic) - Caves

#### Ecology

Size: 15.6 mm

Generation length (yr): 0

Dependency of single sp?: Unknown

Ecology and traits (narrative): This is a pale-coloured species whose single specimen was found from a cave in limestone (Jäger 2012).

#### Threats

Justification for threats: Unknown threats.

##### Threats

Threat type: Past

Threats: 12. Other options - Other threat

#### Threats

Threat type: Past

Threats: 12. Other options - Other threat

#### Conservation

##### Conservation actions

#### Conservation actions

#### Other

##### Use and trade

Use type: International

Use and trade: 18. Unknown

##### Ecosystem services

Ecosystem service type: Very important

##### Research needed

Research needed: 1.2. Research - Population size, distribution & trends1.3. Research - Life history & ecology1.5. Research - Threats

Justification for research needed: Basic research is needed to know the current distribution and population size and trends, ecology and traits of the species, along with possible threats.

#### Use and trade

Use type: International

Use and trade: 18. Unknown

#### Ecosystem services

Ecosystem service type: Very important

#### Research needed

Research needed: 1.2. Research - Population size, distribution & trends1.3. Research - Life history & ecology1.5. Research - Threats

Justification for research needed: Basic research is needed to know the current distribution and population size and trends, ecology and traits of the species, along with possible threats.

#### Viability analysis

### Tetrablemma brevidens

#### Species information

Scientific name: Tetrablemma brevidens

Species authority: Tong & Li, 2008

Kingdom: Animalia

Phylum: Arthropoda

Class: Arachnida

Order: Araneae

Family: Tetrablemmidae

Region for assessment: Global

#### Geographic range

Biogeographic realm: Palearctic

Countries: China

Map of records (Google Earth): Suppl. material 10

Basis of EOO and AOO: Unknown

Basis (narrative): Unknown EOO or AOO.

Min Elevation/Depth (m): 70

Max Elevation/Depth (m): 160

Range description: This species is known from only two localities in western Hainan, China, both recorded in 2005 (Tong 2013, Tong and Li 2008).

#### New occurrences

#### Extent of occurrence

EOO (km2): Unknown

Trend: Unknown

Causes ceased?: Unknown

Causes understood?: Unknown

Causes reversible?: Unknown

Extreme fluctuations?: Unknown

#### Area of occupancy

Trend: Unknown

Causes ceased?: Unknown

Causes understood?: Unknown

Causes reversible?: Unknown

Extreme fluctuations?: Unknown

AOO (km2): Unknown

#### Locations

Number of locations: Unknown

Trend: Unknown

Extreme fluctuations?: Unknown

#### Population

Number of individuals: Unknown

Trend: Unknown

Causes ceased?: Unknown

Causes understood?: Unknown

Causes reversible?: Unknown

Extreme fluctuations?: Unknown

Population Information (Narrative): No population size estimates exist.

#### Subpopulations

Number of subpopulations: Unknown

Trend: Unknown

Extreme fluctuations?: Unknown

Severe fragmentation?: Unknown

#### Habitat

System: Terrestrial

Habitat specialist: Yes

Habitat (narrative): This species was found only from caves (Tong 2013, Tong and Li 2008) and we assume it to be exclusive to this habitat type.

Trend in extent, area or quality?: Unknown

##### Habitat

Habitat importance: Major Importance

Habitats: 7.1. Caves and Subterranean Habitats (non-aquatic) - Caves

#### Habitat

Habitat importance: Major Importance

Habitats: 7.1. Caves and Subterranean Habitats (non-aquatic) - Caves

#### Ecology

Size: 1.19 - 1.28 mm

Generation length (yr): 0

Dependency of single sp?: Unknown

Ecology and traits (narrative): Members of the family Tetrablemmidae are small to tiny spiders known as “armored spiders” due to their heavily scleritised bodies. These three-clawed, ecribellate, haplogyne spiders are covered with a hardened shell or abdominal scutae (ventral, dorsal and lateral) hinged with softer material allowing expansion between the plates in a bellows-like way (Lehtinen 1981; Labarque and Grismado 2009; Whyte and Anderson 2017). They are typically found in tropical and semitropical habitats, although a species of *Shearella* has been found living in dry coastal habitats (Lehtinen 1981). Very little is known about their behaviour although Burger et al. (2006) recorded the first observation of tetrablemmid spiders mating in Thailand and the web and egg-sac construction of *Brignoliella vulgaris* have been observed (Lehtinen 1981). Tetrablemmid spiders have been collected from moss and leaf litter; under stones, bark or logs; in soil samples including hanging soils such as in orchids and epiphytes as well as in dark caves (Tong and Li 2008). Some cave and soil dwelling species have reduced number of eyes such as in the genus *Tetrablemma* which have only four (Whyte and Anderson 2017). The cave dwelling species *Tetrablemma brevidens* is part of the first report of tetrablemmid spiders recorded from China found on Hainan Island by Tong and Li (2008).

#### Threats

Justification for threats: Unknown threats.

##### Threats

Threat type: Past

Threats: 12. Other options - Other threat

#### Threats

Threat type: Past

Threats: 12. Other options - Other threat

#### Conservation

Justification for conservation actions: At least one locality seems to be partly within Jiaxi Nature Reserve (United Nations Environment World Conservation Monitoring Centre 2017).

##### Conservation actions

Conservation action type: In Place

Conservation actions: 1.1. Land/water protection - Site/area protection1.2. Land/water protection - Resource & habitat protection

#### Conservation actions

Conservation action type: In Place

Conservation actions: 1.1. Land/water protection - Site/area protection1.2. Land/water protection - Resource & habitat protection

#### Other

##### Use and trade

Use type: International

Use and trade: 18. Unknown

##### Ecosystem services

Ecosystem service type: Very important

##### Research needed

Research needed: 1.2. Research - Population size, distribution & trends1.3. Research - Life history & ecology1.5. Research - Threats

Justification for research needed: Basic research is needed to know the current distribution and population size and trends, ecology and traits of the species, along with possible threats.

#### Use and trade

Use type: International

Use and trade: 18. Unknown

#### Ecosystem services

Ecosystem service type: Very important

#### Research needed

Research needed: 1.2. Research - Population size, distribution & trends1.3. Research - Life history & ecology1.5. Research - Threats

Justification for research needed: Basic research is needed to know the current distribution and population size and trends, ecology and traits of the species, along with possible threats.

#### Viability analysis

### Chrysometa lepida

#### Species information

Scientific name: Chrysometa lepida

Species authority: (Keyserling, 1881)

Kingdom: Animalia

Phylum: Arthropoda

Class: Arachnida

Order: Araneae

Family: Tetragnathidae

Region for assessment: Global

#### Geographic range

Biogeographic realm: Neotropical

Countries: Peru

Map of records (Google Earth): Suppl. material 11

Basis of EOO and AOO: Unknown

Basis (narrative): Unknown EOO or AOO.

Min Elevation/Depth (m): 3060

Max Elevation/Depth (m): 3060

Range description: Known only from the type locality in Tarma, Peru, recorded once prior to 1881 (Keyserling 1881).

#### New occurrences

#### Extent of occurrence

EOO (km2): Unknown

Trend: Unknown

Causes ceased?: Unknown

Causes understood?: Unknown

Causes reversible?: Unknown

Extreme fluctuations?: Unknown

#### Area of occupancy

Trend: Unknown

Causes ceased?: Unknown

Causes understood?: Unknown

Causes reversible?: Unknown

Extreme fluctuations?: Unknown

AOO (km2): Unknown

#### Locations

Number of locations: Unknown

Trend: Unknown

Extreme fluctuations?: Unknown

#### Population

Number of individuals: Unknown

Trend: Unknown

Causes ceased?: Unknown

Causes understood?: Unknown

Causes reversible?: Unknown

Extreme fluctuations?: Unknown

Population Information (Narrative): No population size estimates exist.

#### Subpopulations

Number of subpopulations: Unknown

Trend: Unknown

Extreme fluctuations?: Unknown

Severe fragmentation?: Unknown

#### Habitat

System: Terrestrial

Habitat specialist: Unknown

Habitat (narrative): The preferred habitat of this species is unknown, although congeners tend to live at high altitudes (Andes and paramos; both areas need more exploration and collection of specimens) (Levi 1986). The habitats around Tarma are dry montane grasslands and shrublands, deserts and xeric shrublands (Olson et al. 2001).

Trend in extent, area or quality?: Unknown

##### Habitat

Habitat importance: Major Importance

Habitats: 18. Unknown

#### Habitat

Habitat importance: Major Importance

Habitats: 18. Unknown

#### Ecology

Size: 2.3 mm

Generation length (yr): 1

Dependency of single sp?: Unknown

Ecology and traits (narrative): Spiders of the family Tetragnathidae are orb-weavers building a web with often only few radii and spirals (Álvarez-Padilla 2007). Some species build their webs horizontally above water (Jocqué and Dippenaar-Schoeman 2006) and commonly they have been observed to build their webs near water sources, along river marigins for instance. Spiders of this family are usually found in the centre of the web or in the vegetation near the web where they tend to hide.

#### Threats

Justification for threats: Unknown threats.

##### Threats

Threat type: Past

Threats: 12. Other options - Other threat

#### Threats

Threat type: Past

Threats: 12. Other options - Other threat

#### Conservation

##### Conservation actions

#### Conservation actions

#### Other

##### Use and trade

Use type: International

Use and trade: 18. Unknown

##### Ecosystem services

Ecosystem service type: Very important

##### Research needed

Research needed: 1.2. Research - Population size, distribution & trends1.3. Research - Life history & ecology1.5. Research - Threats

Justification for research needed: Basic research is needed to know the current distribution and population size and trends, ecology and traits of the species, along with possible threats.

#### Use and trade

Use type: International

Use and trade: 18. Unknown

#### Ecosystem services

Ecosystem service type: Very important

#### Research needed

Research needed: 1.2. Research - Population size, distribution & trends1.3. Research - Life history & ecology1.5. Research - Threats

Justification for research needed: Basic research is needed to know the current distribution and population size and trends, ecology and traits of the species, along with possible threats.

#### Viability analysis

### Cyrtognatha pachygnathoides

#### Species information

Scientific name: Cyrtognatha pachygnathoides

Species authority: (O. Pickard-Cambridge, 1894)

Kingdom: Animalia

Phylum: Arthropoda

Class: Arachnida

Order: Araneae

Family: Tetragnathidae

Region for assessment: Global

#### Geographic range

Biogeographic realm: Neotropical

Countries: PanamaCosta Rica

Map of records (Google Earth): Suppl. material 12

Basis of EOO and AOO: Species Distribution Model

Basis (narrative): Given the reasonable number of records (Pickard-Cambridge 1894, Dimitrov and Hormiga 2009a, GBIF.org 2018a), it was possible to perform species distribution modelling (see methods for details).

Min Elevation/Depth (m): 1010

Max Elevation/Depth (m): 3730

Range description: This species is present in Costa Rica and Panama. According to Dimitrov and Hormiga (2009a), the genus *Cyrtognatha* is widespread in South America, Central America and the southern parts of North America, although the majority of the species are known from single localities. The latest known record is from 1995, from La Amistad International Park in Panama (Dimitrov and Hormiga 2009a). Since there are only few data from a limited number of localities on this species, its range might be relatively narrow as predicted by the models (Dimitar Dimitrov pers. comm.).

#### New occurrences

#### Extent of occurrence

EOO (km2): 7829

Trend: Unknown

Causes ceased?: Unknown

Causes understood?: Unknown

Causes reversible?: Unknown

Extreme fluctuations?: Unknown

#### Area of occupancy

Trend: Unknown

Causes ceased?: Unknown

Causes understood?: Unknown

Causes reversible?: Unknown

Extreme fluctuations?: Unknown

AOO (km2): 4184

#### Locations

Number of locations: Unknown

Trend: Unknown

Extreme fluctuations?: Unknown

#### Population

Number of individuals: Unknown

Trend: Unknown

Causes ceased?: Unknown

Causes understood?: Unknown

Causes reversible?: Unknown

Extreme fluctuations?: Unknown

Population Information (Narrative): Population size and trend are unknown.

#### Subpopulations

Number of subpopulations: Unknown

Trend: Unknown

Extreme fluctuations?: Unknown

Severe fragmentation?: Unknown

#### Habitat

System: Terrestrial

Habitat specialist: Unknown

Habitat (narrative): This species seems to prefer tropical cloud and lowland rainforests (Dimitrov and Hormiga 2009a).

Trend in extent, area or quality?: Unknown

##### Habitat

Habitat importance: Major Importance

Habitats: 1.6. Forest - Subtropical/Tropical Moist Lowland

#### Habitat

Habitat importance: Major Importance

Habitats: 1.6. Forest - Subtropical/Tropical Moist Lowland

#### Ecology

Size: 4.9 mm

Generation length (yr): 1

Dependency of single sp?: Unknown

Ecology and traits (narrative): *Cyrtognatha* species build horizontal or, in some cases, also vertical orb webs. There are usually only few radii and spiral turns in the web and also an open hub where the spider often sits. They flee from their web if disturbed and then hide in the vegetation (Dimitrov and Hormiga 2009b).

#### Threats

Justification for threats: The major potential threat to this species could be habitat destruction such as deforestation and aridification due to climate change (Dimitar Dimitrov pers. comm.).

##### Threats

Threat type: Ongoing

Threats: 11.1. Climate change & severe weather - Habitat shifting & alteration11.2. Climate change & severe weather - Droughts5.3. Biological resource use - Logging & wood harvesting

#### Threats

Threat type: Ongoing

Threats: 11.1. Climate change & severe weather - Habitat shifting & alteration11.2. Climate change & severe weather - Droughts5.3. Biological resource use - Logging & wood harvesting

#### Conservation

Justification for conservation actions: At least part of the range of this species is inside protected areas since it has been recorded from Volcan Chiriqui inside Volcan Baru National Park (Pickard-Cambridge 1894) and La Amistad International Park (Dimitrov and Hormiga 2009a) which covers an area of 570,045 ha (UNESCO 2017). However, it is not known if the species is effectively protected since there are no follow-up collections or monitoring (Dimitar Dimitrov pers. comm.).

##### Conservation actions

Conservation action type: In Place

Conservation actions: 1.1. Land/water protection - Site/area protection1.2. Land/water protection - Resource & habitat protection

#### Conservation actions

Conservation action type: In Place

Conservation actions: 1.1. Land/water protection - Site/area protection1.2. Land/water protection - Resource & habitat protection

#### Other

##### Use and trade

Use type: International

##### Ecosystem services

Ecosystem service type: Very important

##### Research needed

Research needed: 3.1. Monitoring - Population trends3.4. Monitoring - Habitat trends

Justification for research needed: Monitoring is needed to know the current population and habitat trends.

#### Use and trade

Use type: International

#### Ecosystem services

Ecosystem service type: Very important

#### Research needed

Research needed: 3.1. Monitoring - Population trends3.4. Monitoring - Habitat trends

Justification for research needed: Monitoring is needed to know the current population and habitat trends.

#### Viability analysis

### Brachionopus tristis

#### Species information

Scientific name: Brachionopus tristis

Species authority: Purcell, 1903

Kingdom: Animalia

Phylum: Arthropoda

Class: Arachnida

Order: Araneae

Family: Theraphosidae

Region for assessment: Global

#### Geographic range

Biogeographic realm: Afrotropical

Countries: South Africa

Map of records (Google Earth): Suppl. material 13

Basis of EOO and AOO: Unknown

Basis (narrative): EOO and AOO are unknown.

Min Elevation/Depth (m): 820

Max Elevation/Depth (m): 820

Range description: Known only from the type locality in Barberton, Transvaal, South Africa, recorded once in 1897 (Purcell 1903).

#### New occurrences

#### Extent of occurrence

EOO (km2): Unknown

Trend: Unknown

Causes ceased?: Unknown

Causes understood?: Unknown

Causes reversible?: Unknown

Extreme fluctuations?: Unknown

#### Area of occupancy

Trend: Unknown

Causes ceased?: Unknown

Causes understood?: Unknown

Causes reversible?: Unknown

Extreme fluctuations?: Unknown

AOO (km2): Unknown

#### Locations

Number of locations: Unknown

Trend: Unknown

Extreme fluctuations?: Unknown

#### Population

Number of individuals: Unknown

Trend: Unknown

Causes ceased?: Unknown

Causes understood?: Unknown

Causes reversible?: Unknown

Extreme fluctuations?: Unknown

Population Information (Narrative): Population size and trend are unknown.

#### Subpopulations

Number of subpopulations: Unknown

Trend: Unknown

Extreme fluctuations?: Unknown

Severe fragmentation?: Unknown

#### Habitat

System: Terrestrial

Habitat specialist: Unknown

Habitat (narrative): There is no recorded habitat data available. The type locality falls into the ecoregion of tropical and subtropical grasslands, savannahs and shrublands (Olson et al. 2001).

Trend in extent, area or quality?: Unknown

##### Habitat

Habitat importance: Major Importance

Habitats: 18. Unknown

#### Habitat

Habitat importance: Major Importance

Habitats: 18. Unknown

#### Ecology

Size: 13.5 mm

Generation length (yr): 4

Dependency of single sp?: Unknown

Ecology and traits (narrative): Spiders of the family Theraphosidae are free-living, ground-dwelling spiders. They build a burrow lined with silk or hide in a retreat under a rock (Jocqué and Dippenaar-Schoeman 2006). Theraphosid males do not moult when they reach maturity and they also have a shorter lifespan compared to females (Costa and Pérez-Miles 2002). Burrows often have their own two chambers: one is for the spider to moult and the other to eat and rest (Locht et al. 1999).

#### Threats

Justification for threats: Unknown threats.

##### Threats

Threat type: Past

Threats: 12. Other options - Other threat

#### Threats

Threat type: Past

Threats: 12. Other options - Other threat

#### Conservation

##### Conservation actions

#### Conservation actions

#### Other

##### Use and trade

Use type: International

##### Ecosystem services

Ecosystem service type: Very important

##### Research needed

Research needed: 1.2. Research - Population size, distribution & trends1.3. Research - Life history & ecology1.5. Research - Threats

Justification for research needed: Basic research is needed to know the current distribution and population size and trends, ecology and traits of the species, along with possible threats.

#### Use and trade

Use type: International

#### Ecosystem services

Ecosystem service type: Very important

#### Research needed

Research needed: 1.2. Research - Population size, distribution & trends1.3. Research - Life history & ecology1.5. Research - Threats

Justification for research needed: Basic research is needed to know the current distribution and population size and trends, ecology and traits of the species, along with possible threats.

#### Viability analysis

### Cardiopelma mascatum

#### Species information

Scientific name: Cardiopelma mascatum

Species authority: Vol, 1999

Kingdom: Animalia

Phylum: Arthropoda

Class: Arachnida

Order: Araneae

Family: Theraphosidae

Region for assessment: Global

#### Geographic range

Biogeographic realm: Neotropical

Countries: Mexico

Map of records (Google Earth): Suppl. material 14

Basis of EOO and AOO: Unknown

Basis (narrative): Unknown EOO and AOO.

Min Elevation/Depth (m): 1960

Max Elevation/Depth (m): 1960

Range description: This species has been recorded only once from unspecified locality in Mexico prior to 1999 (Vol 1999). Although the geographical origin of this species is not mentioned in the bibliography, this genus has been broadly trafficked for the pet trade from Mexico into Europe and Northern America (the type specimen itself was reared in captivity). Only known to the scientific world from a single female moult, this monogeneric genus appears to hold several undescribed species, which have not yet been scientifically analysed and whose range cannot be mapped. It is therefore impossible at this stage in our knowledge to assess the distribution range of this particular species, although the fact that it has not been scientifically analysed but appears in the pet trade might indicate it does not inhabit remote localities but is rather highly localised. It is possibly threatened with deforestation and illegal trade.

#### New occurrences

#### Extent of occurrence

EOO (km2): Unknown

Trend: Unknown

Causes ceased?: Unknown

Causes understood?: Unknown

Causes reversible?: Unknown

Extreme fluctuations?: Unknown

#### Area of occupancy

Trend: Unknown

Causes ceased?: Unknown

Causes understood?: Unknown

Causes reversible?: Unknown

Extreme fluctuations?: Unknown

AOO (km2): Unknown

#### Locations

Number of locations: Unknown

Trend: Unknown

Extreme fluctuations?: Unknown

#### Population

Number of individuals: Unknown

Trend: Unknown

Causes ceased?: Unknown

Causes understood?: Unknown

Causes reversible?: Unknown

Extreme fluctuations?: Unknown

Population Information (Narrative): Population size and trend are unknown.

#### Subpopulations

Number of subpopulations: Unknown

Trend: Unknown

Extreme fluctuations?: Unknown

Severe fragmentation?: Unknown

#### Habitat

System: Terrestrial

Habitat specialist: Unknown

Habitat (narrative): There is a variety of tropical and subtropical forest types in Mexico along with deserts and xeric shrublands (Olson et al. 2001). Since the type locality is unspecified, the suitable habitat preferred by this species cannot be inferred.

Trend in extent, area or quality?: Unknown

##### Habitat

Habitat importance: Major Importance

Habitats: 18. Unknown

#### Habitat

Habitat importance: Major Importance

Habitats: 18. Unknown

#### Ecology

Size: 28 mm

Generation length (yr): 4

Dependency of single sp?: Unknown

Ecology and traits (narrative): Spiders of the family Theraphosidae are free-living, ground-dwelling spiders. They build a burrow lined with silk or hide in a retreat under a rock (Jocqué and Dippenaar-Schoeman 2006). Theraphosid males do not moult when they reach maturity and they also have shorter lifespans compared to females (Costa and Pérez-Miles 2002). The burrow might have two chambers: one is for the spider to moult and the other to eat and rest (Locht et al. 1999).

#### Threats

Justification for threats: This genus has been broadly trafficked for the pet trade from Mexico into Europe and Northern America and therefore collection as a pet may be a threat to this particular species as well.

##### Threats

Threat type: Ongoing

Threats: 5.1.1. Biological resource use - Hunting & trapping terrestrial animals - Intentional use (species is the target)

#### Threats

Threat type: Ongoing

Threats: 5.1.1. Biological resource use - Hunting & trapping terrestrial animals - Intentional use (species is the target)

#### Conservation

Justification for conservation actions: It would be essential to raise awareness and communication of the consequences of trade on exotic animals captured from the wild.

##### Conservation actions

Conservation action type: Needed

Conservation actions: 4.3. Education & awareness - Awareness & communications

#### Conservation actions

Conservation action type: Needed

Conservation actions: 4.3. Education & awareness - Awareness & communications

#### Other

Justification for use and trade: This species is on the pet trade at an international level.

##### Use and trade

Use type: International

Use and trade: 13. Pets/display animals, horticulture

##### Ecosystem services

Ecosystem service type: Very important

##### Research needed

Research needed: 1.2. Research - Population size, distribution & trends1.3. Research - Life history & ecology1.5. Research - Threats

Justification for research needed: Basic research is needed to know the current distribution and population size and trends, ecology and traits of the species, along with possible threats besides the pet trade.

#### Use and trade

Use type: International

Use and trade: 13. Pets/display animals, horticulture

#### Ecosystem services

Ecosystem service type: Very important

#### Research needed

Research needed: 1.2. Research - Population size, distribution & trends1.3. Research - Life history & ecology1.5. Research - Threats

Justification for research needed: Basic research is needed to know the current distribution and population size and trends, ecology and traits of the species, along with possible threats besides the pet trade.

#### Viability analysis

### Cyriopagopus vonwirthi

#### Species information

Scientific name: Cyriopagopus vonwirthi

Species authority: (Schmidt, 2005)

Kingdom: Animalia

Phylum: Arthropoda

Class: Arachnida

Order: Araneae

Family: Theraphosidae

Taxonomic notes: Species transferred from *Haplopelma* to *Cyriopagopus* after genus synonymy (Smith & Jacobi 2015).

Region for assessment: Global

#### Geographic range

Biogeographic realm: Indomalayan

Countries: Viet Nam

Map of records (Google Earth): Suppl. material 15

Basis of EOO and AOO: Unknown

Basis (narrative): Unknown EOO or AOO.

Min Elevation/Depth (m): 0

Max Elevation/Depth (m): 0

Range description: This species was collected from the pet trade, confirmed from an unspecified locality in Vietnam with no date (Schmidt 2005).

#### New occurrences

#### Extent of occurrence

EOO (km2): Unknown

Trend: Unknown

Causes ceased?: Unknown

Causes understood?: Unknown

Causes reversible?: Unknown

Extreme fluctuations?: Unknown

#### Area of occupancy

Trend: Unknown

Causes ceased?: Unknown

Causes understood?: Unknown

Causes reversible?: Unknown

Extreme fluctuations?: Unknown

AOO (km2): Unknown

#### Locations

Number of locations: Unknown

Trend: Unknown

Extreme fluctuations?: Unknown

#### Population

Number of individuals: Unknown

Trend: Unknown

Causes ceased?: Unknown

Causes understood?: Unknown

Causes reversible?: Unknown

Extreme fluctuations?: Unknown

Population Information (Narrative): No population size estimates exist.

#### Subpopulations

Number of subpopulations: Unknown

Trend: Unknown

Extreme fluctuations?: Unknown

Severe fragmentation?: Unknown

#### Habitat

System: Terrestrial

Habitat specialist: Unknown

Habitat (narrative): Since the type locality is unspecified, the preferred habitat for this species cannot be inferred.

Trend in extent, area or quality?: Unknown

##### Habitat

Habitat importance: Major Importance

Habitats: 18. Unknown

#### Habitat

Habitat importance: Major Importance

Habitats: 18. Unknown

#### Ecology

Size: 40 - 50 mm

Generation length (yr): 4

Dependency of single sp?: Unknown

Ecology and traits (narrative): Spiders of the family Theraphosidae are free-living, ground-dwelling spiders. They build a burrow lined with silk or hide in a retreat under a rock (Jocqué and Dippenaar-Schoeman 2006). Theraphosid males do not moult when they reach maturity and they also have a shorter lifespan compared to females (Costa and Pérez-Miles 2002).

#### Threats

Justification for threats: Collection of individuals from the wild to feed the pet market may be a threat to this species.

##### Threats

Threat type: Ongoing

Threats: 5.1.1. Biological resource use - Hunting & trapping terrestrial animals - Intentional use (species is the target)

#### Threats

Threat type: Ongoing

Threats: 5.1.1. Biological resource use - Hunting & trapping terrestrial animals - Intentional use (species is the target)

#### Conservation

Justification for conservation actions: It would be essential to raise awareness and communication of the consequences of the pet trade on exotic animals.

##### Conservation actions

Conservation action type: Needed

Conservation actions: 4.3. Education & awareness - Awareness & communications

#### Conservation actions

Conservation action type: Needed

Conservation actions: 4.3. Education & awareness - Awareness & communications

#### Other

##### Use and trade

Use type: International

Use and trade: 13. Pets/display animals, horticulture

##### Ecosystem services

Ecosystem service type: Very important

##### Research needed

Research needed: 1.2. Research - Population size, distribution & trends1.3. Research - Life history & ecology1.5. Research - Threats

Justification for research needed: Basic research is needed to know the current distribution and population size and trends, ecology and traits of the species along with possible threats besides the possible consequences of pet trade.

#### Use and trade

Use type: International

Use and trade: 13. Pets/display animals, horticulture

#### Ecosystem services

Ecosystem service type: Very important

#### Research needed

Research needed: 1.2. Research - Population size, distribution & trends1.3. Research - Life history & ecology1.5. Research - Threats

Justification for research needed: Basic research is needed to know the current distribution and population size and trends, ecology and traits of the species along with possible threats besides the possible consequences of pet trade.

#### Viability analysis

### Eupalaestrus larae

#### Species information

Scientific name: Eupalaestrus larae

Species authority: Ferretti & Barneche, 2012

Kingdom: Animalia

Phylum: Arthropoda

Class: Arachnida

Order: Araneae

Family: Theraphosidae

Region for assessment: Global

#### Geographic range

Biogeographic realm: Neotropical

Countries: Argentina

Map of records (Google Earth): Suppl. material 16

Basis of EOO and AOO: Unknown

Basis (narrative): Only three records (Ferretti and Barneche 2012) make it impossible to assess the true distribution of the species.

Min Elevation/Depth (m): 90

Max Elevation/Depth (m): 120

Range description: This species is known only from Chaco province, Argentina, found and recorded in 2012 (Ferretti and Barneche 2012).

#### New occurrences

#### Extent of occurrence

EOO (km2): Unknown

Trend: Unknown

Justification for trend: *E. larae* is capable of inhabiting even modified areas with human disturbance (Ferretti and Barneche 2012) which may suggest the EOO of this species is probably not experiencing any decline. Yet, this is very uncertain.

Causes ceased?: Unknown

Causes understood?: Unknown

Causes reversible?: Unknown

Extreme fluctuations?: Unknown

#### Area of occupancy

Trend: Unknown

Justification for trend: *E. larae* is capable of inhabiting even modified areas with human disturbance (Ferretti and Barneche 2012) which may suggest the AOO of this species is probably not experiencing any decline. Yet, this is very uncertain.

Causes ceased?: Unknown

Causes understood?: Unknown

Causes reversible?: Unknown

Extreme fluctuations?: Unknown

AOO (km2): Unknown

#### Locations

Number of locations: Unknown

Justification for number of locations: 

Trend: Unknown

Extreme fluctuations?: Unknown

#### Population

Number of individuals: Unknown

Trend: Unknown

Causes ceased?: Unknown

Causes understood?: Unknown

Causes reversible?: Unknown

Extreme fluctuations?: Unknown

Population Information (Narrative): No population size estimates exist.

#### Subpopulations

Number of subpopulations: Unknown

Trend: Unknown

Extreme fluctuations?: Unknown

Severe fragmentation?: Unknown

#### Habitat

System: Terrestrial

Habitat specialist: No

Habitat (narrative): Specimens were found in the area between humid and dry Chaco in flat grasslands surrounded by forest. This species was also abundant in golf courses along with other artificial and disturbed habitats (Ferretti and Barneche 2012).

Trend in extent, area or quality?: Stable

Justification for trend: This species tolerance to human disturbance can be advantageous to its survival.

##### Habitat

Habitat importance: Major Importance

Habitats: 4.5. Grassland - Subtropical/Tropical Dry14.4. Artificial/Terrestrial - Rural Gardens

#### Habitat

Habitat importance: Major Importance

Habitats: 4.5. Grassland - Subtropical/Tropical Dry14.4. Artificial/Terrestrial - Rural Gardens

#### Ecology

Size: 45.72 - 64.40 mm

Generation length (yr): 4

Dependency of single sp?: Unknown

Ecology and traits (narrative): Spiders of the family Theraphosidae are free-living, ground-dwelling spiders. They build a burrow lined with silk or hide in a retreat under a rock (Jocqué and Dippenaar-Schoeman 2006). Theraphosid males do not moult when they reach maturity and they also have a shorter lifespan compared to females (Costa and Pérez-Miles 2002). *E. larae* is active at night and come outside their burrow to wait for a prey. Burrows can be deep and temperature inside it can be almost 10ºC cooler compared to the outside temperature. The population density can be high with many specimens within a few square metres (Ferretti and Barneche 2012).

#### Threats

Justification for threats: No known threats.

##### Threats

Threat type: Past

Threats: 12. Other options - Other threat

#### Threats

Threat type: Past

Threats: 12. Other options - Other threat

#### Conservation

##### Conservation actions

Conservation action type: In Place

#### Conservation actions

Conservation action type: In Place

#### Other

##### Use and trade

Use type: International

Use and trade: 18. Unknown

##### Ecosystem services

Ecosystem service type: Very important

##### Research needed

Research needed: 1.2. Research - Population size, distribution & trends1.3. Research - Life history & ecology1.5. Research - Threats

Justification for research needed: Basic research is needed to know the current distribution and population size and trends, ecology and traits of the species, along with possible threats.

#### Use and trade

Use type: International

Use and trade: 18. Unknown

#### Ecosystem services

Ecosystem service type: Very important

#### Research needed

Research needed: 1.2. Research - Population size, distribution & trends1.3. Research - Life history & ecology1.5. Research - Threats

Justification for research needed: Basic research is needed to know the current distribution and population size and trends, ecology and traits of the species, along with possible threats.

#### Viability analysis

### Phormictopus platus

#### Species information

Scientific name: Phormictopus platus

Species authority: Chamberlin, 1917

Kingdom: Animalia

Phylum: Arthropoda

Class: Arachnida

Order: Araneae

Family: Theraphosidae

Region for assessment: Global

#### Geographic range

Biogeographic realm: Nearctic

Countries: United States

Map of records (Google Earth): Suppl. material 17

Basis of EOO and AOO: Unknown

Basis (narrative): Unknown EOO or AOO

Min Elevation/Depth (m): 0

Max Elevation/Depth (m): 0

Range description: This species has been recorded only once from an unconfirmed type locality prior to 1917 (Chamberlin 1917). The description paper sets the type locality as Tortugas in Florida and therefore the type locality here is considered as Dry Tortugas National Park. However, Rudloff (2008) suggested this locality would be more likely Tortuga Island in Hispaniola (Cuba). Hence, the known distribution of this species remains unconfirmed.

#### New occurrences

#### Extent of occurrence

EOO (km2): Unknown

Trend: Unknown

Causes ceased?: Unknown

Causes understood?: Unknown

Causes reversible?: Unknown

Extreme fluctuations?: Unknown

#### Area of occupancy

Trend: Unknown

Causes ceased?: Unknown

Causes understood?: Unknown

Causes reversible?: Unknown

Extreme fluctuations?: Unknown

AOO (km2): Unknown

#### Locations

Number of locations: Unknown

Trend: Unknown

Extreme fluctuations?: Unknown

#### Population

Number of individuals: Unknown

Trend: Unknown

Causes ceased?: Unknown

Causes understood?: Unknown

Causes reversible?: Unknown

Extreme fluctuations?: Unknown

Population Information (Narrative): No population size estimates exist.

#### Subpopulations

Number of subpopulations: Unknown

Trend: Unknown

Extreme fluctuations?: Unknown

Severe fragmentation?: Unknown

#### Habitat

System: Terrestrial

Habitat specialist: Unknown

Habitat (narrative): Since the type locality is unspecified, the preferred habitat by this species cannot be inferred.

Trend in extent, area or quality?: Unknown

##### Habitat

Habitat importance: Major Importance

Habitats: 18. Unknown

#### Habitat

Habitat importance: Major Importance

Habitats: 18. Unknown

#### Ecology

Size: 52 mm

Generation length (yr): 4

Dependency of single sp?: No

Ecology and traits (narrative): Spiders of the family Theraphosidae are free-living, ground-dwelling spiders. They build a burrow lined with silk or hide in a retreat under a rock (Jocqué and Dippenaar-Schoeman 2006). Theraphosid males do not moult when they reach maturity and they also have a shorter lifespan compared to females (Costa and Pérez-Miles 2002).

#### Threats

Justification for threats: Unknown threats.

##### Threats

Threat type: Past

Threats: 12. Other options - Other threat

#### Threats

Threat type: Past

Threats: 12. Other options - Other threat

#### Conservation

##### Conservation actions

Conservation action type: In Place

#### Conservation actions

Conservation action type: In Place

#### Other

##### Use and trade

Use type: International

##### Ecosystem services

Ecosystem service type: Very important

##### Research needed

Research needed: 1.2. Research - Population size, distribution & trends1.3. Research - Life history & ecology1.5. Research - Threats

Justification for research needed: Basic research is needed to know the current distribution and population size and trends, ecology and traits of the species, along with possible threats.

#### Use and trade

Use type: International

#### Ecosystem services

Ecosystem service type: Very important

#### Research needed

Research needed: 1.2. Research - Population size, distribution & trends1.3. Research - Life history & ecology1.5. Research - Threats

Justification for research needed: Basic research is needed to know the current distribution and population size and trends, ecology and traits of the species, along with possible threats.

#### Viability analysis

### Plesiopelma myodes

#### Species information

Scientific name: Plesiopelma myodes

Species authority: Pocock, 1901

Kingdom: Animalia

Phylum: Arthropoda

Class: Arachnida

Order: Araneae

Family: Theraphosidae

Region for assessment: Global

#### Geographic range

Biogeographic realm: Neotropical

Countries: UruguayBrazil

Map of records (Google Earth): Suppl. material 18

Basis of EOO and AOO: Unknown

Basis (narrative): Unknown EOO or AOO.

Min Elevation/Depth (m): 90

Max Elevation/Depth (m): 120

Range description: Known only from two localities, from Soriano in Uruguay, recorded prior to 1901 (Pocock 1901) and from Rio Grande do Sul in Brazil, recorded prior to 1923 (Mello-Leitão 1923).

#### New occurrences

#### Extent of occurrence

EOO (km2): Unknown

Trend: Unknown

Causes ceased?: Unknown

Causes understood?: Unknown

Causes reversible?: Unknown

Extreme fluctuations?: Unknown

#### Area of occupancy

Trend: Unknown

Causes ceased?: Unknown

Causes understood?: Unknown

Causes reversible?: Unknown

Extreme fluctuations?: Unknown

AOO (km2): Unknown

#### Locations

Number of locations: Unknown

Trend: Unknown

Extreme fluctuations?: Unknown

#### Population

Number of individuals: Unknown

Trend: Unknown

Causes ceased?: Unknown

Causes understood?: Unknown

Causes reversible?: Unknown

Extreme fluctuations?: Unknown

Population Information (Narrative): Population size and trend are unknown.

#### Subpopulations

Number of subpopulations: Unknown

Trend: Unknown

Extreme fluctuations?: Unknown

Severe fragmentation?: Unknown

#### Habitat

System: Terrestrial

Habitat specialist: Unknown

Habitat (narrative): Unknown preferred habitat.

Trend in extent, area or quality?: Unknown

##### Habitat

Habitat importance: Major Importance

Habitats: 18. Unknown

#### Habitat

Habitat importance: Major Importance

Habitats: 18. Unknown

#### Ecology

Size: 26 - 35 mm

Generation length (yr): 4

Dependency of single sp?: Unknown

Ecology and traits (narrative): Spiders of the family Theraphosidae are free-living, ground-dwelling spiders. They build a burrow lined with silk or hide in a retreat under a rock (Jocqué and Dippenaar-Schoeman 2006). Theraphosid males do not moult when they reach maturity and they also have a shorter lifespan compared to females. *Plesiopelma* species line their burrows with silk. One species from the same genus, *Plesiopelma longisternale*, was captured by pitfall traps in Uruguay (particularly in Sierra de las Animas, Maldonado and Quebrada de los Cuervos, Treinta y Tres) (Costa and Pérez-Miles 2002). Ferretti et al. (2012) observed that both females and juveniles of *Plesiopelma longisternale* were absent during a same seasonal period and were found from pitfall traps. Males have been collected at least from April to December. Females were found carrying egg-sacs during December and January in the field and the sacs were reported to contain over 100 eggs. *P. longisternale* females were reported to live over 4 years in the laboratory and moulted every 1.5 years, usually in spring (Costa and Pérez-Miles 2002).

#### Threats

Justification for threats: Unknown threats.

##### Threats

Threat type: Past

Threats: 12. Other options - Other threat

#### Threats

Threat type: Past

Threats: 12. Other options - Other threat

#### Conservation

##### Conservation actions

#### Conservation actions

#### Other

##### Use and trade

Use type: International

##### Ecosystem services

Ecosystem service type: Very important

##### Research needed

Research needed: 1.1. Research - Taxonomy1.2. Research - Population size, distribution & trends1.3. Research - Life history & ecology1.5. Research - Threats

Justification for research needed: Basic research is needed to know the current distribution and population size and trends, ecology and traits of the species, along with possible threats. Since the last publication is over 90 years old, a taxonomic review would be needed to confirm the species status.

#### Use and trade

Use type: International

#### Ecosystem services

Ecosystem service type: Very important

#### Research needed

Research needed: 1.1. Research - Taxonomy1.2. Research - Population size, distribution & trends1.3. Research - Life history & ecology1.5. Research - Threats

Justification for research needed: Basic research is needed to know the current distribution and population size and trends, ecology and traits of the species, along with possible threats. Since the last publication is over 90 years old, a taxonomic review would be needed to confirm the species status.

#### Viability analysis

### Poecilotheria subfusca

#### Species information

Scientific name: Poecilotheria subfusca

Species authority: Pocock, 1895

Kingdom: Animalia

Phylum: Arthropoda

Class: Arachnida

Order: Araneae

Family: Theraphosidae

Region for assessment: Global

#### Geographic range

Biogeographic realm: Indomalayan

Countries: Sri Lanka

Map of records (Google Earth): Suppl. material 19

Basis of EOO and AOO: Species Distribution Model

Basis (narrative): Given the relatively high number of records (Pocock 1895, Samarawckrama et al. 2005, Benjamin et al. 2012, Gabriel 2013), it was possible to perform species distribution modelling (see methods for details).

Min Elevation/Depth (m): 170

Max Elevation/Depth (m): 2410

Range description: This species is endemic to Sri Lanka and restricted to its central parts, last recorded in the wild in the 1990s but many pet records are from 2000s (Pocock 1895, Samarawckrama et al. 2005, Benjamin et al. 2012, Gabriel 2013).

#### New occurrences

#### Extent of occurrence

EOO (km2): 6152

Trend: Decline (inferred)

Justification for trend: Large and expanding human settlements surround the existing habitat of the species.

Causes ceased?: No

Causes understood?: Yes

Causes reversible?: No

Extreme fluctuations?: No

#### Area of occupancy

Trend: Decline (inferred)

Justification for trend: Large and expanding human settlements surround the existing habitat of the species.

Causes ceased?: No

Causes understood?: Yes

Causes reversible?: No

Extreme fluctuations?: No

AOO (km2): 5208

#### Locations

Number of locations: Unknown

Trend: Decline (inferred)

Justification for trend: Based on predicted forest loss in the area (Global Forest Watch 2014).

Extreme fluctuations?: Unknown

#### Population

Number of individuals: Unknown

Trend: Decline (inferred)

Justification for trend: Inferred from the loss of AOO and habitat quality. Possible decrease of population size due to the pet trade is also a strong possibility.

Basis for decline: (c) a decline in area of occupancy, extent of occurrence and/or quality of habitat(d) actual or potential levels of exploitation

Causes ceased?: No

Causes understood?: Yes

Causes reversible?: No

Extreme fluctuations?: Unknown

Population Information (Narrative): Population size is unknown but inferred to be declining due to habitat loss and capture from nature due to pet trade.

#### Subpopulations

Number of subpopulations: Unknown

Trend: Decline (inferred)

Justification for trend: Large and expanding human settlements surround the existing habitat of the species.

Extreme fluctuations?: No

Severe fragmentation?: Unknown

#### Habitat

System: Terrestrial

Habitat specialist: Yes

Habitat (narrative): Species in this genus prefer very specific microhabitats, mainly tree holes and on the bark of trees and are found in remnant forests surrounded by extensive human settlements in Sri Lanka.

Trend in extent, area or quality?: Decline (observed)

Justification for trend: Large and expanding human settlements surround the existing habitat of the species (Global Forest Watch 2014).

##### Habitat

Habitat importance: Major Importance

Habitats: 1.9. Forest - Subtropical/Tropical Moist Montane

#### Habitat

Habitat importance: Major Importance

Habitats: 1.9. Forest - Subtropical/Tropical Moist Montane

#### Ecology

Size: 60 mm

Generation length (yr): 6

Dependency of single sp?: No

Ecology and traits (narrative): Species of *Poecilotheria* are tree-dwellers, where they spin small webs in the bifurcations of branches. Given their large size, they might feed on small vertebrates besides the regular large invertebrate diet.

#### Threats

Justification for threats: Habitat transformation and the pet trade form the main threats to this species.

##### Threats

Threat type: Ongoing

Threats: 1.1. Residential & commercial development - Housing & urban areas5.1.1. Biological resource use - Hunting & trapping terrestrial animals - Intentional use (species is the target)

#### Threats

Threat type: Ongoing

Threats: 1.1. Residential & commercial development - Housing & urban areas5.1.1. Biological resource use - Hunting & trapping terrestrial animals - Intentional use (species is the target)

#### Conservation

Justification for conservation actions: At least part of the species estimated range is within protected areas, namely Knuckles and Pedro Forest/Pidurutalagala Conservation Forest and Agra bopats P.R State Forest in Sri Lanka (United Nations Environment World Conservation Monitoring Centre 2017). Large and expanding human settlements cause a threat to this species and they are in need of additional protection. More awareness on the side effects of the pet trade, namely increasing pressure over wild populations, needs to be made.

##### Conservation actions

Conservation action type: In Place

Conservation actions: 1.1. Land/water protection - Site/area protection

##### Conservation actions

Conservation action type: Needed

Conservation actions: 1.2. Land/water protection - Resource & habitat protection4.3. Education & awareness - Awareness & communications

#### Conservation actions

Conservation action type: In Place

Conservation actions: 1.1. Land/water protection - Site/area protection

#### Conservation actions

Conservation action type: Needed

Conservation actions: 1.2. Land/water protection - Resource & habitat protection4.3. Education & awareness - Awareness & communications

#### Other

Justification for use and trade: Traded at the international level.

##### Use and trade

Use type: International

Use and trade: 13. Pets/display animals, horticulture

##### Ecosystem services

Ecosystem service type: Very important

##### Research needed

Research needed: 2.1. Conservation Planning - Species Action/Recovery Plan2.2. Conservation Planning - Area-based Management Plan2.3. Conservation Planning - Harvest & Trade Management Plan3.1. Monitoring - Population trends3.4. Monitoring - Habitat trends

Justification for research needed: Monitoring is needed to confirm the inferred population and habitat trends. Also conservation planning would be essential to the survival of this species since it is endemic to a small part of Sri Lanka and with decreasing population numbers.

#### Use and trade

Use type: International

Use and trade: 13. Pets/display animals, horticulture

#### Ecosystem services

Ecosystem service type: Very important

#### Research needed

Research needed: 2.1. Conservation Planning - Species Action/Recovery Plan2.2. Conservation Planning - Area-based Management Plan2.3. Conservation Planning - Harvest & Trade Management Plan3.1. Monitoring - Population trends3.4. Monitoring - Habitat trends

Justification for research needed: Monitoring is needed to confirm the inferred population and habitat trends. Also conservation planning would be essential to the survival of this species since it is endemic to a small part of Sri Lanka and with decreasing population numbers.

#### Viability analysis

### Dipoena appalachia

#### Species information

Scientific name: Dipoena appalachia

Species authority: Levi, 1953

Kingdom: Animalia

Phylum: Arthropoda

Class: Arachnida

Order: Araneae

Family: Theridiidae

Region for assessment: Global

#### Geographic range

Biogeographic realm: Nearctic

Countries: CanadaUnited States

Map of records (Google Earth): Suppl. material 20

Basis of EOO and AOO: Species Distribution Model

Basis (narrative): Given the relatively high number of records (Levi 1953, Paquin et al. 2008), it was possible to perform species distribution modelling (see methods for details).

Min Elevation/Depth (m): 0

Max Elevation/Depth (m): 690

Range description: This species was found in many localities from the south-eastern United States (Levi 1953) to Quebec, Canada (Paquin et al. 2008). It appears the species is widespread and occurs in a number of different climatic regions.

#### New occurrences

#### Extent of occurrence

EOO (km2): 2729841

Trend: Stable

Justification for trend: Although no monitoring was conducted, given the wide range and no known threats, we infer the trend to be stable.

Causes ceased?: Yes

Causes understood?: Yes

Causes reversible?: Yes

Extreme fluctuations?: No

#### Area of occupancy

Trend: Stable

Justification for trend: Although no monitoring was conducted, given the wide range and no known threats, we infer the trend to be stable.

Causes ceased?: Yes

Causes understood?: Yes

Causes reversible?: Yes

Extreme fluctuations?: No

AOO (km2): 1453664

#### Locations

Number of locations: Not applicable

Justification for number of locations: No known threats to the species.

Trend: Stable

Extreme fluctuations?: No

#### Population

Number of individuals: Unknown

Trend: Stable

Justification for trend: Although no monitoring was conducted, given the wide range and no known threats, we infer the trend to be stable.

Causes ceased?: Yes

Causes understood?: Yes

Causes reversible?: Yes

Extreme fluctuations?: No

Population Information (Narrative): No population size estimates exist.

#### Subpopulations

Number of subpopulations: Unknown

Trend: Stable

Justification for trend: Although no monitoring was conducted, given the wide range and no known threats, we infer the trend to be stable.

Extreme fluctuations?: No

Severe fragmentation?: No

#### Habitat

System: Terrestrial

Habitat specialist: Unknown

Habitat (narrative): Paquin et al. (2008) report this species from mixed forest in Quebec, Canada. Individuals were collected in pan traps and from beating. Several older records from the southeast lack habitat information, except one record from Maryland, USA from "old bird nest" (Levi 1953).

Trend in extent, area or quality?: Stable

Justification for trend: Given the wide range, it is doubtful that the species occupies a specific habitat, but the exact habitat requirements are unknown.

##### Habitat

Habitat importance: Major Importance

Habitats: 1.4. Forest - Temperate

#### Habitat

Habitat importance: Major Importance

Habitats: 1.4. Forest - Temperate

#### Ecology

Size: 1.1 - 1.6 mm

Generation length (yr): 1

Dependency of single sp?: Unknown

Ecology and traits (narrative): Ecology of this particular species is largely unknown. Contrary to most theridiids, Dipoena do not build webs and feed almost exclusively on ants at ground level, on low bushes or on the bark of trees.

#### Threats

Justification for threats: No known threats to the species

##### Threats

Threat type: Past

Threats: 12. Other options - Other threat

#### Threats

Threat type: Past

Threats: 12. Other options - Other threat

#### Conservation

Justification for conservation actions: At least part of the species range is inside protected areas, namely Parc National de la Yamaska in Quebec, Canada (Paquin et al. 2008) and several other conservation areas and National Parks within the USA (United Nations Environment World Conservation Monitoring Centre 2017).

##### Conservation actions

Conservation action type: In Place

Conservation actions: 1.1. Land/water protection - Site/area protection1.2. Land/water protection - Resource & habitat protection

#### Conservation actions

Conservation action type: In Place

Conservation actions: 1.1. Land/water protection - Site/area protection1.2. Land/water protection - Resource & habitat protection

#### Other

##### Use and trade

Use type: International

##### Ecosystem services

Ecosystem service type: Very important

##### Research needed

Research needed: 3.1. Monitoring - Population trends3.4. Monitoring - Habitat trends

Justification for research needed: Monitoring is needed to confirm current population and habitat trends.

#### Use and trade

Use type: International

#### Ecosystem services

Ecosystem service type: Very important

#### Research needed

Research needed: 3.1. Monitoring - Population trends3.4. Monitoring - Habitat trends

Justification for research needed: Monitoring is needed to confirm current population and habitat trends.

#### Viability analysis

### Lasaeola convexa

#### Species information

Scientific name: Lasaeola convexa

Species authority: (Blackwall, 1870)

Kingdom: Animalia

Phylum: Arthropoda

Class: Arachnida

Order: Araneae

Family: Theridiidae

Taxonomic notes: *L. convexa* has been transferred between the genera *Dipoena* and *Lasaeola* for several times (e.g. Wunderlich 2011, Le Peru 2011, IJland et al. 2012) and therefore further taxonomic clarification would be needed.

Region for assessment: Global

#### Geographic range

Biogeographic realm: Palearctic

Countries: CyprusIsraelTurkeyMacedonia, the former Yugoslav Republic ofMaltaRomaniaSan MarinoAlbaniaLiechtensteinLuxembourgBelgiumBosnia and HerzegovinaBulgariaCroatiaCzech RepublicGermanySloveniaSwitzerlandAustriaHungaryMontenegroWestern SaharaAlgeriaTunisiaMoroccoMauritaniaPortugalFranceGreeceSpainItalySerbiaUkraine

Map of records (Google Earth): Suppl. material 21

Basis of EOO and AOO: Species Distribution Model

Basis (narrative): Given the relatively high number of records (Blackwall 1870, Simon 1881, Chyzer and Kulczyński 1894, Brignoli 1967, Brignoli 1976, Levy and Amitai 1981, Vanuytven et al. 1994, Naumova 2009, Wunderlich 2011, Elverici 2012, Helsdingen and Ijland 2015, Dimassi et al. 2016), it was possible to perform species distribution modelling (see methods for details).

Min Elevation/Depth (m): 0

Max Elevation/Depth (m): 2090

Range description: This species should be widely distributed across the Mediterranean and further north (Blackwall 1870, Simon 1881, Chyzer and Kulczyński 1894, Brignoli 1967, Brignoli 1976, Levy and Amitai 1981, Vanuytven et al. 1994, Naumova 2009, Wunderlich 2011, Elverici 2012, Helsdingen and Ijland 2015, Dimassi et al. 2016).

#### New occurrences

#### Extent of occurrence

EOO (km2): 8929003

Trend: Stable

Justification for trend: As it is a widespread species with no specific habitat requirements or known threats, we assume the trend to be stable.

Causes ceased?: Yes

Causes understood?: Yes

Causes reversible?: Yes

Extreme fluctuations?: No

#### Area of occupancy

Trend: Stable

Justification for trend: As it is a widespread species with no specific habitat requirements or known threats, we assume the trend to be stable.

Causes ceased?: Yes

Causes understood?: Yes

Causes reversible?: Yes

Extreme fluctuations?: No

AOO (km2): 3438164

#### Locations

Number of locations: Not applicable

Justification for number of locations: No known threats to the species.

Trend: Stable

Extreme fluctuations?: No

#### Population

Number of individuals: Unknown

Trend: Stable

Justification for trend: As it is a widespread species with no specific habitat requirements or known threats, we assume the trend to be stable.

Causes ceased?: Unknown

Causes understood?: Yes

Causes reversible?: Yes

Extreme fluctuations?: No

Population Information (Narrative): No population size estimates exist.

#### Subpopulations

Number of subpopulations: Unknown

Trend: Stable

Justification for trend: As it is a widespread species with no specific habitat requirements or known threats, we assume the trend to be stable.

Extreme fluctuations?: No

Severe fragmentation?: No

#### Habitat

System: Terrestrial

Habitat specialist: No

Habitat (narrative): *L. convexa* prefers dry habitats and lives on bushes and under stones (Le Peru 2011); specimens were found from shrublands, rocky and grazed areas with Juniper trees (*Juniperus oxycedrus* and *J. phoenica*) (IJland et al. 2012). It also occurs in coniferous forests and has been collected from slopes with stony debris (Helsdingen and Ijland 2015).

Trend in extent, area or quality?: Stable

Justification for trend: Dry and xerix habitats are in fact increasing and therefore the habitat trend for this species can be inferred to be at least stable.

##### Habitat

Habitat importance: Major Importance

Habitats: 1.4. Forest - Temperate3.4. Shrubland - Temperate3.8. Shrubland - Mediterranean-type Shrubby Vegetation

#### Habitat

Habitat importance: Major Importance

Habitats: 1.4. Forest - Temperate3.4. Shrubland - Temperate3.8. Shrubland - Mediterranean-type Shrubby Vegetation

#### Ecology

Size: 1.7 - 4 mm

Generation length (yr): 1

Dependency of single sp?: No

Ecology and traits (narrative): Ecology of this particular species is largely unknown. Contrary to most theridiids, *Lasaeola* do not build webs and feed almost exclusively on ants at ground level, on low bushes or on the bark of trees. Females occur from January to June and also in November, while males are seen in spring and in October (Le Peru 2011).

#### Threats

Justification for threats: No known threats to the species.

##### Threats

Threat type: Past

Threats: 12. Other options - Other threat

#### Threats

Threat type: Past

Threats: 12. Other options - Other threat

#### Conservation

Justification for conservation actions: There are several protected areas inside the range of this species (United Nations Environment World Conservation Monitoring Centre 2017).

##### Conservation actions

Conservation action type: In Place

Conservation actions: 1.1. Land/water protection - Site/area protection1.2. Land/water protection - Resource & habitat protection

#### Conservation actions

Conservation action type: In Place

Conservation actions: 1.1. Land/water protection - Site/area protection1.2. Land/water protection - Resource & habitat protection

#### Other

##### Use and trade

Use type: International

##### Ecosystem services

Ecosystem service type: Very important

##### Research needed

Research needed: 3.1. Monitoring - Population trends3.4. Monitoring - Habitat trends

Justification for research needed: Monitoring is needed to confirm current population and habitat trends.

#### Use and trade

Use type: International

#### Ecosystem services

Ecosystem service type: Very important

#### Research needed

Research needed: 3.1. Monitoring - Population trends3.4. Monitoring - Habitat trends

Justification for research needed: Monitoring is needed to confirm current population and habitat trends.

#### Viability analysis

### Sesato setosa

#### Species information

Scientific name: Sesato setosa

Species authority: Saaristo, 2006

Kingdom: Animalia

Phylum: Arthropoda

Class: Arachnida

Order: Araneae

Family: Theridiidae

Region for assessment: Global

#### Geographic range

Biogeographic realm: Afrotropical

Countries: Seychelles

Map of records (Google Earth): Suppl. material 22

Basis of EOO and AOO: Species Distribution Model

Basis (narrative): Given the relatively high number of records (Saaristo 2006), it was possible to perform species distribution modelling (see methods for details).

Min Elevation/Depth (m): 0

Max Elevation/Depth (m): 450

Range description: This species is endemic to the Seychelles island of Silhouette, all records are from 1990s (Saaristo 2006).

#### New occurrences

#### Extent of occurrence

EOO (km2): 32

Trend: Unknown

Justification for trend: The habitat has been deteriorating due to the effects of invasive plant species, yet we do not know if this affects the species.

Causes ceased?: Unknown

Causes understood?: Unknown

Causes reversible?: Unknown

Extreme fluctuations?: No

#### Area of occupancy

Trend: Unknown

Justification for trend: The habitat has been deteriorating due to the effects of invasive plant species, yet we do not know if this affects the species.

Causes ceased?: Unknown

Causes understood?: Unknown

Causes reversible?: Unknown

Extreme fluctuations?: No

AOO (km2): 32

#### Locations

Number of locations: Unknown

Trend: Unknown

Extreme fluctuations?: Unknown

#### Population

Number of individuals: Unknown

Trend: Unknown

Causes ceased?: Unknown

Causes understood?: Unknown

Causes reversible?: Unknown

Extreme fluctuations?: No

Population Information (Narrative): No estimates exist.

#### Subpopulations

Number of subpopulations: Unknown

Trend: Unknown

Extreme fluctuations?: Unknown

Severe fragmentation?: Unknown

#### Habitat

System: Terrestrial

Habitat specialist: Yes

Habitat (narrative): This species was found only in *Pisonia* forest and spins its webs in vegetation (Saaristo 2006).

Trend in extent, area or quality?: Unknown

Justification for trend: Although the habitat has been deteriorating due to the effects of invasive plant species, *Sesato setosa* appears to dwell in areas around the local village, hence it is unknown if the species is being affected.

##### Habitat

Habitat importance: Major Importance

Habitats: 1.6. Forest - Subtropical/Tropical Moist Lowland

#### Habitat

Habitat importance: Major Importance

Habitats: 1.6. Forest - Subtropical/Tropical Moist Lowland

#### Ecology

Size: 1.65 mm

Generation length (yr): 1

Dependency of single sp?: Unknown

Ecology and traits (narrative): This species spins its web in the vegetation (Saaristo 2006). Otherwise, the ecology of this particular species is largely unknown. Theridiids in general build space webs which are irregular in shape; threads are often configured in different directions (Jocqué and Dippenaar-Schoeman 2006). These threads tend to break easily when capturing prey. These glue-bearing threads make it difficult for prey to flee and easy for a spider to capture them.

#### Threats

Justification for threats: This species habitat is threatened due to the effects of invasive plants, especially *Cinnamomum verum*. Yet, we do not know if it affects the spider.

##### Threats

Threat type: Ongoing

Threats: 8.1.2. Invasive and other problematic species, genes & diseases - Invasive non-native/alien species/diseases - Named species

#### Threats

Threat type: Ongoing

Threats: 8.1.2. Invasive and other problematic species, genes & diseases - Invasive non-native/alien species/diseases - Named species

#### Conservation

Justification for conservation actions: This species is found in the Silhouette National Park, yet the park is not currently managed. Invasive species were managed on Silhouette until 2010 but any actions have been abandoned since. If the spider is in any way affected by invasive plant species, it may be essential to its survival to take consider invasive species management as a possible action.

##### Conservation actions

Conservation action type: In Place

Conservation actions: 1.1. Land/water protection - Site/area protection

##### Conservation actions

Conservation action type: Needed

Conservation actions: 2.1. Land/water management - Site/area management2.2. Land/water management - Invasive/problematic species control

#### Conservation actions

Conservation action type: In Place

Conservation actions: 1.1. Land/water protection - Site/area protection

#### Conservation actions

Conservation action type: Needed

Conservation actions: 2.1. Land/water management - Site/area management2.2. Land/water management - Invasive/problematic species control

#### Other

##### Use and trade

Use type: International

##### Ecosystem services

Ecosystem service type: Very important

##### Research needed

Research needed: 1.5. Research - Threats2.2. Conservation Planning - Area-based Management Plan3.1. Monitoring - Population trends3.4. Monitoring - Habitat trends

Justification for research needed: This species is present in the Silhouette National Park but this is not managed. To know the current population trends implies evaluating the true impact of the invasive plant species on the spider. Also, monitoring of habitat and population trends would be needed.

#### Use and trade

Use type: International

#### Ecosystem services

Ecosystem service type: Very important

#### Research needed

Research needed: 1.5. Research - Threats2.2. Conservation Planning - Area-based Management Plan3.1. Monitoring - Population trends3.4. Monitoring - Habitat trends

Justification for research needed: This species is present in the Silhouette National Park but this is not managed. To know the current population trends implies evaluating the true impact of the invasive plant species on the spider. Also, monitoring of habitat and population trends would be needed.

#### Viability analysis

### Steatoda xerophila

#### Species information

Scientific name: Steatoda xerophila

Species authority: Levy & Amitai, 1982

Kingdom: Animalia

Phylum: Arthropoda

Class: Arachnida

Order: Araneae

Family: Theridiidae

Region for assessment: Global

#### Geographic range

Biogeographic realm: Palearctic

Countries: Israel

Map of records (Google Earth): Suppl. material 23

Basis of EOO and AOO: Unknown

Basis (narrative): Very few records in Israel (Levy and Amitai 1982, Levy 1998) do not allow knowing the true range of the species.

Min Elevation/Depth (m): 380

Max Elevation/Depth (m): 660

Range description: This species has been recorded for the Negev, a desertic and semidesertic region of southern Israel, recorded in 1973 (Levy and Amitai 1982).

#### New occurrences

#### Extent of occurrence

EOO (km2): Unknown

Trend: Unknown

Causes ceased?: Unknown

Causes understood?: Unknown

Causes reversible?: Unknown

Extreme fluctuations?: Unknown

#### Area of occupancy

Trend: Unknown

Causes ceased?: Unknown

Causes understood?: Unknown

Causes reversible?: Unknown

Extreme fluctuations?: Unknown

AOO (km2): Unknown

#### Locations

Number of locations: Unknown

Trend: Unknown

Extreme fluctuations?: Unknown

#### Population

Number of individuals: Unknown

Trend: Unknown

Causes ceased?: Unknown

Causes understood?: Unknown

Causes reversible?: Unknown

Extreme fluctuations?: Unknown

Population Information (Narrative): No population size estimates exist.

#### Subpopulations

Number of subpopulations: Unknown

Trend: Unknown

Extreme fluctuations?: Unknown

Severe fragmentation?: Unknown

#### Habitat

System: Terrestrial

Habitat specialist: Yes

Habitat (narrative): This species is found in arid desertic and semidesertic areas of the Negev, southern Israel. (Levy and Amitai 1982).

Trend in extent, area or quality?: Stable

Justification for trend: We do not know of any threats to the habitat.

##### Habitat

Habitat importance: Major Importance

Habitats: 8.1. Desert - Hot

#### Habitat

Habitat importance: Major Importance

Habitats: 8.1. Desert - Hot

#### Ecology

Size: 6.3 - 6.6 mm

Generation length (yr): 1

Dependency of single sp?: Unknown

Ecology and traits (narrative): Ecology of this particular species is largely unknown. Theridiids in general build space webs which are irregular in shape; threads are often configured in different directions (Jocqué and Dippenaar-Schoeman 2006). These threads tend to break easily when capturing prey. These glue-bearing threads make it difficult for prey to flee and easy for a spider to capture them.

#### Threats

Justification for threats: Unknown threats.

##### Threats

Threat type: Past

Threats: 12. Other options - Other threat

#### Threats

Threat type: Past

Threats: 12. Other options - Other threat

#### Conservation

##### Conservation actions

#### Conservation actions

#### Other

##### Use and trade

Use type: International

##### Ecosystem services

Ecosystem service type: Very important

##### Research needed

Research needed: 1.2. Research - Population size, distribution & trends1.3. Research - Life history & ecology1.5. Research - Threats

Justification for research needed: Basic research is needed to know the current distribution and population size and trends, ecology and traits of the species along with possible threats.

#### Use and trade

Use type: International

#### Ecosystem services

Ecosystem service type: Very important

#### Research needed

Research needed: 1.2. Research - Population size, distribution & trends1.3. Research - Life history & ecology1.5. Research - Threats

Justification for research needed: Basic research is needed to know the current distribution and population size and trends, ecology and traits of the species along with possible threats.

#### Viability analysis

### Theridion miserum

#### Species information

Scientific name: Theridion miserum

Species authority: Thorell, 1898

Kingdom: Animalia

Phylum: Arthropoda

Class: Arachnida

Order: Araneae

Family: Theridiidae

Region for assessment: Global

#### Geographic range

Biogeographic realm: Indomalayan

Countries: Myanmar

Map of records (Google Earth): Suppl. material 24

Basis of EOO and AOO: Unknown

Basis (narrative): Unknown EOO or AOO.

Min Elevation/Depth (m): 110

Max Elevation/Depth (m): 110

Range description: This species is known only from the type locality in Myanmar, prior to 1898 (Thorell 1898).

#### New occurrences

#### Extent of occurrence

EOO (km2): Unknown

Trend: Unknown

Causes ceased?: Unknown

Causes understood?: Unknown

Causes reversible?: Unknown

Extreme fluctuations?: Unknown

#### Area of occupancy

Trend: Unknown

Causes ceased?: Unknown

Causes understood?: Unknown

Causes reversible?: Unknown

Extreme fluctuations?: Unknown

AOO (km2): Unknown

#### Locations

Number of locations: Unknown

Trend: Unknown

Extreme fluctuations?: Unknown

#### Population

Number of individuals: Unknown

Trend: Unknown

Causes ceased?: Unknown

Causes understood?: Unknown

Causes reversible?: Unknown

Extreme fluctuations?: Unknown

Population Information (Narrative): No population size estimates exist.

#### Subpopulations

Number of subpopulations: Unknown

Trend: Unknown

Extreme fluctuations?: Unknown

Severe fragmentation?: Unknown

#### Habitat

System: Terrestrial

Habitat specialist: Unknown

Habitat (narrative): Myanmar belongs to the ecoregion of tropical and subtropical moist broadleaf forests (Olson et al. 2001). Otherwise the habitat requirements of this particular species remain unknown.

Trend in extent, area or quality?: Unknown

##### Habitat

Habitat importance: Major Importance

Habitats: 18. Unknown

#### Habitat

Habitat importance: Major Importance

Habitats: 18. Unknown

#### Ecology

Size: >2 mm

Generation length (yr): 1

Dependency of single sp?: Unknown

Ecology and traits (narrative): Ecology of this particular species is largely unknown. Theridiids in general build space webs which are irregular in shape; threads are often configured in different directions (Jocqué and Dippenaar-Schoeman 2006). These threads tend to break easily when capturing prey. These glue-bearing threads make it difficult for prey to flee and easy for a spider to capture them.

#### Threats

Justification for threats: Unknown threats.

##### Threats

Threat type: Past

Threats: 12. Other options - Other threat

#### Threats

Threat type: Past

Threats: 12. Other options - Other threat

#### Conservation

##### Conservation actions

#### Conservation actions

#### Other

##### Use and trade

Use type: International

##### Ecosystem services

Ecosystem service type: Very important

##### Research needed

Research needed: 1.2. Research - Population size, distribution & trends1.3. Research - Life history & ecology1.5. Research - Threats

Justification for research needed: Basic research is needed to know the current distribution and population size and trends, ecology and traits of the species, along with possible threats.

#### Use and trade

Use type: International

#### Ecosystem services

Ecosystem service type: Very important

#### Research needed

Research needed: 1.2. Research - Population size, distribution & trends1.3. Research - Life history & ecology1.5. Research - Threats

Justification for research needed: Basic research is needed to know the current distribution and population size and trends, ecology and traits of the species, along with possible threats.

#### Viability analysis

### Theridion xianfengense

#### Species information

Scientific name: Theridion xianfengense

Species authority: Zhu & Song, 1992

Kingdom: Animalia

Phylum: Arthropoda

Class: Arachnida

Order: Araneae

Family: Theridiidae

Region for assessment: Global

#### Geographic range

Biogeographic realm: IndomalayanPalearctic

Countries: MyanmarChinaTaiwan, Province of China

Map of records (Google Earth): Suppl. material 25

Basis of EOO and AOO: Species Distribution Model

Basis (narrative): Given the relatively high number of records (Zhu and Song 1992, Song and Li 1997, Song et al. 1999, Yoshida et al. 2000, Yin et al. 2012), it was possible to perform species distribution modelling (see methods for details).

Min Elevation/Depth (m): 0

Max Elevation/Depth (m): 1830

Range description: Recorded from several sites in China between the 1980s and 1990s (Zhu and Song 1992, Song and Li 1997, Song et al. 1999, Yoshida et al. 2000, Yin et al. 2012). The last known record is from Orchid Island, Taiwan (Yoshida et al. 2000). This species is predicted to also occur in Myanmar.

#### New occurrences

#### Extent of occurrence

EOO (km2): 1719562

Trend: Stable

Justification for trend: As it is a widespread species with no specific habitat requirements or known threats, we assume the trend to be stable.

Causes ceased?: Yes

Causes understood?: Yes

Causes reversible?: Yes

Extreme fluctuations?: No

#### Area of occupancy

Trend: Stable

Justification for trend: As it is a widespread species with no specific habitat requirements or known threats, we assume the trend to be stable.

Causes ceased?: Yes

Causes understood?: Yes

Causes reversible?: Yes

Extreme fluctuations?: No

AOO (km2): 515972

#### Locations

Number of locations: Not applicable

Justification for number of locations: No known threats to the species.

Trend: Stable

Extreme fluctuations?: No

#### Population

Number of individuals: Unknown

Trend: Stable

Justification for trend: As it is a widespread species with no specific habitat requirements or known threats, we assume the trend to be stable.

Causes ceased?: Yes

Causes understood?: Yes

Causes reversible?: Yes

Extreme fluctuations?: No

Population Information (Narrative): No population size estimates exist. This species is widespread in South East Asia.

#### Subpopulations

Trend: Stable

Justification for trend: As it is a widespread species with no specific habitat requirements or known threats we assume the trend to be stable.

Extreme fluctuations?: No

Severe fragmentation?: No

#### Habitat

System: Terrestrial

Habitat specialist: Unknown

Habitat (narrative): The predicted range of this species covers the tropical and subtropical moist broadleaf forests and temperate and mixed forests (Olson et al. 2001). Otherwise the preferred habitat is unknown.

Trend in extent, area or quality?: Unknown

##### Habitat

Habitat importance: Major Importance

Habitats: 18. Unknown

#### Habitat

Habitat importance: Major Importance

Habitats: 18. Unknown

#### Ecology

Size: 2.29-3.10 mm

Generation length (yr): 1

Dependency of single sp?: No

Ecology and traits (narrative): Ecology of this particular species is largely unknown. Theridiids in general build space webs which are irregular in shape; threads are often configured in different directions (Jocqué and Dippenaar-Schoeman 2006). These threads tend to break easily when capturing prey. These glue-bearing threads make it difficult for prey to flee and easy for a spider to capture them.

#### Threats

Justification for threats: No known threats.

##### Threats

Threat type: Past

Threats: 12. Other options - Other threat

#### Threats

Threat type: Past

Threats: 12. Other options - Other threat

#### Conservation

Justification for conservation actions: There are several areas of differents sizes and protection levels inside the predicted range of this species (United Nations Environment World Conservation Monitoring Centre 2017).

##### Conservation actions

Conservation action type: In Place

Conservation actions: 1.1. Land/water protection - Site/area protection1.2. Land/water protection - Resource & habitat protection

#### Conservation actions

Conservation action type: In Place

Conservation actions: 1.1. Land/water protection - Site/area protection1.2. Land/water protection - Resource & habitat protection

#### Other

##### Use and trade

Use type: International

Use and trade: 18. Unknown

##### Ecosystem services

Ecosystem service type: Very important

##### Research needed

Research needed: 3.1. Monitoring - Population trends3.4. Monitoring - Habitat trends

Justification for research needed: Monitoring is needed to confirm the current population and habitat trends.

#### Use and trade

Use type: International

Use and trade: 18. Unknown

#### Ecosystem services

Ecosystem service type: Very important

#### Research needed

Research needed: 3.1. Monitoring - Population trends3.4. Monitoring - Habitat trends

Justification for research needed: Monitoring is needed to confirm the current population and habitat trends.

#### Viability analysis

### Thymoites pictipes

#### Species information

Scientific name: Thymoites pictipes

Species authority: (Banks, 1904)

Kingdom: Animalia

Phylum: Arthropoda

Class: Arachnida

Order: Araneae

Family: Theridiidae

Region for assessment: Global

#### Geographic range

Biogeographic realm: Nearctic

Countries: MexicoCanadaUnited States

Map of records (Google Earth): Suppl. material 26

Basis of EOO and AOO: Species Distribution Model

Basis (narrative): Given the relatively high number of records (Gertsch and Archer 1942, Schenkel 1950, Levi 1957), it was possible to perform species distribution modelling (see methods for details).

Min Elevation/Depth (m): 0

Max Elevation/Depth (m): 1060

Range description: This species is known from several sites from the west coast of the USA (Gertsch and Archer 1942, Schenkel 1950, Levi 1957), last recorded prior to 1957 (Levi 1957). In addition, the SDM predicts suitable habitat to be present in Mexico (Baja California) and Canada (British Columbia) as well.

#### New occurrences

#### Extent of occurrence

EOO (km2): 992811

Trend: Unknown

Causes ceased?: Unknown

Causes understood?: Unknown

Causes reversible?: Unknown

Extreme fluctuations?: Unknown

#### Area of occupancy

Trend: Unknown

Causes ceased?: Unknown

Causes understood?: Unknown

Causes reversible?: Unknown

Extreme fluctuations?: Unknown

AOO (km2): 232808

#### Locations

Number of locations: Unknown

Trend: Unknown

Extreme fluctuations?: No

#### Population

Number of individuals: Unknown

Trend: Unknown

Causes ceased?: Unknown

Causes understood?: Unknown

Causes reversible?: Unknown

Extreme fluctuations?: Unknown

Population Information (Narrative): No population size estimates exist.

#### Subpopulations

Trend: Unknown

Extreme fluctuations?: Unknown

Severe fragmentation?: Unknown

#### Habitat

System: Terrestrial

Habitat specialist: Unknown

Habitat (narrative): The habitat of this species is unknown. One specimen was found from tree bark (Levi 1957).

Trend in extent, area or quality?: Unknown

##### Habitat

Habitat importance: Major Importance

Habitats: 18. Unknown

#### Habitat

Habitat importance: Major Importance

Habitats: 18. Unknown

#### Ecology

Size: 2.2-2.4 mm

Generation length (yr): 1

Dependency of single sp?: No

Ecology and traits (narrative): Ecology of this particular species is largely unknown. Theridiids in general build space webs which are irregular in shape; threads are often configured in different directions (Jocqué and Dippenaar-Schoeman 2006). These threads tend to break easily when capturing prey. These glue-bearing threads make it difficult for prey to flee and easy for a spider to capture them.

#### Threats

Justification for threats: Unknown threats.

##### Threats

Threat type: Past

Threats: 12. Other options - Other threat

#### Threats

Threat type: Past

Threats: 12. Other options - Other threat

#### Conservation

Justification for conservation actions: There are several protected areas within the range of this species, namely Redwood National Park and Ventana Wilderness area in USA and El Vizcaíno biosphere reserve in Mexico (United Nations Environment World Conservation Monitoring Centre 2017).

##### Conservation actions

Conservation action type: In Place

Conservation actions: 1.1. Land/water protection - Site/area protection1.2. Land/water protection - Resource & habitat protection

#### Conservation actions

Conservation action type: In Place

Conservation actions: 1.1. Land/water protection - Site/area protection1.2. Land/water protection - Resource & habitat protection

#### Other

##### Use and trade

Use type: International

##### Ecosystem services

Ecosystem service type: Very important

##### Research needed

Research needed: 1.3. Research - Life history & ecology3.1. Monitoring - Population trends3.4. Monitoring - Habitat trends

Justification for research needed: Basic research on the ecology of this species is needed. Also monitoring is needed to know the current population and habitat trends, as it was last recorded before 1957.

#### Use and trade

Use type: International

#### Ecosystem services

Ecosystem service type: Very important

#### Research needed

Research needed: 1.3. Research - Life history & ecology3.1. Monitoring - Population trends3.4. Monitoring - Habitat trends

Justification for research needed: Basic research on the ecology of this species is needed. Also monitoring is needed to know the current population and habitat trends, as it was last recorded before 1957.

#### Viability analysis

### Thymoites verus

#### Species information

Scientific name: Thymoites verus

Species authority: (Levi, 1959)

Kingdom: Animalia

Phylum: Arthropoda

Class: Arachnida

Order: Araneae

Family: Theridiidae

Region for assessment: Global

#### Geographic range

Biogeographic realm: Neotropical

Countries: Mexico

Map of records (Google Earth): Suppl. material 27

Basis of EOO and AOO: Unknown

Basis (narrative): Unknown EOO or AOO.

Min Elevation/Depth (m): 20

Max Elevation/Depth (m): 20

Range description: Known only from the type locality in Santa Cruz, Veracruz, Mexico, recorded once prior to 1959 (Levi 1959). There are many places named Santa Cruz within the Veracruz region in Mexico, hence the coordinates for Veracruz are presented on the map.

#### New occurrences

#### Extent of occurrence

EOO (km2): Unknown

Trend: Unknown

Causes ceased?: Unknown

Causes understood?: Unknown

Causes reversible?: Unknown

Extreme fluctuations?: Unknown

#### Area of occupancy

Trend: Unknown

Causes ceased?: Unknown

Causes understood?: Unknown

Causes reversible?: Unknown

Extreme fluctuations?: Unknown

AOO (km2): Unknown

#### Locations

Number of locations: Unknown

Trend: Unknown

Extreme fluctuations?: Unknown

#### Population

Number of individuals: Unknown

Trend: Unknown

Causes ceased?: Unknown

Causes understood?: Unknown

Causes reversible?: Unknown

Extreme fluctuations?: Unknown

Population Information (Narrative): No population size estimates exist.

#### Subpopulations

Trend: Unknown

Extreme fluctuations?: Unknown

Severe fragmentation?: Unknown

#### Habitat

System: Terrestrial

Habitat specialist: Unknown

Habitat (narrative): Since the type locality is unspecified, the habitat preferred by this species cannot be inferred.

Trend in extent, area or quality?: Unknown

##### Habitat

Habitat importance: Major Importance

Habitats: 18. Unknown

#### Habitat

Habitat importance: Major Importance

Habitats: 18. Unknown

#### Ecology

Size: 2.1 mm

Generation length (yr): 1

Dependency of single sp?: Unknown

Ecology and traits (narrative): Ecology of this particular species is unknown. Theridiids in general build space webs which are irregular in shape; threads are often configured in different directions (Jocqué and Dippenaar-Schoeman 2006). These threads tend to break easily when capturing prey. These glue-bearing threads make it difficult for prey to flee and easy for a spider to capture them.

#### Threats

Justification for threats: Unknown threats.

##### Threats

Threat type: Past

Threats: 12. Other options - Other threat

#### Threats

Threat type: Past

Threats: 12. Other options - Other threat

#### Conservation

##### Conservation actions

#### Conservation actions

#### Other

##### Use and trade

Use type: International

Use and trade: 18. Unknown

##### Ecosystem services

Ecosystem service type: Very important

##### Research needed

Research needed: 1.2. Research - Population size, distribution & trends1.3. Research - Life history & ecology1.5. Research - Threats

Justification for research needed: Basic research is needed to know the current distribution and population size and trends, ecology and traits of the species, along with possible threats.

#### Use and trade

Use type: International

Use and trade: 18. Unknown

#### Ecosystem services

Ecosystem service type: Very important

#### Research needed

Research needed: 1.2. Research - Population size, distribution & trends1.3. Research - Life history & ecology1.5. Research - Threats

Justification for research needed: Basic research is needed to know the current distribution and population size and trends, ecology and traits of the species, along with possible threats.

#### Viability analysis

### Ogulnius infumatus

#### Species information

Scientific name: Ogulnius infumatus

Species authority: Simon, 1898

Kingdom: Animalia

Phylum: Arthropoda

Class: Arachnida

Order: Araneae

Family: Theridiosomatidae

Region for assessment: Global

#### Geographic range

Biogeographic realm: Neotropical

Countries: Saint Vincent and the Grenadines

Map of records (Google Earth): Suppl. material 28

Basis of EOO and AOO: Unknown

Basis (narrative): Unknown EOO or AOO.

Min Elevation/Depth (m): 450

Max Elevation/Depth (m): 450

Range description: Simon (1897) did not specify a locality for the holotype of *Ogulnius infumatus* except for the island of Saint Vincent in the Caribbean. The species has not been reported since.

#### New occurrences

#### Extent of occurrence

EOO (km2): Unknown

Trend: Unknown

Causes ceased?: Unknown

Causes understood?: Unknown

Causes reversible?: Unknown

Extreme fluctuations?: Unknown

#### Area of occupancy

Trend: Unknown

Causes ceased?: Unknown

Causes understood?: Unknown

Causes reversible?: Unknown

Extreme fluctuations?: Unknown

AOO (km2): Unknown

#### Locations

Number of locations: Unknown

Trend: Unknown

Extreme fluctuations?: Unknown

#### Population

Number of individuals: Unknown

Trend: Unknown

Causes ceased?: Unknown

Causes understood?: Unknown

Causes reversible?: Unknown

Extreme fluctuations?: Unknown

Population Information (Narrative): No population size estimates exist.

#### Subpopulations

Trend: Unknown

Extreme fluctuations?: Unknown

Severe fragmentation?: Unknown

#### Habitat

System: Terrestrial

Habitat specialist: Unknown

Habitat (narrative): The habitat of this particular species is unknown. Theridiosomatids in general have been observed to prefer wet and humid habitats, for example dark forests and some have been recorded from caves as well (Coddington 1986).

Trend in extent, area or quality?: Unknown

##### Habitat

Habitat importance: Major Importance

Habitats: 18. Unknown

#### Habitat

Habitat importance: Major Importance

Habitats: 18. Unknown

#### Ecology

Size: 0.5 mm

Generation length (yr): 1

Dependency of single sp?: No

Ecology and traits (narrative): Ecology of this species is largely unknown. Theridiosomatids in general tend to build a web that varies in shape (complete orb webs to networks with a few threads) and some species do not build a web at all. Webs are often built in litter or in low vegetation (Coddington 1986, Dippenaar-Schoeman and Jocqué 1997).

#### Threats

Justification for threats: Unknown threats.

##### Threats

Threat type: Past

Threats: 12. Other options - Other threat

#### Threats

Threat type: Past

Threats: 12. Other options - Other threat

#### Conservation

##### Conservation actions

#### Conservation actions

#### Other

##### Use and trade

Use type: International

Use and trade: 18. Unknown

##### Ecosystem services

Ecosystem service type: Very important

##### Research needed

Research needed: 1.2. Research - Population size, distribution & trends1.3. Research - Life history & ecology1.5. Research - Threats

Justification for research needed: Basic research is needed to know the current distribution and population size and trends, ecology and traits of the species, along with possible threats.

#### Use and trade

Use type: International

Use and trade: 18. Unknown

#### Ecosystem services

Ecosystem service type: Very important

#### Research needed

Research needed: 1.2. Research - Population size, distribution & trends1.3. Research - Life history & ecology1.5. Research - Threats

Justification for research needed: Basic research is needed to know the current distribution and population size and trends, ecology and traits of the species, along with possible threats.

#### Viability analysis

### Theridiosoma concolor

#### Species information

Scientific name: Theridiosoma concolor

Species authority: Keyserling, 1884

Kingdom: Animalia

Phylum: Arthropoda

Class: Arachnida

Order: Araneae

Family: Theridiosomatidae

Region for assessment: Global

#### Geographic range

Biogeographic realm: Neotropical

Countries: Peru

Map of records (Google Earth): Suppl. material 29

Basis of EOO and AOO: Unknown

Basis (narrative): Unknown EOO or AOO.

Min Elevation/Depth (m): 120

Max Elevation/Depth (m): 120

Range description: The species is only known from the type locality, from a single collection dated over 130 years ago (Keyserling 1884).

#### New occurrences

#### Extent of occurrence

EOO (km2): Unknown

Trend: Unknown

Causes ceased?: Unknown

Causes understood?: Unknown

Causes reversible?: Unknown

Extreme fluctuations?: Unknown

#### Area of occupancy

Trend: Unknown

Causes ceased?: Unknown

Causes understood?: Unknown

Causes reversible?: Unknown

Extreme fluctuations?: Unknown

AOO (km2): Unknown

#### Locations

Number of locations: Unknown

Justification for number of locations: 

Trend: Unknown

Extreme fluctuations?: Unknown

#### Population

Number of individuals: Unknown

Trend: Unknown

Causes ceased?: Unknown

Causes understood?: Unknown

Causes reversible?: Unknown

Extreme fluctuations?: Unknown

Population Information (Narrative): This species has been collected only once and data on its population size, fluctuations or changes are not known (Keyserling 1884).

#### Subpopulations

Trend: Unknown

Extreme fluctuations?: Unknown

Severe fragmentation?: No

#### Habitat

System: Terrestrial

Habitat specialist: Unknown

Habitat (narrative): The area in which the species was collected is dominated by tropical rainforest (Keyserling 1884).

Trend in extent, area or quality?: Decline (estimated)

Justification for trend: This habitat is estimated to be decreasing based on satellite data (Bachman et al. 2011, Global Forest Watch 2014).

##### Habitat

Habitat importance: Major Importance

Habitats: 1.5. Forest - Subtropical/Tropical Dry1.9. Forest - Subtropical/Tropical Moist Montane

#### Habitat

Habitat importance: Major Importance

Habitats: 1.5. Forest - Subtropical/Tropical Dry1.9. Forest - Subtropical/Tropical Moist Montane

#### Ecology

Size: 4 mm

Generation length (yr): 1

Dependency of single sp?: No

Ecology and traits (narrative): Ecology of this species is largely unknown. Theridiosomatids in general tend to build a web that varies in shape (complete orb webs to networks with a few threads) and some species do not build a web at all. Webs are often built in litter or in low vegetation (Coddington 1986, Dippenaar-Schoeman and Jocqué 1997).

#### Threats

Justification for threats: This species is known only from a forest area that is estimated to have recently decreased (since 2004 based on satellite imagery data) due to an increase in fire frequency (Bachman et al. 2011) and deforestation (Global Forest Watch 2014).

##### Threats

Threat type: Ongoing

Threats: 7.1.1. Natural system modifications - Fire & fire suppression - Increase in fire frequency/intensity5.3.5. Biological resource use - Logging & wood harvesting - Motivation unknown/unrecorded

#### Threats

Threat type: Ongoing

Threats: 7.1.1. Natural system modifications - Fire & fire suppression - Increase in fire frequency/intensity5.3.5. Biological resource use - Logging & wood harvesting - Motivation unknown/unrecorded

#### Conservation

Justification for conservation actions: This species was collected in an area of tropical rainforest that has been partially deforested (Global Forest Watch 2014). This area of tropical rainforest has also been shown to have been affected by fire, likely set by nearby human populations to aid in deforestation (Bachman et al. 2011). Although the population values are unknown, it is likely that drastic deforestation and fire in this species habitat would be detrimental to its survival and increase its extinction risk. It is therefore recommended that deforestation and fire in this habitat be carefully managed.

##### Conservation actions

Conservation action type: Needed

Conservation actions: 1.1. Land/water protection - Site/area protection1.2. Land/water protection - Resource & habitat protection2.1. Land/water management - Site/area management

#### Conservation actions

Conservation action type: Needed

Conservation actions: 1.1. Land/water protection - Site/area protection1.2. Land/water protection - Resource & habitat protection2.1. Land/water management - Site/area management

#### Other

##### Use and trade

Use type: International

Use and trade: 18. Unknown

##### Ecosystem services

Ecosystem service type: Very important

##### Research needed

Research needed: 1.2. Research - Population size, distribution & trends1.3. Research - Life history & ecology1.5. Research - Threats3.4. Monitoring - Habitat trends

Justification for research needed: Basic research is needed to know the current distribution and population size and trends, ecology and traits of the species, along with possible threats. The single locality where the species was collected is affected by deforestation and monitoring of human activity should be conducted.

#### Use and trade

Use type: International

Use and trade: 18. Unknown

#### Ecosystem services

Ecosystem service type: Very important

#### Research needed

Research needed: 1.2. Research - Population size, distribution & trends1.3. Research - Life history & ecology1.5. Research - Threats3.4. Monitoring - Habitat trends

Justification for research needed: Basic research is needed to know the current distribution and population size and trends, ecology and traits of the species, along with possible threats. The single locality where the species was collected is affected by deforestation and monitoring of human activity should be conducted.

#### Viability analysis

Justification for probability: 

### Bomis bengalensis

#### Species information

Scientific name: Bomis bengalensis

Species authority: Tikader, 1962

Kingdom: Animaia

Phylum: Arthropoda

Class: Arachnida

Order: Araneae

Family: Thomisidae

Region for assessment: Global

#### Geographic range

Biogeographic realm: Indomalayan

Countries: India

Map of records (Google Earth): Suppl. material 30

Basis of EOO and AOO: Unknown

Basis (narrative): Unknown EOO or AOO.

Min Elevation/Depth (m): 10

Max Elevation/Depth (m): 20

Range description: Known from only two sites in West Bengal, India, recorded during the 1950s (Tikader 1962, Tikader and Biswas 1981).

#### New occurrences

#### Extent of occurrence

EOO (km2): Unknown

Trend: Unknown

Causes ceased?: Unknown

Causes understood?: Unknown

Causes reversible?: Unknown

Extreme fluctuations?: Unknown

#### Area of occupancy

Trend: Unknown

Causes ceased?: Unknown

Causes understood?: Unknown

Causes reversible?: Unknown

Extreme fluctuations?: Unknown

AOO (km2): Unknown

#### Locations

Number of locations: Unknown

Trend: Unknown

Extreme fluctuations?: Unknown

#### Population

Number of individuals: Unknown

Trend: Unknown

Causes ceased?: Unknown

Causes understood?: Unknown

Causes reversible?: Unknown

Extreme fluctuations?: Unknown

Population Information (Narrative): No population size estimates exist.

#### Subpopulations

Trend: Unknown

Extreme fluctuations?: Unknown

Severe fragmentation?: Unknown

#### Habitat

System: Terrestrial

Habitat specialist: Unknown

Habitat (narrative): The records for this species have been made in a region dominated by tropical and subtropical moist broadleaf forests near mangroves (Olson et al. 2001). Otherwise the habitat requirements of this particular species remain unknown.

Trend in extent, area or quality?: Unknown

##### Habitat

Habitat importance: Major Importance

Habitats: 18. Unknown

#### Habitat

Habitat importance: Major Importance

Habitats: 18. Unknown

#### Ecology

Size: 3.1 mm

Generation length (yr): 1

Dependency of single sp?: Unknown

Ecology and traits (narrative): Ecology of this species is unknown. Thomisids in general are ambush predators and do not build webs. Also known as crab spiders, they are most active during the day and usually wear a cryptic colour which help them to camouflage and wait for their prey, for example, by sitting on a plant. With their acute vision, they detect the prey and then attack. The prey are sometimes over twice the size of the spider and are paralysed with strong venom (Dippenaar-Schoeman and Jocqué 1997).

#### Threats

Justification for threats: Unknown threats.

##### Threats

Threat type: Past

Threats: 12. Other options - Other threat

#### Threats

Threat type: Past

Threats: 12. Other options - Other threat

#### Conservation

##### Conservation actions

#### Conservation actions

#### Other

##### Use and trade

Use type: International

Use and trade: 18. Unknown

##### Ecosystem services

Ecosystem service type: Very important

##### Research needed

Research needed: 1.2. Research - Population size, distribution & trends1.3. Research - Life history & ecology1.5. Research - Threats

Justification for research needed: Basic research is needed to know the current distribution and population size and trends, ecology and traits of the species, along with possible threats.

#### Use and trade

Use type: International

Use and trade: 18. Unknown

#### Ecosystem services

Ecosystem service type: Very important

#### Research needed

Research needed: 1.2. Research - Population size, distribution & trends1.3. Research - Life history & ecology1.5. Research - Threats

Justification for research needed: Basic research is needed to know the current distribution and population size and trends, ecology and traits of the species, along with possible threats.

#### Viability analysis

### Epicadus trituberculatus

#### Species information

Scientific name: Epicadus trituberculatus

Species authority: 

Synonyms: *Tobias paraguayensis* Mello-Leitão, 1929*Epicadus planus* Mello-Leitão, 1932

Kingdom: Animalia

Phylum: Arthropoda

Class: Arachnida

Order: Araneae

Family: Thomisidae

Region for assessment: Global

#### Geographic range

Biogeographic realm: Neotropical

Countries: GuyanaFrench GuianaSurinameParaguayPeruTrinidad and TobagoGuatemalaBelizePanamaBrazilColombiaEcuadorArgentinaMexicoCosta RicaHondurasNicaraguaBolivia, Plurinational States ofVenezuela, Bolivarian Republic of

Map of records (Google Earth): Suppl. material 31

Basis of EOO and AOO: Species Distribution Model

Basis (narrative): Given the relatively high number of records (Taczanowski 1872, Mello-Leitão 1929, Silva-Moreira and Machado 2016), it was possible to perform species distribution modelling (see methods for details).

Min Elevation/Depth (m): 0

Max Elevation/Depth (m): 1750

Range description: *E. trituberculatus* is known from several sites in South America (Taczanowski 1872, Mello-Leitão 1929, Silva-Moreira and Machado 2016) and should be present throughout tropical Central and South Americas.

#### New occurrences

#### Extent of occurrence

EOO (km2): 17139271

Trend: Stable

Justification for trend: As it is a widespread species with no known threats, the trend is assumed to be stable.

Causes ceased?: Yes

Causes understood?: Yes

Causes reversible?: Yes

Extreme fluctuations?: No

#### Area of occupancy

Trend: Stable

Justification for trend: As it is a widespread species with no known threats, the trend is assumed to be stable.

Causes ceased?: Yes

Causes understood?: Yes

Causes reversible?: Yes

Extreme fluctuations?: No

AOO (km2): 3899420

#### Locations

Number of locations: Not applicable

Justification for number of locations: No known threats to the species.

Trend: Stable

Extreme fluctuations?: No

#### Population

Number of individuals: Unknown

Trend: Stable

Causes ceased?: Yes

Causes understood?: Yes

Causes reversible?: Yes

Extreme fluctuations?: No

Population Information (Narrative): No population size estimates exist. This species should be widespread and with no known threats, therefore the trend is assumed to be stable.

#### Subpopulations

Number of subpopulations: Unknown

Trend: Stable

Justification for trend: As it is a widespread species with no known threats, the trend is assumed to be stable.

Extreme fluctuations?: No

Severe fragmentation?: No

#### Habitat

System: Terrestrial

Habitat specialist: No

Habitat (narrative): No habitat data for this species is reported. Its predicted range is mostly covered by tropical and subtropical moist broadleaf forests (Olson et al. 2001).

Trend in extent, area or quality?: Unknown

##### Habitat

Habitat importance: Major Importance

Habitats: 18. Unknown

#### Habitat

Habitat importance: Major Importance

Habitats: 18. Unknown

#### Ecology

Size: 2.50-15 mm

Generation length (yr): 1

Dependency of single sp?: No

Ecology and traits (narrative): Ecology of this species is unknown. Thomisids in general are ambush predators and do not build webs. Also known as crab spiders, they are most active during the day and usually wear a cryptic colour which help them to camouflage and wait for their prey, for example, by sitting on a plant. With their acute vision, they detect the prey and then attack. The prey are sometimes over twice the size of the spider and are paralysed with strong venom (Dippenaar-Schoeman and Jocqué 1997). *Epicadus* species are medium-sized spiders with a remarkable sexual size dimorphism, commonly found on leaves or flowers (Silva-Moreira and Machado 2016). According to Machado et al. (2017), the genus *Epicadus* is included in a clade of spiders that display a variety of polychromatism and use flowers to hunt.

#### Threats

Justification for threats: No known threats.

##### Threats

Threat type: Past

Threats: 12. Other options - Other threat

#### Threats

Threat type: Past

Threats: 12. Other options - Other threat

#### Conservation

Justification for conservation actions: There are several protected areas within the range of this species (United Nations Environment World Conservation Monitoring Centre 2017).

##### Conservation actions

Conservation action type: In Place

Conservation actions: 1.1. Land/water protection - Site/area protection1.2. Land/water protection - Resource & habitat protection

#### Conservation actions

Conservation action type: In Place

Conservation actions: 1.1. Land/water protection - Site/area protection1.2. Land/water protection - Resource & habitat protection

#### Other

##### Use and trade

Use type: International

Use and trade: 18. Unknown

##### Ecosystem services

Ecosystem service type: Very important

##### Research needed

Research needed: 3.1. Monitoring - Population trends3.4. Monitoring - Habitat trends

Justification for research needed: Monitoring is needed to confirm the current population and habitat trends.

#### Use and trade

Use type: International

Use and trade: 18. Unknown

#### Ecosystem services

Ecosystem service type: Very important

#### Research needed

Research needed: 3.1. Monitoring - Population trends3.4. Monitoring - Habitat trends

Justification for research needed: Monitoring is needed to confirm the current population and habitat trends.

#### Viability analysis

### Misumena picta

#### Species information

Scientific name: Misumena picta

Species authority: Franganillo, 1926

Kingdom: Animalia

Phylum: Arthropoda

Class: Arachnida

Order: Araneae

Family: Thomisidae

Region for assessment: Global

#### Geographic range

Biogeographic realm: Neotropical

Countries: Cuba

Map of records (Google Earth): Suppl. material 32

Basis of EOO and AOO: Unknown

Basis (narrative): Unknown EOO or AOO.

Min Elevation/Depth (m): 40

Max Elevation/Depth (m): 100

Range description: Recorded only from two localities, Habana and Camaguey in Cuba, in 1926 (Franganillo Balboa 1926).

#### New occurrences

#### Extent of occurrence

EOO (km2): Unknown

Trend: Unknown

Causes ceased?: Unknown

Causes understood?: Unknown

Causes reversible?: Unknown

Extreme fluctuations?: Unknown

#### Area of occupancy

Trend: Unknown

Causes ceased?: Unknown

Causes understood?: Unknown

Causes reversible?: Unknown

Extreme fluctuations?: Unknown

AOO (km2): Unknown

#### Locations

Number of locations: Unknown

Trend: Unknown

Extreme fluctuations?: Unknown

#### Population

Number of individuals: Unknown

Trend: Unknown

Causes ceased?: Unknown

Causes understood?: Unknown

Causes reversible?: Unknown

Extreme fluctuations?: Unknown

#### Subpopulations

Trend: Unknown

Extreme fluctuations?: Unknown

Severe fragmentation?: Unknown

#### Habitat

System: Terrestrial

Habitat specialist: Unknown

Habitat (narrative): Cuba is mostly covered with tropical and subtropical dry broadleaf forests (Olson et al. 2001). Otherwise the habitat requirements of this particular species remain unknown.

Trend in extent, area or quality?: Unknown

##### Habitat

Habitat importance: Major Importance

Habitats: 18. Unknown

#### Habitat

Habitat importance: Major Importance

Habitats: 18. Unknown

#### Ecology

Size: Unknown

Generation length (yr): 1

Dependency of single sp?: No

Ecology and traits (narrative): Ecology of this species is unknown. Thomisids in general are ambush predators and do not build webs. Also known as crab spiders, they are most active during the day and usually wear a cryptic colour which help them to camouflage and wait for their prey, for example, by sitting on a plant. With their acute vision, they detect the prey and then attack. The prey are sometimes over twice the size of the spider and are paralysed with strong venom (Dippenaar-Schoeman and Jocqué 1997).

#### Threats

Justification for threats: Unknown threats.

##### Threats

Threat type: Past

Threats: 12. Other options - Other threat

#### Threats

Threat type: Past

Threats: 12. Other options - Other threat

#### Conservation

##### Conservation actions

#### Conservation actions

#### Other

##### Use and trade

Use type: International

Use and trade: 18. Unknown

##### Ecosystem services

Ecosystem service type: Very important

##### Research needed

Research needed: 1.2. Research - Population size, distribution & trends1.3. Research - Life history & ecology1.5. Research - Threats

Justification for research needed: Basic research is needed to know the current distribution and population size and trends, ecology and traits of the species, along with possible threats.

#### Use and trade

Use type: International

Use and trade: 18. Unknown

#### Ecosystem services

Ecosystem service type: Very important

#### Research needed

Research needed: 1.2. Research - Population size, distribution & trends1.3. Research - Life history & ecology1.5. Research - Threats

Justification for research needed: Basic research is needed to know the current distribution and population size and trends, ecology and traits of the species, along with possible threats.

#### Viability analysis

### Misumenoides gwarighatensis

#### Species information

Scientific name: Misumenoides gwarighatensis

Species authority: Gajbe, 2004

Kingdom: Animalia

Phylum: Arthropoda

Class: Arachnida

Order: Araneae

Family: Thomisidae

Region for assessment: Global

#### Geographic range

Biogeographic realm: Indomalayan

Countries: India

Map of records (Google Earth): Suppl. material 33

Basis of EOO and AOO: Unknown

Basis (narrative): Unknown EOO or AOO.

Min Elevation/Depth (m): 390

Max Elevation/Depth (m): 390

Range description: Known only from the type locality Madhya Pradesh, India. Recorded only once in 1997 (Gajbe 2004).

#### New occurrences

#### Extent of occurrence

EOO (km2): Unknown

Trend: Unknown

Causes ceased?: Unknown

Causes understood?: Unknown

Causes reversible?: Unknown

Extreme fluctuations?: Unknown

#### Area of occupancy

Trend: Unknown

Causes ceased?: Unknown

Causes understood?: Unknown

Causes reversible?: Unknown

Extreme fluctuations?: Unknown

AOO (km2): Unknown

#### Locations

Number of locations: Unknown

Trend: Unknown

Extreme fluctuations?: Unknown

#### Population

Number of individuals: Unknown

Trend: Unknown

Causes ceased?: Unknown

Causes understood?: Unknown

Causes reversible?: Unknown

Extreme fluctuations?: Unknown

Population Information (Narrative): No population size estimates exist.

#### Subpopulations

Trend: Unknown

Extreme fluctuations?: Unknown

Severe fragmentation?: Unknown

#### Habitat

System: Terrestrial

Habitat specialist: Unknown

Habitat (narrative): The habitat of this species is largely unknown, the single specimen being found in vegetation (Gajbe 2004). The type locality seems to fall into the ecoregion of tropical and subtropical dry broadleaf forest (Olson et al. 2001).

Trend in extent, area or quality?: Unknown

##### Habitat

Habitat importance: Major Importance

Habitats: 18. Unknown

#### Habitat

Habitat importance: Major Importance

Habitats: 18. Unknown

#### Ecology

Size: 6.5 mm

Generation length (yr): 1

Dependency of single sp?: No

Ecology and traits (narrative): Ecology of this species is unknown. Thomisids in general are ambush predators and do not build webs. Also known as crab spiders, they are most active during the day and usually wear a cryptic colour which help them to camouflage and wait for their prey, for example, by sitting on a plant. With their acute vision, they detect the prey and then attack. The prey are sometimes over twice the size of the spider and are paralysed with strong venom (Dippenaar-Schoeman and Jocqué 1997).

#### Threats

Justification for threats: Unknown threats.

##### Threats

Threat type: Past

Threats: 12. Other options - Other threat

#### Threats

Threat type: Past

Threats: 12. Other options - Other threat

#### Conservation

##### Conservation actions

#### Conservation actions

#### Other

##### Use and trade

Use type: International

Use and trade: 18. Unknown

##### Ecosystem services

Ecosystem service type: Very important

##### Research needed

Research needed: 1.2. Research - Population size, distribution & trends1.3. Research - Life history & ecology1.5. Research - Threats

Justification for research needed: Basic research is needed to know the current distribution and population size and trends, ecology and traits of the species, along with possible threats.

#### Use and trade

Use type: International

Use and trade: 18. Unknown

#### Ecosystem services

Ecosystem service type: Very important

#### Research needed

Research needed: 1.2. Research - Population size, distribution & trends1.3. Research - Life history & ecology1.5. Research - Threats

Justification for research needed: Basic research is needed to know the current distribution and population size and trends, ecology and traits of the species, along with possible threats.

#### Viability analysis

### Misumenops guianensis

#### Species information

Scientific name: Misumenops guianensis

Species authority: (Taczanowski, 1872)

Kingdom: Animalia

Phylum: Arthropoda

Class: Arachnida

Order: Araneae

Family: Thomisidae

Region for assessment: Global

#### Geographic range

Biogeographic realm: Neotropical

Countries: GuyanaFrench GuianaSurinameParaguayPeruTrinidad and TobagoPanamaBrazilColombiaArgentinaCosta RicaBolivia, Plurinational States ofVenezuela, Bolivarian Republic of

Map of records (Google Earth): Suppl. material 34

Basis of EOO and AOO: Species Distribution Model

Basis (narrative): Given the relatively high number of records (Taczanowski 1872, Badcock 1932, Schenkel 1949, Lehtinen and Marusik 2008), it was possible to perform species distribution modelling (see methods for details).

Min Elevation/Depth (m): 0

Max Elevation/Depth (m): 1270

Range description: This species should be widely distributed throughout South America (Taczanowski 1872, Badcock 1932, Schenkel 1949, Lehtinen and Marusik 2008).

#### New occurrences

#### Extent of occurrence

EOO (km2): 15958592

Trend: Stable

Justification for trend: As it is a widespread species with no known threats, the trend is assumed to be stable.

Causes ceased?: Yes

Causes understood?: Yes

Causes reversible?: Yes

Extreme fluctuations?: No

#### Area of occupancy

Trend: Stable

Justification for trend: As it is a widespread species with no known threats, the trend is assumed to be stable.

Causes ceased?: Yes

Causes understood?: Yes

Causes reversible?: Yes

Extreme fluctuations?: No

AOO (km2): 11867792

#### Locations

Number of locations: Not applicable

Justification for number of locations: No known threats to the species.

Trend: Stable

Extreme fluctuations?: No

#### Population

Number of individuals: Unknown

Trend: Stable

Justification for trend: As it is a widespread species with no known threats, the trend is assumed to be stable.

Causes ceased?: Yes

Causes understood?: Yes

Causes reversible?: Yes

Extreme fluctuations?: No

Population Information (Narrative): No population size estimates exist. As it is a widespread species with no known threats, the trend is assumed to be stable.

#### Subpopulations

Trend: Stable

Justification for trend: As it is a widespread species with no known threats, the trend is assumed to be stable.

Extreme fluctuations?: No

Severe fragmentation?: No

#### Habitat

System: Terrestrial

Habitat specialist: Unknown

Habitat (narrative): Specimens have been collected from savannahs, dry meadows and from a gallery forest in Venezuela (Lehtinen and Marusik 2008).

Trend in extent, area or quality?: Stable

Justification for trend: Given the variety of habitat types, the quality is assumed to be stable.

##### Habitat

Habitat importance: Major Importance

Habitats: 1.6. Forest - Subtropical/Tropical Moist Lowland2.1. Savanna - Dry4.5. Grassland - Subtropical/Tropical Dry

#### Habitat

Habitat importance: Major Importance

Habitats: 1.6. Forest - Subtropical/Tropical Moist Lowland2.1. Savanna - Dry4.5. Grassland - Subtropical/Tropical Dry

#### Ecology

Size: 3.1–5.7 mm

Generation length (yr): 1

Dependency of single sp?: No

Ecology and traits (narrative): Ecology of this species is unknown. Thomisids in general are ambush predators and do not build webs. Also known as crab spiders, they are most active during the day and usually wear a cryptic colour which help them to camouflage and wait for their prey, for example, by sitting on a plant. With their acute vision, they detect the prey and then attack. The prey are sometimes over twice the size of the spider and are paralysed with strong venom (Dippenaar-Schoeman and Jocqué 1997).

#### Threats

Justification for threats: No known threats.

##### Threats

Threat type: Past

Threats: 12. Other options - Other threat

#### Threats

Threat type: Past

Threats: 12. Other options - Other threat

#### Conservation

Justification for conservation actions: There are several protected areas within the range of this species (United Nations Environment World Conservation Monitoring Centre 2017).

##### Conservation actions

Conservation action type: In Place

Conservation actions: 1.1. Land/water protection - Site/area protection1.2. Land/water protection - Resource & habitat protection

#### Conservation actions

Conservation action type: In Place

Conservation actions: 1.1. Land/water protection - Site/area protection1.2. Land/water protection - Resource & habitat protection

#### Other

##### Use and trade

Use type: International

Use and trade: 18. Unknown

##### Ecosystem services

Ecosystem service type: Very important

##### Research needed

Research needed: 3.1. Monitoring - Population trends3.4. Monitoring - Habitat trends

Justification for research needed: Monitoring is needed to confirm the current population and habitat trends.

#### Use and trade

Use type: International

Use and trade: 18. Unknown

#### Ecosystem services

Ecosystem service type: Very important

#### Research needed

Research needed: 3.1. Monitoring - Population trends3.4. Monitoring - Habitat trends

Justification for research needed: Monitoring is needed to confirm the current population and habitat trends.

#### Viability analysis

### Misumenops ignobilis

#### Species information

Scientific name: Misumenops ignobilis

Species authority: (Badcock, 1932)

Kingdom: Animalia

Phylum: Arthropoda

Class: Arachnida

Order: Araneae

Family: Thomisidae

Region for assessment: Global

#### Geographic range

Biogeographic realm: Neotropical

Countries: ParaguayArgentina

Map of records (Google Earth): Suppl. material 35

Basis of EOO and AOO: Unknown

Basis (narrative): Unknown EOO or AOO.

Min Elevation/Depth (m): 50

Max Elevation/Depth (m): 290

Range description: This species is known from Paraguay and Argentina, specifically in the Gran Chaco on the Paraguayan side of the Bolivian border, recorded in 1940 (Badcock 1932) and the Argentinian Chaco, recorded in 1927 (Schenkel 1949).

#### New occurrences

#### Extent of occurrence

EOO (km2): Unknown

Trend: Unknown

Causes ceased?: Unknown

Causes understood?: Unknown

Causes reversible?: Unknown

Extreme fluctuations?: Unknown

#### Area of occupancy

Trend: Unknown

Causes ceased?: Unknown

Causes understood?: Unknown

Causes reversible?: Unknown

Extreme fluctuations?: Unknown

AOO (km2): Unknown

#### Locations

Number of locations: Unknown

Trend: Unknown

Extreme fluctuations?: Unknown

#### Population

Number of individuals: Unknown

Trend: Unknown

Causes ceased?: Unknown

Causes understood?: Unknown

Causes reversible?: Unknown

Extreme fluctuations?: Unknown

Population Information (Narrative): No population size estimates exist.

#### Subpopulations

Trend: Unknown

Extreme fluctuations?: No

Severe fragmentation?: No

#### Habitat

System: Terrestrial

Habitat specialist: Unknown

Habitat (narrative): The localities where this species has been recorded fall between tropical and subtropical dry broadleaf forests and grasslands, savannahs and shrublands (Olson et al. 2001). Otherwise the habitat requirements of this particular species remain unknown.

Trend in extent, area or quality?: Unknown

##### Habitat

Habitat importance: Major Importance

Habitats: 18. Unknown

#### Habitat

Habitat importance: Major Importance

Habitats: 18. Unknown

#### Ecology

Size: Unknown

Generation length (yr): 1

Dependency of single sp?: No

Ecology and traits (narrative): Ecology of this species is unknown. Thomisids in general are ambush predators and do not build webs. Also known as crab spiders, they are most active during the day and usually wear a cryptic colour which help them to camouflage and wait for their prey, for example, by sitting on a plant. With their acute vision, they detect the prey and then attack. The prey are sometimes over twice the size of the spider and are paralysed with strong venom (Dippenaar-Schoeman and Jocqué 1997).

#### Threats

Justification for threats: In the last 30 years, the Gran Chaco region has shown a massive contraction of forest, where 1.2 million ha of original lowland and mountain subtropical dry forest, 85% of the original, have been cleared, mainly due to agricultural expansion (Global Forest Watch 2014). This habitat change may possibly affect the species population, if it lives in forests.

##### Threats

Threat type: Ongoing

Threats: 2.3.2. Agriculture & aquaculture - Livestock farming & ranching - Small-holder grazing, ranching or farming

#### Threats

Threat type: Ongoing

Threats: 2.3.2. Agriculture & aquaculture - Livestock farming & ranching - Small-holder grazing, ranching or farming

#### Conservation

##### Conservation actions

#### Conservation actions

#### Other

##### Use and trade

Use type: International

Use and trade: 18. Unknown

##### Ecosystem services

Ecosystem service type: Very important

##### Research needed

Research needed: 1.2. Research - Population size, distribution & trends1.3. Research - Life history & ecology1.5. Research - Threats

Justification for research needed: Basic research is needed to know the current distribution and population size and trends, ecology and traits of the species, along with possible threats.

#### Use and trade

Use type: International

Use and trade: 18. Unknown

#### Ecosystem services

Ecosystem service type: Very important

#### Research needed

Research needed: 1.2. Research - Population size, distribution & trends1.3. Research - Life history & ecology1.5. Research - Threats

Justification for research needed: Basic research is needed to know the current distribution and population size and trends, ecology and traits of the species, along with possible threats.

#### Viability analysis

### Oxytate greenae

#### Species information

Scientific name: Oxytate greenae

Species authority: (Tikader, 1980)

Kingdom: Animalia

Phylum: Arthropoda

Class: Arachnida

Order: Araneae

Family: Thomisidae

Region for assessment: Global

#### Geographic range

Biogeographic realm: Indomalayan

Countries: BangladeshBhutanNepalIndiaMyanmar

Map of records (Google Earth): Suppl. material 36

Basis of EOO and AOO: Species Distribution Model

Basis (narrative): Although there were few records (Tikader 1980, Sen et al. 2015), it was possible to perform species distribution modeling (see methods for details).

Min Elevation/Depth (m): 0

Max Elevation/Depth (m): 1770

Range description: This species is known from three sites in India; it was recorded in 1971 from Andmana Islands and in 2009 from Kalijhora and Budhuram (Tikader 1980, Sen et al. 2015). However, the species distribution model predicts the existence of suitable habitat in neighbouring regions and countries.

#### New occurrences

#### Extent of occurrence

EOO (km2): 594860

Trend: Unknown

Causes ceased?: Unknown

Causes understood?: Unknown

Causes reversible?: Unknown

Extreme fluctuations?: Unknown

#### Area of occupancy

Trend: Unknown

Causes ceased?: Unknown

Causes understood?: Unknown

Causes reversible?: Unknown

Extreme fluctuations?: Unknown

AOO (km2): 162004

#### Locations

Number of locations: Unknown

Trend: Unknown

Extreme fluctuations?: Unknown

#### Population

Number of individuals: Unknown

Trend: Unknown

Causes ceased?: Unknown

Causes understood?: Unknown

Causes reversible?: Unknown

Extreme fluctuations?: Unknown

Population Information (Narrative): No population size estimates exist.

#### Subpopulations

Trend: Unknown

Extreme fluctuations?: No

Severe fragmentation?: Unknown

#### Habitat

System: Terrestrial

Habitat specialist: No

Habitat (narrative): Unknown habitat. The predicted range falls into the ecoregion of tropical and subtropical moist broadleaf forests (Olson et al. 2001). However, it remains unknown what kinds of habitats this particular species prefers.

Trend in extent, area or quality?: Unknown

##### Habitat

Habitat importance: Major Importance

Habitats: 18. Unknown

#### Habitat

Habitat importance: Major Importance

Habitats: 18. Unknown

#### Ecology

Size: 10 mm

Generation length (yr): 1

Dependency of single sp?: No

Ecology and traits (narrative): Ecology of this species is unknown. Thomisids in general are ambush predators and do not build webs. Also known as crab spiders, they are most active during the day and usually wear a cryptic colour which help them to camouflage and wait for their prey, for example, by sitting on a plant. With their acute vision, they detect the prey and then attack. The prey are sometimes over twice the size of the spider and are paralysed with strong venom (Dippenaar-Schoeman and Jocqué 1997).

#### Threats

Justification for threats: Unknown threats.

##### Threats

Threat type: Past

Threats: 12. Other options - Other threat

#### Threats

Threat type: Past

Threats: 12. Other options - Other threat

#### Conservation

Justification for conservation actions: At least part of the range of this species is within protected areas: according to Sen et al. (2015), this species was recorded from reserve forests, namely Mahananda Wildlife Sanctuary and Gurumara National Park.

##### Conservation actions

Conservation action type: In Place

Conservation actions: 1.1. Land/water protection - Site/area protection1.2. Land/water protection - Resource & habitat protection

#### Conservation actions

Conservation action type: In Place

Conservation actions: 1.1. Land/water protection - Site/area protection1.2. Land/water protection - Resource & habitat protection

#### Other

##### Use and trade

Use type: International

##### Ecosystem services

Ecosystem service type: Very important

##### Research needed

Research needed: 3.1. Monitoring - Population trends3.4. Monitoring - Habitat trends

Justification for research needed: Monitoring is needed to know the current population and habitat trends.

#### Use and trade

Use type: International

#### Ecosystem services

Ecosystem service type: Very important

#### Research needed

Research needed: 3.1. Monitoring - Population trends3.4. Monitoring - Habitat trends

Justification for research needed: Monitoring is needed to know the current population and habitat trends.

#### Viability analysis

### Ozyptila conspurcata

#### Species information

Scientific name: Ozyptila conspurcata

Species authority: Thorell, 1877

Kingdom: Animalia

Phylum: Arthropoda

Class: Arachnida

Order: Araneae

Family: Thomisidae

Region for assessment: Global

#### Geographic range

Biogeographic realm: Nearctic

Countries: CanadaUnited States

Map of records (Google Earth): Suppl. material 37

Basis of EOO and AOO: Species Distribution Model

Basis (narrative): Given the relatively high number of records (Thorell 1877, Banks 1892, Emerton 1894, Gertsch 1939, Kaston 1948, Schick 1965, Sauer 1972, Dondale and Redner 1975, Abraham 1996, Zeiders et al. 1999, Wade and Roughley 2010, Levi and Patrick 2013, Ovtcharenko et al. 2014, GBIF.org 2018c), it was possible to perform species distribution modelling (see methods for details).

Min Elevation/Depth (m): 0

Max Elevation/Depth (m): 3810

Range description: This species is known from several sites and is relatively well-recorded in the USA and Canada (Thorell 1877, Banks 1892, Emerton 1894, Gertsch 1939, Kaston 1948, Schick 1965, Sauer 1972, Dondale and Redner 1975, Abraham 1996, Zeiders et al. 1999, Wade and Roughley 2010, Levi and Patrick 2013, Ovtcharenko et al. 2014, GBIF.org 2018c). It is predicted to be present throughout USA and southernmost parts of Canada.

#### New occurrences

#### Extent of occurrence

EOO (km2): 11568241

Trend: Stable

Justification for trend: As it is a widespread species with no known threats, the trend is assumed to be stable.

Causes ceased?: Yes

Causes understood?: Yes

Causes reversible?: Yes

Extreme fluctuations?: No

#### Area of occupancy

Trend: Stable

Justification for trend: As it is a widespread species with no known threats, the trend is assumed to be stable.

Causes ceased?: Yes

Causes understood?: Yes

Causes reversible?: Yes

Extreme fluctuations?: No

AOO (km2): 10103920

#### Locations

Number of locations: Not applicable

Justification for number of locations: No known threats to the species.

Trend: Unknown

Extreme fluctuations?: Unknown

#### Population

Number of individuals: Unknown

Trend: Stable

Justification for trend: As it is a widespread species with no known threats, the trend is assumed to be stable.

Causes ceased?: Yes

Causes understood?: Yes

Causes reversible?: Yes

Extreme fluctuations?: No

Population Information (Narrative): This species is relatively well-recorded and widespread in the USA and in the southermost parts of Canada, which indicates a stable population trend.

#### Subpopulations

Trend: Stable

Justification for trend: As it is a widespread species with no known threats, the trend is assumed to be stable.

Extreme fluctuations?: No

Severe fragmentation?: No

#### Habitat

System: Terrestrial

Habitat specialist: Unknown

Habitat (narrative): This species seems to adapt well to different kinds of habitats. Specimens have been found from coniferous forests (Ovtcharenko et al. 2014), peat bogs, under leaves in a swamp, under stones and under bark and logs and dead leaves (Kaston 1948), from tallgrass prairie and grasslands (Abraham 1996, Wade and Roughley 2010). Dondale and Redner (1975) found specimens from "a field edge in Alberta, from talus at 10,000 ft elevation and from Juniper-Douglas fir forest in Colorado, from the nest of a house sparrow in North Dakota, and from pine litter in Wisconsin".

Trend in extent, area or quality?: Stable

Justification for trend: This species has been reported in various habitats, indicating it can adapt relatively well in different environments.

##### Habitat

Habitat importance: Major Importance

Habitats: 1.4. Forest - Temperate4.4. Grassland - Temperate5.4. Wetlands (inland) - Bogs, Marshes, Swamps, Fens, Peatlands

#### Habitat

Habitat importance: Major Importance

Habitats: 1.4. Forest - Temperate4.4. Grassland - Temperate5.4. Wetlands (inland) - Bogs, Marshes, Swamps, Fens, Peatlands

#### Ecology

Size: 3-4.3 mm

Generation length (yr): 1

Dependency of single sp?: Unknown

Ecology and traits (narrative): Mature individuals occur from March to November (Kaston 1948). Thomisids in general are ambush predators and do not build webs. Also known as crab spiders, they are most active during the day and usually wear a cryptic colour which help them to camouflage and wait for their prey, for example, by sitting on a plant. With their acute vision, they detect the prey and then attack. The prey are sometimes over twice the size of the spider and are paralysed with strong venom (Dippenaar-Schoeman and Jocqué 1997). Species of this genus are dark in colour and inhabit the leaf litter, bark of trees and open areas (Dippenaar-Schoeman and Jocqué 1997, Paquin et al. 2008).

#### Threats

Justification for threats: No known threats.

##### Threats

Threat type: Past

Threats: 12. Other options - Other threat

#### Threats

Threat type: Past

Threats: 12. Other options - Other threat

#### Conservation

Justification for conservation actions: There are several protected areas within the range of this species (United Nations Environment World Conservation Monitoring Centre 2017).

##### Conservation actions

Conservation action type: In Place

Conservation actions: 1.1. Land/water protection - Site/area protection1.2. Land/water protection - Resource & habitat protection

#### Conservation actions

Conservation action type: In Place

Conservation actions: 1.1. Land/water protection - Site/area protection1.2. Land/water protection - Resource & habitat protection

#### Other

##### Use and trade

Use type: International

Use and trade: 18. Unknown

##### Ecosystem services

Ecosystem service type: Very important

##### Research needed

Research needed: 3.1. Monitoring - Population trends3.4. Monitoring - Habitat trends

Justification for research needed: Monitoring is needed to confirm current population and habitat trends.

#### Use and trade

Use type: International

Use and trade: 18. Unknown

#### Ecosystem services

Ecosystem service type: Very important

#### Research needed

Research needed: 3.1. Monitoring - Population trends3.4. Monitoring - Habitat trends

Justification for research needed: Monitoring is needed to confirm current population and habitat trends.

#### Viability analysis

### Ozyptila hardyi

#### Species information

Scientific name: Ozyptila hardyi

Species authority: Gertsch, 1953

Kingdom: Animalia

Phylum: Arthropoda

Class: Arachnida

Order: Araneae

Family: Thomisidae

Region for assessment: Global

#### Geographic range

Biogeographic realm: Nearctic

Countries: United States

Map of records (Google Earth): Suppl. material 38

Basis of EOO and AOO: Unknown

Basis (narrative): Unknown EOO or AOO.

Min Elevation/Depth (m): 0

Max Elevation/Depth (m): 0

Range description: Known only from the type locality in Laguna Madre, Texas, USA, recorded in 1945 (Gertsch 1953).

#### New occurrences

#### Extent of occurrence

EOO (km2): Unknown

Trend: Unknown

Causes ceased?: Unknown

Causes understood?: Unknown

Causes reversible?: Unknown

Extreme fluctuations?: Unknown

#### Area of occupancy

Trend: Unknown

Causes ceased?: Unknown

Causes understood?: Unknown

Causes reversible?: Unknown

Extreme fluctuations?: Unknown

AOO (km2): Unknown

#### Locations

Number of locations: Unknown

Trend: Unknown

Extreme fluctuations?: No

#### Population

Number of individuals: Unknown

Trend: Unknown

Causes ceased?: Unknown

Causes understood?: Unknown

Causes reversible?: Unknown

Extreme fluctuations?: Unknown

Population Information (Narrative): No population size estimates exist.

#### Subpopulations

Trend: Unknown

Extreme fluctuations?: Unknown

Severe fragmentation?: Unknown

#### Habitat

System: Terrestrial

Habitat specialist: No

Habitat (narrative): A single specimen was found from the nest of a Southern Plains Woodrat (*Neotoma micropus*, Gertsch 1953). Otherwise, the habitat requirements of this particular species remain unknown. *Ozyptila* tend to live on the ground, amongst leaf litter or on the bark of trees. The locality seems to fall into the ecoregion of desert and xeric shrublands (Olson et al. 2001).

Trend in extent, area or quality?: Unknown

##### Habitat

Habitat importance: Major Importance

Habitats: 18. Unknown

#### Habitat

Habitat importance: Major Importance

Habitats: 18. Unknown

#### Ecology

Size: 2.2 mm

Generation length (yr): 1

Dependency of single sp?: Unknown

Ecology and traits (narrative): Thomisids in general are ambush predators and do not build webs. Also known as crab spiders, they are most active during the day and usually wear a cryptic colour which help them to camouflage and wait for their prey, for example, by sitting on a plant. With their acute vision, they detect the prey and then attack. The prey are sometimes over twice the size of the spider and are paralysed with strong venom (Dippenaar-Schoeman and Jocqué 1997). Species of this genus are dark in colour and inhabit the leaf litter, bark of trees and open areas (Dippenaar-Schoeman and Jocqué 1997, Paquin et al. 2008).

#### Threats

Justification for threats: Unknown threats.

##### Threats

Threat type: Past

Threats: 12. Other options - Other threat

#### Threats

Threat type: Past

Threats: 12. Other options - Other threat

#### Conservation

##### Conservation actions

#### Conservation actions

#### Other

##### Use and trade

Use type: International

Use and trade: 18. Unknown

##### Ecosystem services

Ecosystem service type: Very important

##### Research needed

Research needed: 1.2. Research - Population size, distribution & trends1.3. Research - Life history & ecology1.5. Research - Threats

Justification for research needed: Basic research is needed to know the current distribution and population size and trends, ecology and traits of the species, along with possible threats.

#### Use and trade

Use type: International

Use and trade: 18. Unknown

#### Ecosystem services

Ecosystem service type: Very important

#### Research needed

Research needed: 1.2. Research - Population size, distribution & trends1.3. Research - Life history & ecology1.5. Research - Threats

Justification for research needed: Basic research is needed to know the current distribution and population size and trends, ecology and traits of the species, along with possible threats.

#### Viability analysis

### Stephanopis yulensis

#### Species information

Scientific name: Stephanopis yulensis

Species authority: Thorell, 1881

Kingdom: Animalia

Phylum: Arthropoda

Class: Arachnida

Order: Araneae

Family: Thomisidae

Region for assessment: Global

#### Geographic range

Biogeographic realm: Australasian

Countries: Papua New Guinea

Map of records (Google Earth): Suppl. material 39

Basis of EOO and AOO: Unknown

Basis (narrative): Unknown EOO or AOO.

Min Elevation/Depth (m): 60

Max Elevation/Depth (m): 60

Range description: Known only from the type locality in Roro (Yule Island). The species is only mentioned in its original taxonomical description (Thorell 1881) and has not been recorded for over 135 years. But despite the long period with no observations and the reduced size of the island where the species was found (13 km2), it is only 2 km away from the mainland at its nearest point and very few arachnological surveys have ever been conducted in the country, none in the region close to Yule Island. Therefore the fact that it has not been found for so long can be easily due to the lack of prospecting.

#### New occurrences

#### Extent of occurrence

EOO (km2): Unknown

Trend: Unknown

Causes ceased?: Unknown

Causes understood?: Unknown

Causes reversible?: Unknown

Extreme fluctuations?: Unknown

#### Area of occupancy

Trend: Unknown

Causes ceased?: Unknown

Causes understood?: Unknown

Causes reversible?: Unknown

Extreme fluctuations?: Unknown

AOO (km2): Unknown

#### Locations

Number of locations: Unknown

Trend: Unknown

Extreme fluctuations?: Unknown

#### Population

Number of individuals: Unknown

Trend: Unknown

Causes ceased?: Unknown

Causes understood?: Unknown

Causes reversible?: Unknown

Extreme fluctuations?: Unknown

Population Information (Narrative): No population size estimates exist.

#### Subpopulations

Trend: Unknown

Extreme fluctuations?: Unknown

Severe fragmentation?: Unknown

#### Habitat

System: Terrestrial

Habitat specialist: Unknown

Habitat (narrative): No habitat data was recorded, however, the region of Yule Island is dominated by moist broadleaf forest (Olson et al. 2001).

Trend in extent, area or quality?: Unknown

##### Habitat

Habitat importance: Major Importance

Habitats: 18. Unknown

#### Habitat

Habitat importance: Major Importance

Habitats: 18. Unknown

#### Ecology

Size: 9.5 mm

Generation length (yr): 1

Dependency of single sp?: Unknown

Ecology and traits (narrative): Thomisids in general are ambush predators and do not build webs. Also known as crab spiders, they are most active during the day and usually wear a cryptic colour which help them to camouflage and wait for their prey, for example, by sitting on a plant. With their acute vision, they detect the prey and then attack. The prey are sometimes over twice the size of the spider and are paralysed with strong venom (Dippenaar-Schoeman and Jocqué 1997).

#### Threats

Justification for threats: Unknown threats.

##### Threats

Threat type: Past

Threats: 12. Other options - Other threat

#### Threats

Threat type: Past

Threats: 12. Other options - Other threat

#### Conservation

##### Conservation actions

#### Conservation actions

#### Other

##### Use and trade

Use type: International

##### Ecosystem services

Ecosystem service type: Very important

##### Research needed

Research needed: 1.2. Research - Population size, distribution & trends1.3. Research - Life history & ecology1.5. Research - Threats

Justification for research needed: Basic research is needed to know the current distribution and population size and trends, ecology and traits of the species, along with possible threats.

#### Use and trade

Use type: International

#### Ecosystem services

Ecosystem service type: Very important

#### Research needed

Research needed: 1.2. Research - Population size, distribution & trends1.3. Research - Life history & ecology1.5. Research - Threats

Justification for research needed: Basic research is needed to know the current distribution and population size and trends, ecology and traits of the species, along with possible threats.

#### Viability analysis

### Synema adjunctum

#### Species information

Scientific name: Synema adjunctum

Species authority: O. Pickard-Cambridge, 1891

Kingdom: Animalia

Phylum: Arthropoda

Class: Arachnida

Order: Araneae

Family: Thomisidae

Region for assessment: Global

#### Geographic range

Biogeographic realm: Neotropical

Countries: Panama

Map of records (Google Earth): Suppl. material 40

Basis of EOO and AOO: Unknown

Basis (narrative): Unknown EOO or AOO.

Min Elevation/Depth (m): 140

Max Elevation/Depth (m): 1170

Range description: Known only from the type locality, Volcan de Chiriqui, in Panama, prior to 1891 (Pickard-Cambridge 1891, Pickard-Cambridge 1900).

#### New occurrences

#### Extent of occurrence

EOO (km2): Unknown

Trend: Unknown

Causes ceased?: Unknown

Causes understood?: Unknown

Causes reversible?: Unknown

Extreme fluctuations?: Unknown

#### Area of occupancy

Trend: Unknown

Causes ceased?: Unknown

Causes understood?: Unknown

Causes reversible?: Unknown

Extreme fluctuations?: Unknown

AOO (km2): Unknown

#### Locations

Number of locations: Unknown

Trend: Unknown

Extreme fluctuations?: Unknown

#### Population

Number of individuals: Unknown

Trend: Unknown

Causes ceased?: Unknown

Causes understood?: Unknown

Causes reversible?: Unknown

Extreme fluctuations?: Unknown

Population Information (Narrative): No population size estimates exist.

#### Subpopulations

Trend: Unknown

Extreme fluctuations?: Unknown

Severe fragmentation?: Unknown

#### Habitat

System: Terrestrial

Habitat specialist: Unknown

Habitat (narrative): The type locality of this species is in the region of tropical and subtropical moist broadleaf forests (Olson et al. 2001). Otherwise the preferred habitat is unknown.

Trend in extent, area or quality?: Unknown

##### Habitat

Habitat importance: Major Importance

Habitats: 18. Unknown

#### Habitat

Habitat importance: Major Importance

Habitats: 18. Unknown

#### Ecology

Size: <3 mm

Generation length (yr): 1

Dependency of single sp?: Unknown

Ecology and traits (narrative): Thomisids in general are ambush predators and do not build webs. Also known as crab spiders, they are most active during the day and usually wear a cryptic colour which help them to camouflage and wait for their prey, for example, by sitting on a plant. With their acute vision, they detect the prey and then attack. The prey are sometimes over twice the size of the spider and are paralysed with strong venom (Dippenaar-Schoeman and Jocqué 1997).

#### Threats

Justification for threats: Unknown threats.

##### Threats

Threat type: Past

Threats: 12. Other options - Other threat

#### Threats

Threat type: Past

Threats: 12. Other options - Other threat

#### Conservation

Justification for conservation actions: The type locality is inside Volcan Baru National Park, which indicates that, at least in this area, the species could be preserved (United Nations Environment World Conservation Monitoring Centre 2017).

##### Conservation actions

Conservation action type: In Place

Conservation actions: 1.1. Land/water protection - Site/area protection1.2. Land/water protection - Resource & habitat protection

#### Conservation actions

Conservation action type: In Place

Conservation actions: 1.1. Land/water protection - Site/area protection1.2. Land/water protection - Resource & habitat protection

#### Other

##### Use and trade

Use type: International

Use and trade: 18. Unknown

##### Ecosystem services

Ecosystem service type: Very important

##### Research needed

Research needed: 1.2. Research - Population size, distribution & trends1.3. Research - Life history & ecology1.5. Research - Threats

Justification for research needed: Basic research is needed to know the current distribution and population size and trends, ecology and traits of the species, along with possible threats.

#### Use and trade

Use type: International

Use and trade: 18. Unknown

#### Ecosystem services

Ecosystem service type: Very important

#### Research needed

Research needed: 1.2. Research - Population size, distribution & trends1.3. Research - Life history & ecology1.5. Research - Threats

Justification for research needed: Basic research is needed to know the current distribution and population size and trends, ecology and traits of the species, along with possible threats.

#### Viability analysis

### Synema hildebrandti

#### Species information

Scientific name: Synema hildebrandti

Species authority: Dahl, 1907

Kingdom: Animalia

Phylum: Arthropoda

Class: Arachnida

Order: Araneae

Family: Thomisidae

Region for assessment: Global

#### Geographic range

Biogeographic realm: Afrotropical

Countries: Madagascar

Map of records (Google Earth): Suppl. material 41

Basis of EOO and AOO: Unknown

Basis (narrative): Unknown EOO or AOO.

Min Elevation/Depth (m): 1720

Max Elevation/Depth (m): 1720

Range description: Known only from Madagascar, no locality specified, recorded prior to 1905 (Dahl 1905).

#### New occurrences

#### Extent of occurrence

EOO (km2): Unknown

Trend: Unknown

Causes ceased?: Unknown

Causes understood?: Unknown

Causes reversible?: Unknown

Extreme fluctuations?: Unknown

#### Area of occupancy

Trend: Unknown

Causes ceased?: Unknown

Causes understood?: Unknown

Causes reversible?: Unknown

Extreme fluctuations?: Unknown

AOO (km2): Unknown

#### Locations

Number of locations: Unknown

Trend: Unknown

Extreme fluctuations?: No

#### Population

Number of individuals: Unknown

Trend: Unknown

Causes ceased?: Unknown

Causes understood?: Unknown

Causes reversible?: Unknown

Extreme fluctuations?: Unknown

Population Information (Narrative): Population size and trend are unknown.

#### Subpopulations

Trend: Unknown

Extreme fluctuations?: Unknown

Severe fragmentation?: Unknown

#### Habitat

System: Terrestrial

Habitat specialist: Unknown

Habitat (narrative): Madagascar is mostly covered with tropical and subtropical moist and dry broadleaf forests but also deserts and xeric shrublands in the southern part (Olson et al. 2001). However, since the type locality of this species is uncertain, the preferred habitat remains unknown.

Trend in extent, area or quality?: Unknown

##### Habitat

Habitat importance: Major Importance

Habitats: 18. Unknown

#### Habitat

Habitat importance: Major Importance

Habitats: 18. Unknown

#### Ecology

Size: Unknown

Generation length (yr): 1

Dependency of single sp?: Unknown

Ecology and traits (narrative): Thomisids in general are ambush predators and do not build webs. Also known as crab spiders, they are most active during the day and usually wear a cryptic colour which help them to camouflage and wait for their prey, for example, by sitting on a plant. With their acute vision, they detect the prey and then attack. The prey are sometimes over twice the size of the spider and are paralysed with strong venom (Dippenaar-Schoeman and Jocqué 1997).

#### Threats

Justification for threats: Unknown threats.

##### Threats

Threat type: Past

Threats: 12. Other options - Other threat

#### Threats

Threat type: Past

Threats: 12. Other options - Other threat

#### Conservation

##### Conservation actions

#### Conservation actions

#### Other

##### Use and trade

Use type: International

Use and trade: 18. Unknown

##### Ecosystem services

Ecosystem service type: Very important

##### Research needed

Research needed: 1.2. Research - Population size, distribution & trends1.3. Research - Life history & ecology1.5. Research - Threats

Justification for research needed: Basic research is needed to know the current distribution and population size and trends, ecology and traits of the species, along with possible threats.

#### Use and trade

Use type: International

Use and trade: 18. Unknown

#### Ecosystem services

Ecosystem service type: Very important

#### Research needed

Research needed: 1.2. Research - Population size, distribution & trends1.3. Research - Life history & ecology1.5. Research - Threats

Justification for research needed: Basic research is needed to know the current distribution and population size and trends, ecology and traits of the species, along with possible threats.

#### Viability analysis

### Thomisus litoris

#### Species information

Scientific name: Thomisus litoris

Species authority: Strand, 1913

Kingdom: Animalia

Phylum: Arthropoda

Class: Arachnida

Order: Araneae

Family: Thomisidae

Region for assessment: Global

#### Geographic range

Biogeographic realm: Afrotropical

Countries: Rwanda

Map of records (Google Earth): Suppl. material 42

Basis of EOO and AOO: Unknown

Basis (narrative): Unknown EOO or AOO.

Min Elevation/Depth (m): 1430

Max Elevation/Depth (m): 1430

Range description: Known only from the type locality in Central Africa, specifically at Kiwu Lake in Rwanda, recorded in 1907 (Strand 1913). No records have been published after species description, which may be due to insufficient exploration.

#### New occurrences

#### Extent of occurrence

EOO (km2): Unknown

Trend: Unknown

Causes ceased?: Unknown

Causes understood?: Unknown

Causes reversible?: Unknown

Extreme fluctuations?: Unknown

#### Area of occupancy

Trend: Unknown

Causes ceased?: Unknown

Causes understood?: Unknown

Causes reversible?: Unknown

Extreme fluctuations?: Unknown

AOO (km2): Unknown

#### Locations

Number of locations: Unknown

Trend: Unknown

Extreme fluctuations?: Unknown

#### Population

Number of individuals: Unknown

Trend: Unknown

Causes ceased?: Unknown

Causes understood?: Unknown

Causes reversible?: Unknown

Extreme fluctuations?: Unknown

Population Information (Narrative): No population size estimates exist.

#### Subpopulations

Trend: Unknown

Extreme fluctuations?: Unknown

Severe fragmentation?: Unknown

#### Habitat

System: Terrestrial

Habitat specialist: Unknown

Habitat (narrative): Only Lake Kiwu is mentioned in the original description (Strand 1913), which is located in the tropical and subtropical moist broadleaf forest ecoregion (Olson et al. 2001). Otherwise the preferred habitat of the species is unknown.

Trend in extent, area or quality?: Unknown

##### Habitat

Habitat importance: Major Importance

Habitats: 18. Unknown

#### Habitat

Habitat importance: Major Importance

Habitats: 18. Unknown

#### Ecology

Size: 5 mm

Generation length (yr): 1

Dependency of single sp?: Unknown

Ecology and traits (narrative): Thomisids in general are ambush predators and do not build webs. Also known as crab spiders, they are most active during the day and usually wear a cryptic colour which help them to camouflage and wait for their prey, for example, by sitting on a plant. With their acute vision, they detect the prey and then attack. The prey are sometimes over twice the size of the spider and are paralysed with strong venom (Dippenaar-Schoeman and Jocqué 1997).

#### Threats

Justification for threats: Unknown threats.

##### Threats

Threat type: Past

Threats: 12. Other options - Other threat

#### Threats

Threat type: Past

Threats: 12. Other options - Other threat

#### Conservation

##### Conservation actions

#### Conservation actions

#### Other

##### Use and trade

Use type: International

Use and trade: 18. Unknown

##### Ecosystem services

Ecosystem service type: Very important

##### Research needed

Research needed: 1.2. Research - Population size, distribution & trends1.3. Research - Life history & ecology1.5. Research - Threats

Justification for research needed: Basic research is needed to know the current distribution and population size and trends, ecology and traits of the species, along with possible threats.

#### Use and trade

Use type: International

Use and trade: 18. Unknown

#### Ecosystem services

Ecosystem service type: Very important

#### Research needed

Research needed: 1.2. Research - Population size, distribution & trends1.3. Research - Life history & ecology1.5. Research - Threats

Justification for research needed: Basic research is needed to know the current distribution and population size and trends, ecology and traits of the species, along with possible threats.

#### Viability analysis

### Tmarus peruvianus

#### Species information

Scientific name: Tmarus peruvianus

Species authority: Berland, 1913

Kingdom: Animalia

Phylum: Arthropoda

Class: Arachnida

Order: Araneae

Family: Thomisidae

Region for assessment: Global

#### Geographic range

Biogeographic realm: Neotropical

Countries: Peru

Map of records (Google Earth): Suppl. material 43

Basis of EOO and AOO: Unknown

Basis (narrative): Unknown EOO or AOO.

Min Elevation/Depth (m): 180

Max Elevation/Depth (m): 180

Range description: Known only from the type locality in North Peru, recorded prior to 1913 (Berland 1913).

#### New occurrences

#### Extent of occurrence

EOO (km2): Unknown

Trend: Unknown

Causes ceased?: Unknown

Causes understood?: Unknown

Causes reversible?: Unknown

Extreme fluctuations?: Unknown

#### Area of occupancy

Trend: Unknown

Causes ceased?: Unknown

Causes understood?: Unknown

Causes reversible?: Unknown

Extreme fluctuations?: Unknown

AOO (km2): Unknown

#### Locations

Number of locations: Unknown

Trend: Unknown

Extreme fluctuations?: Unknown

#### Population

Number of individuals: Unknown

Trend: Unknown

Causes ceased?: Unknown

Causes understood?: Unknown

Causes reversible?: Unknown

Extreme fluctuations?: Unknown

Population Information (Narrative): No population size estimates exist.

#### Subpopulations

Trend: Unknown

Extreme fluctuations?: Unknown

Severe fragmentation?: Unknown

#### Habitat

System: Terrestrial

Habitat specialist: Unknown

Habitat (narrative): Known from desertic habitats in North Peru (Berland 1913).

Trend in extent, area or quality?: Unknown

##### Habitat

Habitat importance: Major Importance

Habitats: 8.1. Desert - Hot8.2. Desert - Temperate

#### Habitat

Habitat importance: Major Importance

Habitats: 8.1. Desert - Hot8.2. Desert - Temperate

#### Ecology

Size: 3 mm

Generation length (yr): 1

Dependency of single sp?: Unknown

Ecology and traits (narrative): Thomisids in general are ambush predators and do not build webs. Also known as crab spiders, they are most active during the day and usually wear a cryptic colour which help them to camouflage and wait for their prey, for example, by sitting on a plant. With their acute vision, they detect the prey and then attack. The prey are sometimes over twice the size of the spider and are paralysed with strong venom (Dippenaar-Schoeman and Jocqué 1997).

#### Threats

Justification for threats: Unknown threats.

##### Threats

Threat type: Past

Threats: 12. Other options - Other threat

#### Threats

Threat type: Past

Threats: 12. Other options - Other threat

#### Conservation

##### Conservation actions

#### Conservation actions

#### Other

##### Use and trade

Use type: International

Use and trade: 18. Unknown

##### Ecosystem services

Ecosystem service type: Very important

##### Research needed

Research needed: 1.2. Research - Population size, distribution & trends1.3. Research - Life history & ecology1.5. Research - Threats

Justification for research needed: Basic research is needed to know the current distribution and population size and trends, ecology and traits of the species, along with possible threats.

#### Use and trade

Use type: International

Use and trade: 18. Unknown

#### Ecosystem services

Ecosystem service type: Very important

#### Research needed

Research needed: 1.2. Research - Population size, distribution & trends1.3. Research - Life history & ecology1.5. Research - Threats

Justification for research needed: Basic research is needed to know the current distribution and population size and trends, ecology and traits of the species, along with possible threats.

#### Viability analysis

### Xysticus kalandadzei

#### Species information

Scientific name: Xysticus kalandadzei

Species authority: Mcheidze & Utochkin, 1971

Kingdom: Araneae

Phylum: Arthropoda

Class: Arachnida

Order: Araneae

Family: Thomisidae

Region for assessment: Global

#### Geographic range

Biogeographic realm: Palearctic

Countries: Georgia

Map of records (Google Earth): Suppl. material 44

Basis of EOO and AOO: Unknown

Basis (narrative): Unknown EOO or AOO.

Min Elevation/Depth (m): 510

Max Elevation/Depth (m): 1180

Range description: This species is known from Georgia, specifically from Kiketi, Betania, recorded in 1962 (Mcheidze and Utochkin 1971) and from Tbilisi recorded prior to 2006 (Pkhakadze 2006). Suggested to be endemic to Georgia (Mcheidze 2014), although the precise distribution is unknown given the low number of records.

#### New occurrences

#### Extent of occurrence

EOO (km2): Unknown

Trend: Unknown

Causes ceased?: Unknown

Causes understood?: Unknown

Causes reversible?: Unknown

Extreme fluctuations?: Unknown

#### Area of occupancy

Trend: Unknown

Causes ceased?: Unknown

Causes understood?: Unknown

Causes reversible?: Unknown

Extreme fluctuations?: Unknown

AOO (km2): Unknown

#### Locations

Number of locations: Unknown

Trend: Unknown

Extreme fluctuations?: Unknown

#### Population

Number of individuals: Unknown

Trend: Unknown

Causes ceased?: Unknown

Causes understood?: Unknown

Causes reversible?: Unknown

Extreme fluctuations?: Unknown

Population Information (Narrative): No population size estimates exist.

#### Subpopulations

Trend: Unknown

Extreme fluctuations?: Unknown

Severe fragmentation?: Unknown

#### Habitat

System: Terrestrial

Habitat specialist: Unknown

Habitat (narrative): All specimens were found in grasslands (Mcheidze and Utochkin 1971).

Trend in extent, area or quality?: Unknown

##### Habitat

Habitat importance: Major Importance

Habitats: 4.4. Grassland - Temperate

#### Habitat

Habitat importance: Major Importance

Habitats: 4.4. Grassland - Temperate

#### Ecology

Size: 3.6 mm

Generation length (yr): 11

Dependency of single sp?: Unknown

Ecology and traits (narrative): Thomisids in general are ambush predators and do not build webs. Also known as crab spiders, they are most active during the day and usually wear a cryptic colour which help them to camouflage and wait for their prey, for example, by sitting on a plant. With their acute vision, they detect the prey and then attack. The prey are sometimes over twice the size of the spider and are paralysed with strong venom (Dippenaar-Schoeman and Jocqué 1997). Species of *Xysticus* are relatively dark in colour and instead of flowers they hunt on the leaf litter, bark of trees and open areas (Dippenaar-Schoeman and Jocqué 1997, Paquin et al. 2008).

#### Threats

Justification for threats: Unknown threats.

##### Threats

Threat type: Past

Threats: 12. Other options - Other threat

#### Threats

Threat type: Past

Threats: 12. Other options - Other threat

#### Conservation

##### Conservation actions

#### Conservation actions

#### Other

##### Use and trade

Use type: International

Use and trade: 18. Unknown

##### Ecosystem services

Ecosystem service type: Very important

##### Research needed

Research needed: 1.2. Research - Population size, distribution & trends1.3. Research - Life history & ecology1.5. Research - Threats

Justification for research needed: Basic research is needed to know the current distribution and population size and trends, ecology and traits of the species, along with possible threats.

#### Use and trade

Use type: International

Use and trade: 18. Unknown

#### Ecosystem services

Ecosystem service type: Very important

#### Research needed

Research needed: 1.2. Research - Population size, distribution & trends1.3. Research - Life history & ecology1.5. Research - Threats

Justification for research needed: Basic research is needed to know the current distribution and population size and trends, ecology and traits of the species, along with possible threats.

#### Viability analysis

### Xysticus tristrami

#### Species information

Scientific name: Xysticus tristrami

Species authority: (O. Pickard-Cambridge, 1872)

Kingdom: Animalia

Phylum: Arthropoda

Class: Arachnida

Order: Araneae

Family: Thomisidae

Region for assessment: Global

#### Geographic range

Biogeographic realm: Palearctic

Countries: UzbekistanPalestinian Territory, OccupiedQatarSaudi ArabiaAfghanistanLebanonCyprusSyrian Arab RepublicTajikistanTurkmenistanIraqIran, Islamic Republic ofPakistanIsraelJordanKazakhstanKuwaitKyrgyzstanYemenGeorgiaTurkeyArmeniaAzerbaijanIndiaMacedonia, the former Yugoslav Republic ofAlbaniaBulgariaMontenegroLibyaSudanGreeceSerbiaEgyptRussian FederationUkraineChina

Map of records (Google Earth): Suppl. material 45

Basis of EOO and AOO: Species Distribution Model

Basis (narrative): Given the relatively high number of records (Pickard-Cambridge 1872, Roewer 1962, Levy 1976, Dippenaar-Schoeman 1989, Marusik and Logunov 1990, El-Hennawy 2006, Lecigne 2016, Kiany et al. 2017, GBIF.org 2018d), it was possible to perform species distribution modelling (see methods for details).

Min Elevation/Depth (m): 0

Max Elevation/Depth (m): 4650

Range description: This species is well-recorded (Pickard-Cambridge 1872b, Roewer 1962, Levy 1976, Dippenaar-Schoeman 1989, Marusik and Logunov 1990, El-Hennawy 2006, Lecigne 2016, GBIF.org 2018d, Kiany et al. 2017) and should have a wide distribution from South Eastern Europe and North East Africa to Central Asia.

#### New occurrences

#### Extent of occurrence

EOO (km2): 14889354

Trend: Stable

Justification for trend: As it is a widespread species with no known threats, the trend is assumed to be stable.

Causes ceased?: Yes

Causes understood?: Yes

Causes reversible?: Yes

Extreme fluctuations?: No

#### Area of occupancy

Trend: Stable

Justification for trend: As it is a widespread species with no known threats, the trend is assumed to be stable.

Causes ceased?: Yes

Causes understood?: Yes

Causes reversible?: Yes

Extreme fluctuations?: No

AOO (km2): 11231076

#### Locations

Number of locations: Not applicable

Justification for number of locations: No known threats to the species.

Trend: Stable

Extreme fluctuations?: No

#### Population

Number of individuals: Unknown

Trend: Stable

Causes ceased?: Yes

Causes understood?: Yes

Causes reversible?: Yes

Extreme fluctuations?: No

Population Information (Narrative): No population size estimates exist. However, as it is a relatively well-recorded and widespread species with no known threats, we assume the trend to be stable.

#### Subpopulations

Trend: Stable

Extreme fluctuations?: No

Severe fragmentation?: No

#### Habitat

System: Terrestrial

Habitat specialist: No

Habitat (narrative): Specimens were found in a variety of relatively open habitat types, from rocky areas to shrublands. They were mainly found under rocks and stones and males running on the ground and upon plants and shrubs (Pickard-Cambridge 1872).

Trend in extent, area or quality?: Stable

##### Habitat

Habitat importance: Major Importance

Habitats: 3.4. Shrubland - Temperate3.5. Shrubland - Subtropical/Tropical Dry4.4. Grassland - Temperate4.5. Grassland - Subtropical/Tropical Dry6. Rocky areas (e.g. inland cliffs, mountain peaks)

#### Habitat

Habitat importance: Major Importance

Habitats: 3.4. Shrubland - Temperate3.5. Shrubland - Subtropical/Tropical Dry4.4. Grassland - Temperate4.5. Grassland - Subtropical/Tropical Dry6. Rocky areas (e.g. inland cliffs, mountain peaks)

#### Ecology

Size: 2-3 mm (prosoma length)

Generation length (yr): 1

Dependency of single sp?: No

Ecology and traits (narrative): Thomisids in general are ambush predators and do not build webs. Also known as crab spiders, they are most active during the day and usually wear a cryptic colour which help them to camouflage and wait for their prey, for example, by sitting on a plant. With their acute vision, they detect the prey and then attack. The prey are sometimes over twice the size of the spider and are paralysed with strong venom (Dippenaar-Schoeman and Jocqué 1997). Species of *Xysticus* are relatively dark in colour and instead of flowers they hunt on the leaf litter, bark of trees and open areas (Dippenaar-Schoeman and Jocqué 1997, Paquin et al. 2008). According to Levy (1976), the males and females of this particular species are both present in April and May, females occurring until August. Several unmated females have been reported to lay unfertilised eggs when in the laboratory (Levy 1976).

#### Threats

Justification for threats: No known threats.

##### Threats

Threat type: Past

Threats: 12. Other options - Other threat

#### Threats

Threat type: Past

Threats: 12. Other options - Other threat

#### Conservation

Justification for conservation actions: There are several protected areas within the range of this species (United Nations Environment World Conservation Monitoring Centre 2017).

##### Conservation actions

Conservation action type: In Place

Conservation actions: 1.1. Land/water protection - Site/area protection1.2. Land/water protection - Resource & habitat protection

#### Conservation actions

Conservation action type: In Place

Conservation actions: 1.1. Land/water protection - Site/area protection1.2. Land/water protection - Resource & habitat protection

#### Other

##### Use and trade

Use type: International

Use and trade: 18. Unknown

##### Ecosystem services

Ecosystem service type: Very important

##### Research needed

Research needed: 3.1. Monitoring - Population trends3.4. Monitoring - Habitat trends

Justification for research needed: Monitoring is needed to confirm the current population and habitat trends.

#### Use and trade

Use type: International

Use and trade: 18. Unknown

#### Ecosystem services

Ecosystem service type: Very important

#### Research needed

Research needed: 3.1. Monitoring - Population trends3.4. Monitoring - Habitat trends

Justification for research needed: Monitoring is needed to confirm the current population and habitat trends.

#### Viability analysis

### Longrita rastellata

#### Species information

Scientific name: Longrita rastellata

Species authority: Platnick, 2002

Kingdom: Animalia

Phylum: Arthropoda

Class: Arachnida

Order: Araneae

Family: Trochanteriidae

Region for assessment: Global

#### Geographic range

Biogeographic realm: Australasian

Countries: Australia

Map of records (Google Earth): Suppl. material 46

Basis of EOO and AOO: Unknown

Basis (narrative): Unknown EOO or AOO.

Min Elevation/Depth (m): 80

Max Elevation/Depth (m): 670

Range description: This species is known only from northern Queensland and Western Australia, recorded in 1985 and 1993, respectively (Platnick 2002). According to Platnick (2002), the species might be widespread in northern Australia.

#### New occurrences

#### Extent of occurrence

EOO (km2): Unknown

Trend: Unknown

Causes ceased?: Unknown

Causes understood?: Unknown

Causes reversible?: Unknown

Extreme fluctuations?: Unknown

#### Area of occupancy

Trend: Unknown

Causes ceased?: Unknown

Causes understood?: Unknown

Causes reversible?: Unknown

Extreme fluctuations?: Unknown

AOO (km2): Unknown

#### Locations

Number of locations: Unknown

Trend: Unknown

Extreme fluctuations?: No

#### Population

Number of individuals: Unknown

Trend: Unknown

Causes ceased?: Unknown

Causes understood?: Unknown

Causes reversible?: Unknown

Extreme fluctuations?: Unknown

Population Information (Narrative): No population size estimates exist. Platnick (2002) suggests this species may be quite common in the northern parts of Australia.

#### Subpopulations

Trend: Unknown

Extreme fluctuations?: Unknown

Severe fragmentation?: Unknown

#### Habitat

System: Terrestrial

Habitat specialist: Unknown

Habitat (narrative): All specimens were found under rocks (Platnick 2002), otherwise the habitat of this species remains unknown.

Trend in extent, area or quality?: Unknown

##### Habitat

Habitat importance: Major Importance

Habitats: 18. Unknown

#### Habitat

Habitat importance: Major Importance

Habitats: 18. Unknown

#### Ecology

Size: 18-19 mm

Generation length (yr): 1

Dependency of single sp?: Unknown

Ecology and traits (narrative): Trochanteriids are commonly known as scorpion spiders. They are flat-bodied free-living wanderers that do not build a web at all. They tend to hide in rock cracks, under bark or stones (Dippenaar-Schoeman and Jocqué 1997, Jocqué and Dippenaar-Schoeman 2006).

#### Threats

Justification for threats: Unknown threats.

##### Threats

Threat type: Past

Threats: 12. Other options - Other threat

#### Threats

Threat type: Past

Threats: 12. Other options - Other threat

#### Conservation

##### Conservation actions

#### Conservation actions

#### Other

##### Use and trade

Use type: International

Use and trade: 18. Unknown

##### Ecosystem services

Ecosystem service type: Very important

##### Research needed

Research needed: 1.2. Research - Population size, distribution & trends1.3. Research - Life history & ecology1.5. Research - Threats

Justification for research needed: Basic research is needed to know the current distribution and population size and trends, ecology and traits of the species, along with possible threats.

#### Use and trade

Use type: International

Use and trade: 18. Unknown

#### Ecosystem services

Ecosystem service type: Very important

#### Research needed

Research needed: 1.2. Research - Population size, distribution & trends1.3. Research - Life history & ecology1.5. Research - Threats

Justification for research needed: Basic research is needed to know the current distribution and population size and trends, ecology and traits of the species, along with possible threats.

#### Viability analysis

### Cavasteron guttulatum

#### Species information

Scientific name: Cavasteron guttulatum

Species authority: Baehr & Jocqué, 2000

Kingdom: Animalia

Phylum: Arthropoda

Class: Arachnida

Order: Araneae

Family: Zodariidae

Region for assessment: Global

#### Geographic range

Biogeographic realm: Australasian

Countries: Australia

Map of records (Google Earth): Suppl. material 47

Basis of EOO and AOO: Unknown

Basis (narrative): Unknown EOO or AOO.

Min Elevation/Depth (m): 150

Max Elevation/Depth (m): 160

Range description: Recorded in 1984 from only three localities that are far apart in South Australia (Jocqué and Baehr 2001).

#### New occurrences

#### Extent of occurrence

EOO (km2): Unknown

Trend: Unknown

Causes ceased?: Unknown

Causes understood?: Unknown

Causes reversible?: Unknown

Extreme fluctuations?: Unknown

#### Area of occupancy

Trend: Unknown

Causes ceased?: Unknown

Causes understood?: Unknown

Causes reversible?: Unknown

Extreme fluctuations?: Unknown

AOO (km2): Unknown

#### Locations

Number of locations: Unknown

Trend: Unknown

Extreme fluctuations?: Unknown

#### Population

Number of individuals: Unknown

Trend: Unknown

Causes ceased?: Unknown

Causes understood?: Unknown

Causes reversible?: Unknown

Extreme fluctuations?: Unknown

Population Information (Narrative): No population size estimates exist.

#### Subpopulations

Trend: Unknown

Extreme fluctuations?: No

Severe fragmentation?: No

#### Habitat

System: Terrestrial

Habitat specialist: No

Habitat (narrative): Recorded from scrub and sand plains (Jocqué and Baehr 2001).

Trend in extent, area or quality?: Unknown

##### Habitat

Habitat importance: Major Importance

Habitats: 3.4. Shrubland - Temperate3.5. Shrubland - Subtropical/Tropical Dry

#### Habitat

Habitat importance: Major Importance

Habitats: 3.4. Shrubland - Temperate3.5. Shrubland - Subtropical/Tropical Dry

#### Ecology

Size: 3.10 mm

Generation length (yr): 1

Dependency of single sp?: Unknown

Ecology and traits (narrative): Zodariids are mostly ground-dwellers and wanderers that do not build a web. In general they prefer dry habitats where they often make burrows for shelter (Dippenaar-Schoeman and Jocqué 1997).

#### Threats

Justification for threats: Unknown threats.

##### Threats

Threat type: Past

Threats: 12. Other options - Other threat

#### Threats

Threat type: Past

Threats: 12. Other options - Other threat

#### Conservation

##### Conservation actions

Conservation action type: In Place

#### Conservation actions

Conservation action type: In Place

#### Other

##### Use and trade

Use type: International

##### Ecosystem services

Ecosystem service type: Very important

##### Research needed

Research needed: 1.2. Research - Population size, distribution & trends1.3. Research - Life history & ecology1.5. Research - Threats

Justification for research needed: Basic research is needed to know the current distribution and population size and trends, ecology and traits of the species, along with possible threats.

#### Use and trade

Use type: International

#### Ecosystem services

Ecosystem service type: Very important

#### Research needed

Research needed: 1.2. Research - Population size, distribution & trends1.3. Research - Life history & ecology1.5. Research - Threats

Justification for research needed: Basic research is needed to know the current distribution and population size and trends, ecology and traits of the species, along with possible threats.

#### Viability analysis

### Storena gujaratensis

#### Species information

Scientific name: Storena gujaratensis

Species authority: Tikader & Patel, 1975

Kingdom: Animalia

Phylum: Arthropoda

Class: Arachnida

Order: Araneae

Family: Zodariidae

Region for assessment: Global

#### Geographic range

Biogeographic realm: Indomalayan

Countries: India

Map of records (Google Earth): Suppl. material 48

Basis of EOO and AOO: Unknown

Basis (narrative): Unknown EOO or AOO.

Min Elevation/Depth (m): 40

Max Elevation/Depth (m): 40

Range description: Known only from the type locality in Gujarat, India, recorded in 1972 (Tikader and Patel 1975).

#### New occurrences

#### Extent of occurrence

EOO (km2): Unknown

Trend: Unknown

Causes ceased?: Unknown

Causes understood?: Unknown

Causes reversible?: Unknown

Extreme fluctuations?: Unknown

#### Area of occupancy

Trend: Unknown

Causes ceased?: Unknown

Causes understood?: Unknown

Causes reversible?: Unknown

Extreme fluctuations?: Unknown

AOO (km2): Unknown

#### Locations

Number of locations: Unknown

Trend: Unknown

Extreme fluctuations?: Unknown

#### Population

Number of individuals: Unknown

Trend: Unknown

Causes ceased?: Unknown

Causes understood?: Unknown

Causes reversible?: Unknown

Extreme fluctuations?: Unknown

Population Information (Narrative): No population size estimates exist.

#### Subpopulations

Trend: Unknown

Extreme fluctuations?: Unknown

Severe fragmentation?: Unknown

#### Habitat

System: Terrestrial

Habitat specialist: Unknown

Habitat (narrative): This species was found under stones or dead leaves on the ground (Tikader and Patel 1975). Gujarat, the region from where this species was recorded, belongs to the ecoregion of deserts and xeric shrublands (Olson et al. 2001). Otherwise, the specific habitat of this species is unknown.

Trend in extent, area or quality?: Unknown

##### Habitat

Habitat importance: Major Importance

Habitats: 18. Unknown

#### Habitat

Habitat importance: Major Importance

Habitats: 18. Unknown

#### Ecology

Size: 6.4 mm

Generation length (yr): 1

Dependency of single sp?: Unknown

Ecology and traits (narrative): All specimens were found under stones or dead leaves (Tikader and Patel 1975), which indicates this species is a ground-dweller. Zodariids in general are ground-dwellers and wanderers, not building a web. They mostly prefer dry habitats often making burrows for shelter (Dippenaar-Schoeman and Jocqué 1997).

#### Threats

Justification for threats: Unknown threats.

##### Threats

Threat type: Past

Threats: 12. Other options - Other threat

#### Threats

Threat type: Past

Threats: 12. Other options - Other threat

#### Conservation

##### Conservation actions

#### Conservation actions

#### Other

##### Use and trade

Use type: International

Use and trade: 18. Unknown

##### Ecosystem services

Ecosystem service type: Very important

##### Research needed

Research needed: 1.2. Research - Population size, distribution & trends1.3. Research - Life history & ecology1.5. Research - Threats

Justification for research needed: Basic research is needed to know the current distribution and population size and trends, ecology and traits of the species, along with possible threats.

#### Use and trade

Use type: International

Use and trade: 18. Unknown

#### Ecosystem services

Ecosystem service type: Very important

#### Research needed

Research needed: 1.2. Research - Population size, distribution & trends1.3. Research - Life history & ecology1.5. Research - Threats

Justification for research needed: Basic research is needed to know the current distribution and population size and trends, ecology and traits of the species, along with possible threats.

#### Viability analysis

### Zodarion sytchevskajae

#### Species information

Scientific name: Zodarion sytchevskajae

Species authority: (Nenilin & Fet, 1985)

Kingdom: Animalia

Phylum: Arthropoda

Class: Arachnida

Order: Araneae

Family: Zodariidae

Region for assessment: Global

#### Geographic range

Biogeographic realm: Palearctic

Countries: Turkmenistan

Map of records (Google Earth): Suppl. material 49

Basis of EOO and AOO: Unknown

Basis (narrative): Unknown EOO or AOO.

Min Elevation/Depth (m): 200

Max Elevation/Depth (m): 1090

Range description: This species is known from three sites in Turkmenistan, last recorded prior to 2001 (Nenilin and Fet 1985, Marusik and Koponen 2001).

#### New occurrences

#### Extent of occurrence

EOO (km2): Unknown

Trend: Unknown

Causes ceased?: Unknown

Causes understood?: Unknown

Causes reversible?: Unknown

Extreme fluctuations?: Unknown

#### Area of occupancy

Trend: Unknown

Causes ceased?: Unknown

Causes understood?: Unknown

Causes reversible?: Unknown

Extreme fluctuations?: Unknown

AOO (km2): Unknown

#### Locations

Number of locations: Unknown

Trend: Unknown

Extreme fluctuations?: Unknown

#### Population

Number of individuals: Unknown

Trend: Unknown

Causes ceased?: Unknown

Causes understood?: Unknown

Causes reversible?: Unknown

Extreme fluctuations?: Unknown

Population Information (Narrative): This species seems to be widely distributed based on the type series, yet it has subsequently not been collected.

#### Subpopulations

Trend: Unknown

Extreme fluctuations?: Unknown

Severe fragmentation?: Unknown

#### Habitat

System: Terrestrial

Habitat specialist: Unknown

Habitat (narrative): This species has been recorded from *termitaria* (Marusik and Koponen 2001, Nenilin and Fet 1985) yet it is not known whether it also occurs in other types of microhabitats. Turkmenistan is mostly covered with deserts and xeric shrublands (Olson et al. 2001).

Trend in extent, area or quality?: Unknown

##### Habitat

Habitat importance: Major Importance

Habitats: 7.2. Caves and Subterranean Habitats (non-aquatic) - Other Subterranean Habitats8.1. Desert - Hot

#### Habitat

Habitat importance: Major Importance

Habitats: 7.2. Caves and Subterranean Habitats (non-aquatic) - Other Subterranean Habitats8.1. Desert - Hot

#### Ecology

Size: <2 mm

Generation length (yr): 1

Dependency of single sp?: Unknown

Ecology and traits (narrative): Zodariids in general are ground-dwellers and wanderers, not building a web. They mostly prefer dry habitats and many *Zodarion* are specialised in hunting ants, building an igloo-like retreat (Dippenaar-Schoeman and Jocqué 1997). This particular species may be specialised in preying on termites, since all the records available have been made in *termitaria* (Marusik and Koponen 2001, Nenilin and Fet 1985).

#### Threats

Justification for threats: Unknown threats.

##### Threats

Threat type: Past

Threats: 12. Other options - Other threat

#### Threats

Threat type: Past

Threats: 12. Other options - Other threat

#### Conservation

##### Conservation actions

#### Conservation actions

#### Other

##### Use and trade

Use type: International

Use and trade: 18. Unknown

##### Ecosystem services

Ecosystem service type: Very important

##### Research needed

Research needed: 1.2. Research - Population size, distribution & trends1.3. Research - Life history & ecology1.5. Research - Threats

Justification for research needed: Basic research is needed to know the current distribution in more detail and population size and trends, ecology and traits of the species, along with possible threats.

#### Use and trade

Use type: International

Use and trade: 18. Unknown

#### Ecosystem services

Ecosystem service type: Very important

#### Research needed

Research needed: 1.2. Research - Population size, distribution & trends1.3. Research - Life history & ecology1.5. Research - Threats

Justification for research needed: Basic research is needed to know the current distribution in more detail and population size and trends, ecology and traits of the species, along with possible threats.

#### Viability analysis

### Huntia deepensis

#### Species information

Scientific name: Huntia deepensis

Species authority: Gray & Thompson, 2001

Kingdom: Animalia

Phylum: Arthropoda

Class: Arachnida

Order: Araneae

Family: Zoropsidae

Region for assessment: Global

#### Geographic range

Biogeographic realm: Australasian

Countries: Australia

Map of records (Google Earth): Suppl. material 50

Basis of EOO and AOO: Species Distribution Model

Basis (narrative): Given the relatively high number of records (Gray and Thompson 2001), it was possible to perform species distribution modelling (see methods for details).

Min Elevation/Depth (m): 0

Max Elevation/Depth (m): 250

Range description: This species has been recorded from Australia only, the latest dates from 1990 (Gray and Thompson 2001).

#### New occurrences

#### Extent of occurrence

EOO (km2): 2697

Trend: Decline (inferred)

Justification for trend: Inferred from possible habitat loss due to the increase of frequency of forest fires.

Causes ceased?: No

Causes understood?: Yes

Causes reversible?: Yes

Extreme fluctuations?: No

#### Area of occupancy

Trend: Decline (inferred)

Justification for trend: Inferred from possible habitat loss due to the increase of frequency of forest fires.

Causes ceased?: No

Causes understood?: Yes

Causes reversible?: Yes

Extreme fluctuations?: No

AOO (km2): 1788

#### Locations

Number of locations: Unknown

Trend: Unknown

Extreme fluctuations?: Unknown

#### Population

Trend: Decline (inferred)

Justification for trend: Inferred from possible habitat loss due to the increase of frequency of forest fires.

Causes ceased?: Yes

Causes understood?: Yes

Causes reversible?: Yes

Extreme fluctuations?: No

#### Subpopulations

Trend: Decline (inferred)

Justification for trend: Inferred from possible habitat loss due to the increase of frequency of forest fires.

Extreme fluctuations?: No

Severe fragmentation?: No

#### Habitat

System: Terrestrial

Habitat specialist: Unknown

Habitat (narrative): This cribbelate species of hunting spider is found in forest and woodland habitats in south-western Australia (Gray and Thompson 2001). Specimens were recorded from Kari and tingle woodland, eucalypt woodland and from jarrah, sheoak & karri forests (Gray and Thompson 2001).

Trend in extent, area or quality?: Decline (estimated)

Justification for trend: The area of available habitat is estimated to be declining due to increasing frequency of forest fires in the region.

##### Habitat

Habitat importance: Major Importance

Habitats: 1.5. Forest - Subtropical/Tropical Dry3.8. Shrubland - Mediterranean-type Shrubby Vegetation

#### Habitat

Habitat importance: Major Importance

Habitats: 1.5. Forest - Subtropical/Tropical Dry3.8. Shrubland - Mediterranean-type Shrubby Vegetation

#### Ecology

Size: 9.17-9.69 mm

Generation length (yr): 1

Dependency of single sp?: Unknown

Ecology and traits (narrative): Members of the family Zoropsidae are large, agile wandering spiders that resemble Lycosids or wolf spiders though they have a different eye arrangement. Zoropsids have been found amongst the leaf litter in rainforests and under stones in Australia (Jocqué and Dippenaar-Schoeman 2006). The ecology of *Huntia deepensis* is largely unknown.

#### Threats

Justification for threats: From 50,000 to over 150,000 fires have been reported between 2012 and 2017 for Western Australia (Global Forest Watch 2014). This may cause a possible threat to the survival of this species, although this is uncertain.

##### Threats

Threat type: Ongoing

Threats: 7.1.1. Natural system modifications - Fire & fire suppression - Increase in fire frequency/intensity

#### Threats

Threat type: Ongoing

Threats: 7.1.1. Natural system modifications - Fire & fire suppression - Increase in fire frequency/intensity

#### Conservation

Justification for conservation actions: At least part of the range of this species is within protected areas, namely D'Entrecasteaux and West Cape Howe National Parks in Western Australia (United Nations Environment World Conservation Monitoring Centre 2017).

##### Conservation actions

Conservation action type: In Place

Conservation actions: 1.1. Land/water protection - Site/area protection1.2. Land/water protection - Resource & habitat protection

#### Conservation actions

Conservation action type: In Place

Conservation actions: 1.1. Land/water protection - Site/area protection1.2. Land/water protection - Resource & habitat protection

#### Other

##### Use and trade

Use type: International

Use and trade: 18. Unknown

##### Ecosystem services

Ecosystem service type: Very important

##### Research needed

Research needed: 1.3. Research - Life history & ecology3.1. Monitoring - Population trends3.4. Monitoring - Habitat trends

Justification for research needed: Monitoring is needed to confirm the current population and habitat trends. Also more data on the ecology and traits of this species is required to assess its sensitivity to forest fires.

#### Use and trade

Use type: International

Use and trade: 18. Unknown

#### Ecosystem services

Ecosystem service type: Very important

#### Research needed

Research needed: 1.3. Research - Life history & ecology3.1. Monitoring - Population trends3.4. Monitoring - Habitat trends

Justification for research needed: Monitoring is needed to confirm the current population and habitat trends. Also more data on the ecology and traits of this species is required to assess its sensitivity to forest fires.

#### Viability analysis

## Results

**Figure 1.**
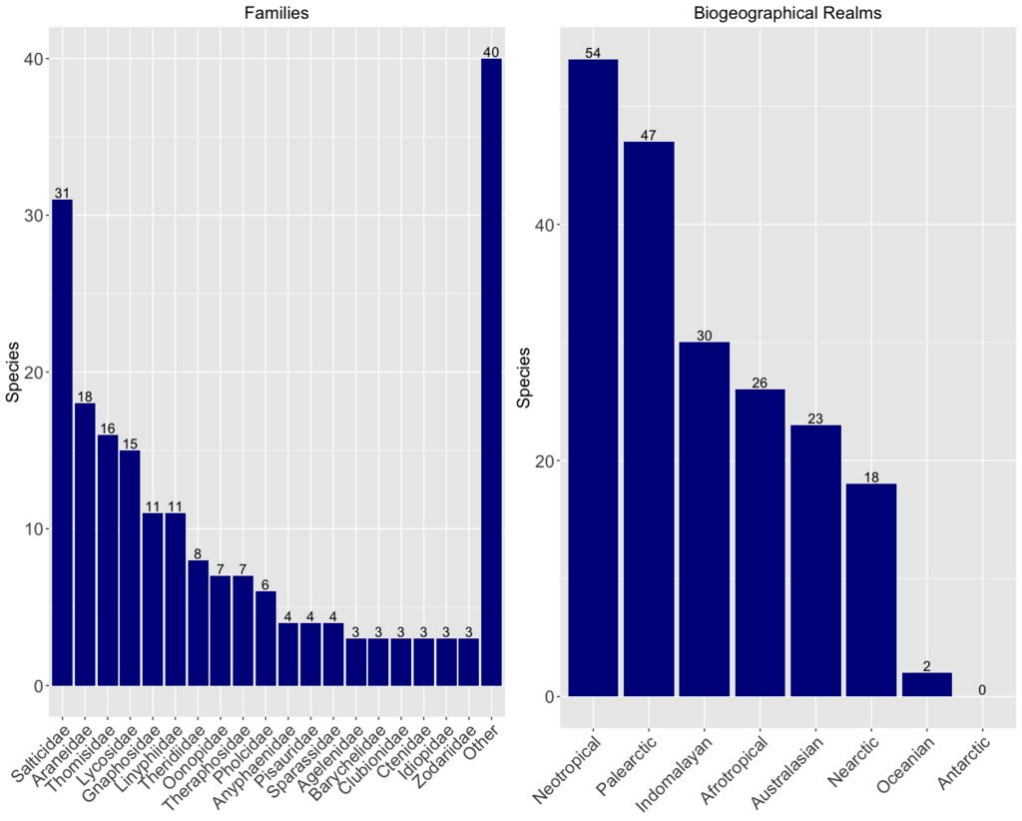
Family and biogeographical realm of the 200 assessed species.

A total of 200 species have been assessed within this project (Seppälä et al. 2018a, Seppälä et al. 2018b, Seppälä et al. 2018c, this work), belonging to 47 of the currently recognised 118 families (Fig. 1; World Spider Catalog 2018). As expected by a random sample, our study species follow the known species richness per family, with the most represented being the jumping spiders (Salticidae, 31 species), orb-weavers (Araneidae, 18 species), crab spiders (Thomisidae, 16 species), wolf spiders (Lycosidae, 15 species), ground spiders (Gnaphosidae, 11 species) and sheet weavers or money spiders (Linyphiidae, 11 species). These broadly correspond to the families with more described species to date (World Spider Catalog 2018). All other families in our sample were represented by less than 10 species.

The Neotropics (54 species) and the Palearctic (47 species) were the most represented biogeographical realms (Fig. 1). The Oceanian realm (with only 2 species) and the Antarctic (no species) were scarcely or not represented. This is probably due as much to lack of knowledge as to low species richness in some regions.

**Figure 2.**
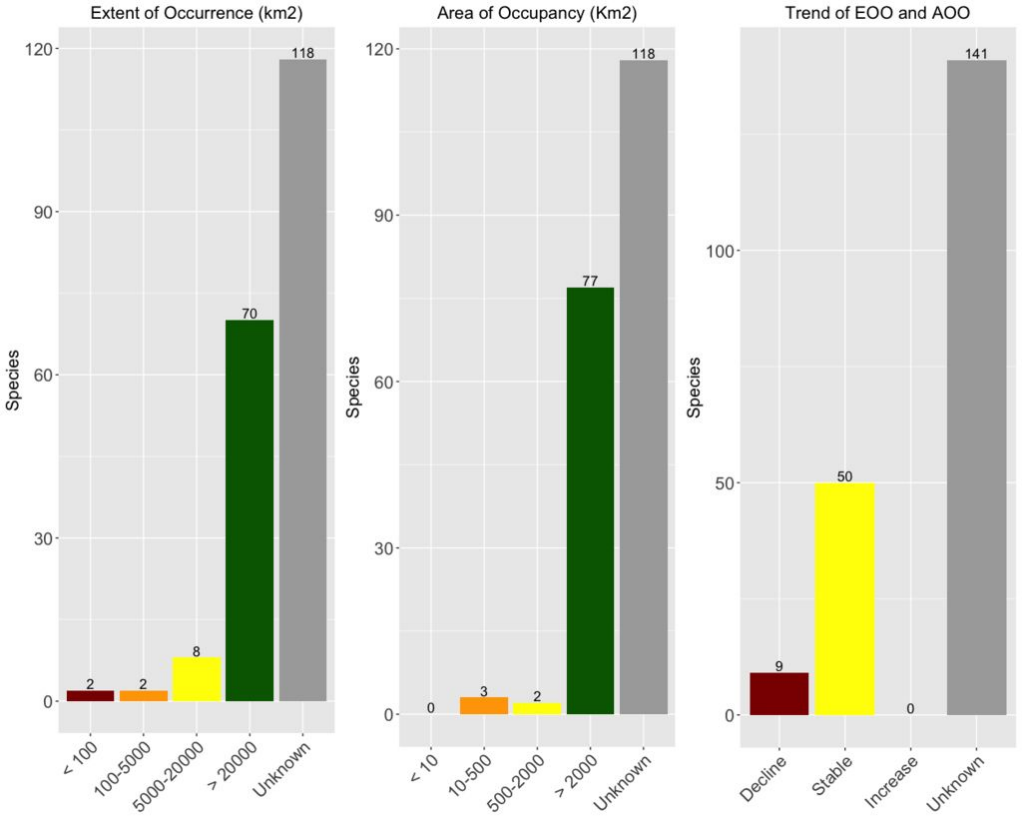
Extent of Occurrence (EOO), Area of Occupancy (AOO) and their trends amongst the 200 assessed species.

*Nephilingis cruentata* (Fabricius, 1775) (Nephilidae), *Tiso aestivus* (L. Koch, 1872) (Linyphiidae) and *Ceratinella brunnea* Emerton, 1882 (Linyphiidae) were the most widespread species, all with an estimated EOO above 30 million km^2^ or AOO above 20 million km^2^. At the other end of the spectrum, *Sesato setosa* Saaristo, 2006 (Theridiidae, from the Seychelles), *Cataxia bolganupensis* (Main, 1985) (Idiopidae, from Australia) and *Zelotes mulanjensis* FitzPatrick, 2007 (Gnaphosidae, from Malawi) all had an EOO and AOO below 300 km^2^. In addition, there is uncertainty if *Galeosoma robertsi* Hewitt, 1916 (Idiopidae, from South Africa) was driven to extinction during the 20th century. Of these, we could find evidence of decline in EOO or AOO for all species but *S. setosa. C. bolganupensis, Z. mulanjensis and G. robertsi* were under severe threat from habitat destruction from wildfires, deforestation and urbanisation, respectively. For 118 of the species in our study, it was not possible to estimate the Extent of Occurrence or Area of Occupancy due to the scarcity of reliable data (Fig. 2). The trends in EOO and AOO were assumed to be stable for 50 out of the 59 species with some data available, although there is no monitoring data for any taxon. Only for nine species we found evidence of decline mainly due to habitat loss or degradation (Fig. 2).

**Figure 3.**
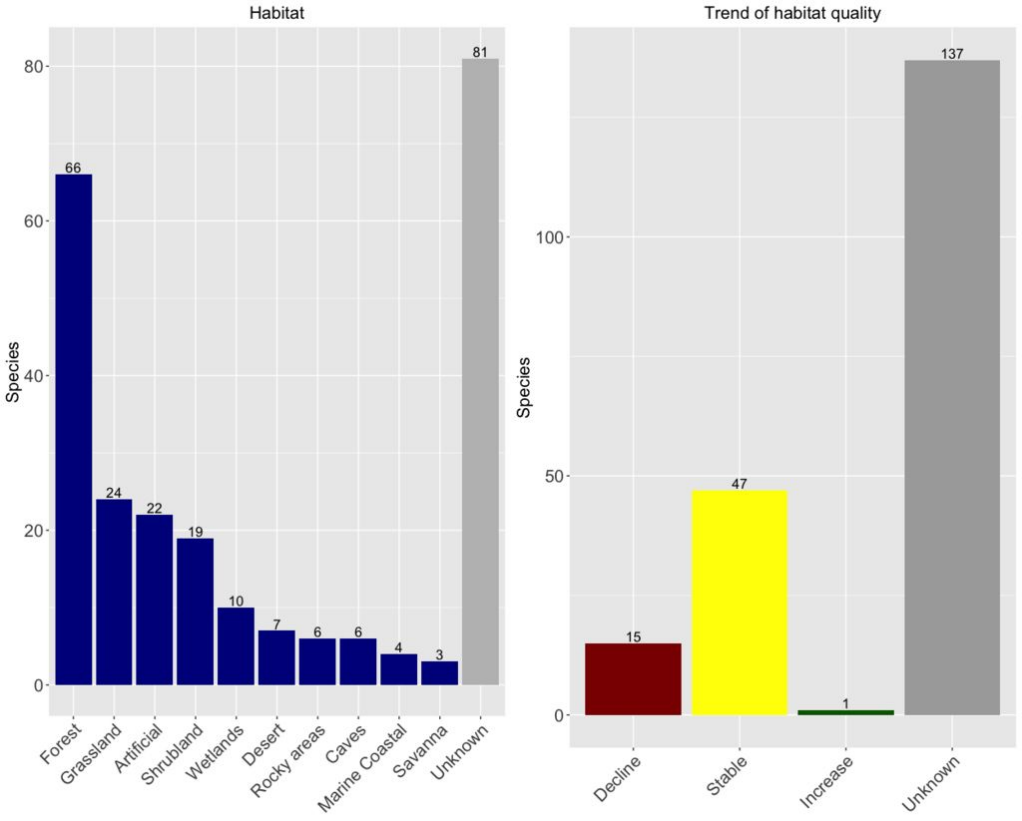
Habitat type and trend in quality of habitat of the 200 assessed species.

Data on habitat was available for 119 of the studied species (Fig. 3). Forest was the most common habitat type (66 species), followed by grasslands (24 species). For 47 species the habitat quality trend was inferred to be stable, only declining for 15 and increasing for 1 species. For the remaining 137 species, the habitat quality trend could not be inferred and was thus classified as unknown (Fig. 3).

**Figure 4.**
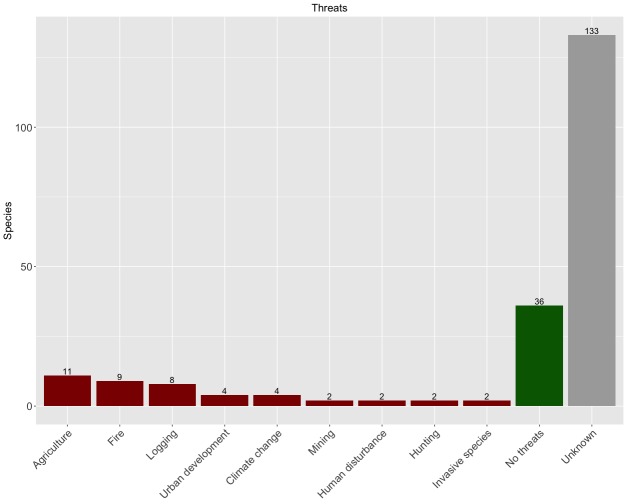
Main threats to the 200 assessed species.

The most common threat types amongst the 31 species for which threat data were available (Fig. 4) were agriculture (11 species), wildfires (9 species) and logging (8 species). In the case of 36 species, there were no known threats to the species, mostly amongst the widespread and well-known taxa. For 133 species, the available information was not sufficient to attribute the existence of any specific kind of threat (Fig. 4).

**Figure 5.**
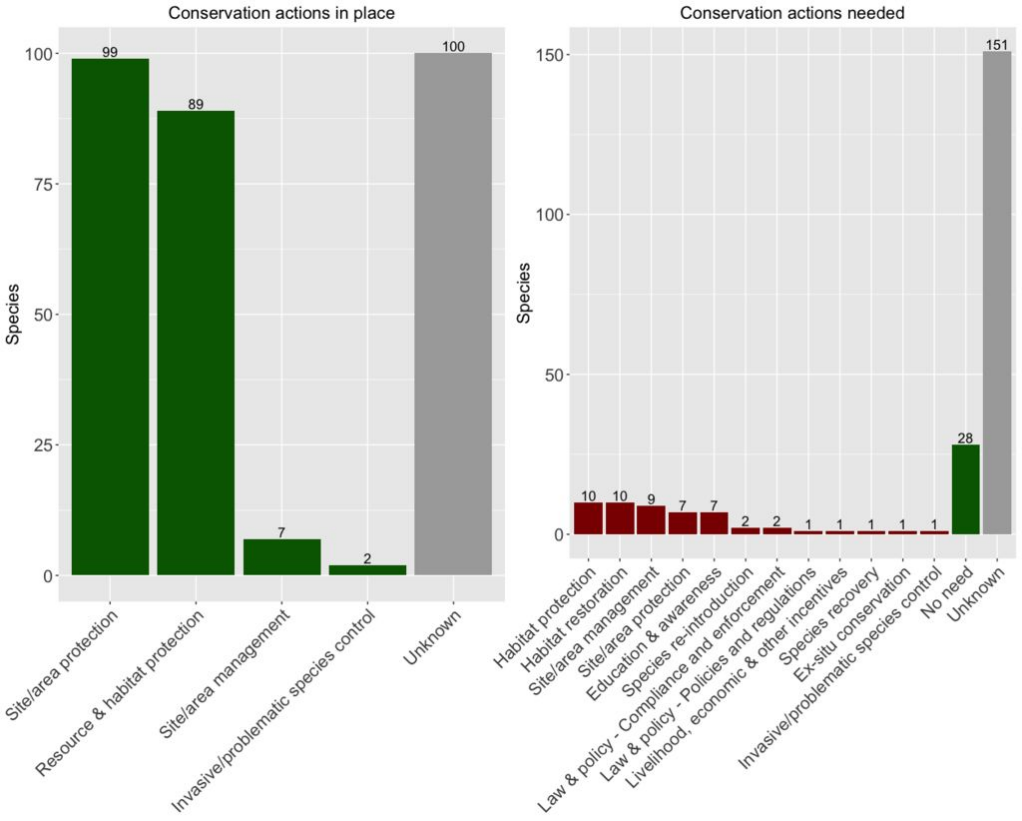
Conservation actions in place and needed for the 200 assessed species.

Many species are known to occur within protected areas or habitats and, therefore, the status of the area itself is the most common conservation action currently in place (Fig. 5), even if, for half the taxa, we had no knowledge of their occurrence in PAs or any other protection measure. However, occurrence within protected areas may not be enough for species survival and a number of other conservation actions were suggested, the most common of which being habitat protection, restoration and management, besides a strong emphasis in education and awareness (Fig. 5).

**Figure 6.**
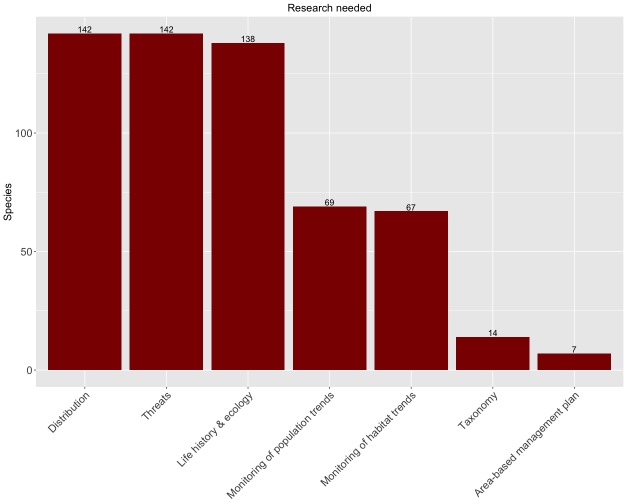
Main research needs for the 200 assessed species.

Finally, we identified a number of research priorities for the future (Fig. 6), the most important of which being to better know the species distribution (the Wallacean shortfall; Lomolino 2004), threats and life history and ecology (the Hutchinsonian shortfall; Mokany and Ferrier 2010). Also monitoring of both population (the Prestonian shortfall; Cardoso et al. 2011a) and habitat trends were deemed critical for many species (Fig. 6).

Results

## Discussion

This exercise provides a first glimpse into the general trends in the conservation status of spiders around the world. Most notably, this research shows that spider species are most commonly affected by habitat destruction, although climate change, invasive species and direct hunting (for the pet trade) are important threats to some taxa as well. Yet, these threats are for known and assessed species, the vast majority of taxa lacking data on their threats (Cardoso et al. 2011a). Even basic data such as distribution and basic life history are unknown for the great majority of spiders. Unfortunately, without such data, it is impossible to suggest conservation measures to all but a few of the best known species. In the meantime, many of them, including some of the 200 that were assessed herein, may already be extinct. In fact, it is probable that many more species became extinct even before they were described or assessed.

We hypothesise that there should be a higher proportion of threatened species amongst the Data Deficient or undescribed species than amongst those with reasonable information available. This hypothesis is based on two reasons. First, the scarcity of information on many species is often partly due to their rarity. These rare species are harder to collect and hence have a higher probability of remaining undescribed or unrecorded for longer periods or lacking data for their assessment. Exceptions might occur in relatively well-known areas, where rare species are specifically targeted and often better known than common ones. Second, widespread species are often the only ones for which an assessment may be conducted as they are assumed to have stable populations, creating a bias in the dataset towards a large proportion of non-threatened species. This means that a random sample of species, such as the approach followed herein and recognised by the SRLI (Baillie et al. 2008), might not reflect reality. SRLI values reached are probably higher than the real trend and we incur the risk of painting a more optimistic picture than reality. The strategy currently used by the IUCN is therefore inadequate for taxa with scarce information, which represent the vast majority of species within diverse, poorly known groups such as spiders. Hence, we are currently working on a non-random approach for the selection of species for the SRLI (Henriques et al. in prep.).

The Convention on Biological Diversity (CBD) in Aichi, Japan, declared 2010–2020 as the decade of biodiversity. Twenty biodiversity targets were set to be met by the year 2020 (Tittensor et al. 2014). Amongst these, target 12 says “**by 2020 the extinction of known threatened species has been prevented and their conservation status, particularly of those most in decline, has been improved and sustained**”. Yet, if we do not have extinction risk information for the vast majority of species, even if only for the fraction described to date, it is impossible to know how close we are to such a target. This research revealed important information about global trends on the threat status of spider species. However, it also revealed how much we still need to discover to even begin to be able to provide any definitive answers on the threat status of spiders at a global level.

Discussion

## Supplementary Material

Supplementary material 1 Distribution of *Dictis denticulata* Dankittipakul & Singtripop, 2010 Data type: Distribution
File: oo_173342.kml Cardoso, P.

Supplementary material 2 Distribution of *Scytodes cogu* Brescovit & Rheims, 2001 Data type: Distirbution
File: oo_173345.kml Cardoso, P.

Supplementary material 3 Distribution of *Selenops candidus* Muma, 1953 Data type: Distribution
File: oo_173346.kml Cardoso, P.

Supplementary material 4 Distribution of *Selenops shevaroyensis* Gravely, 1931 Data type: Distribution
File: oo_173347.kml Cardoso, P.

Supplementary material 5 Distribution of *Loxosceles devia* Gertsch & Mulaik, 1940 Data type: Distribution
File: oo_173348.kml Cardoso, P.

Supplementary material 6 Distribution of *Heteropoda jiangxiensis* Li, 1991 Data type: Distribution
File: oo_173349.kml Cardoso, P.

Supplementary material 7 Distirbution of *Isopeda echuca* Hirts, 1992 Data type: Distribution
File: oo_173350.kml Cardoso, P.

Supplementary material 8 Distribution of *Pseudopoda parvipunctata* Jäger, 2001 Data type: Distribution
File: oo_173340.kml Cardoso, P.

Supplementary material 9 Distribution of *Sinopoda sitkao* Jäger, 2012 Data type: Distribution
File: oo_173351.kml Cardoso, P.

Supplementary material 10 Distribution of *Tetrablemma brevidens* Tong & Li, 2008 Data type: Distribution
File: oo_173352.kml Cardoso, P.

Supplementary material 11 Distribution of *Chrysometa lepida* (Keyserling, 1881) Data type: Distribution
File: oo_173364.kml Cardoso, P.

Supplementary material 12 Distribution of *Cyrtognatha pachygnathoides* (O. Pickard-Cambridge, 1894) Data type: Distribution
File: oo_203766.kml Cardoso, P.

Supplementary material 13 Distribution of *Brachionopus tristis* Purcell, 1903 Data type: Distribution
File: oo_173372.kml Cardoso, P.

Supplementary material 14 Distribution of *Cardiopelma mascatum* Vol, 1999 Data type: Distribution
File: oo_173373.kml Cardoso, P.

Supplementary material 15 Distribution of *Cyriopagopus vonwirthi* Schmidt, 2005 Data type: Distribution
File: oo_173374.kml Cardoso, P.

Supplementary material 16 Distribution of *Eupalaestrus larae* Ferretti & Barneche, 2012 Data type: Distribution
File: oo_222731.kml Cardoso, P.

Supplementary material 17 Distribution of *Phormictopus platus* Chamberlin, 1917 Data type: Distribution
File: oo_222730.kml Cardoso, P.

Supplementary material 18 Distribution of *Plesiopelma myodes* Pocock, 1901 Data type: Distribution
File: oo_173378.kml Cardoso, P.

Supplementary material 19 Distribution of *Poecilotheria subfusca* Pocock, 1895 Data type: Distribution
File: oo_173379.kml Cardoso, P.

Supplementary material 20 Distribution of *Dipoeana appalachia* Levi, 1953 Data type: Distribution
File: oo_173383.kml Cardoso, P.

Supplementary material 21 Distribution of *Lasaeola convexa* (Blackwall, 1870) Data type: Distribution
File: oo_173388.kml Cardoso, P.

Supplementary material 22 Distribution of *Sesato setosa* Saaristo, 2006 Data type: Distribution
File: oo_173390.kml Cardoso, P.

Supplementary material 23 Distribution of *Steatoda xerophila* Levy & Amitai, 1982 Data type: Distribution
File: oo_173391.kml Cardoso, P.

Supplementary material 24 Distribution of *Theridion miserum* Thorell, 1898 Data type: Distribution
File: oo_207834.kml Cardoso, P.

Supplementary material 25 Distribution of *Theridion xiangfengense* Zhu & Song, 1992 Data type: Distribution
File: oo_173394.kml Cardoso, P.

Supplementary material 26 Distribution of *Thymoites pictipes* (Banks, 1904) Data type: Distribution
File: oo_173395.kml Cardoso, P.

Supplementary material 27 Distribution of *Thymoites verus* (Levi, 1959) Data type: Distribution
File: oo_173397.kml Cardoso, P.

Supplementary material 28 Distribution of *Ogulnius infumatus* Simon, 1897 Data type: Distribution
File: oo_173398.kml Cardoso, P.

Supplementary material 29 Distribution of *Theridiosoma concolor* Keyserling, 1884 Data type: Distribution
File: oo_173399.kml Cardoso, P.

Supplementary material 30 Distribution of *Bomis bengalensis* Tikader, 1962 Data type: Distribution
File: oo_173400.kml Cardoso, P.

Supplementary material 31 Distribution of *Epicadus trituberculatus* Taczanowski, 1872 Data type: Distribution
File: oo_173401.kml Cardoso, P.

Supplementary material 32 Distribution of *Misumena picta* Franganillo, 1926 Data type: Distribution
File: oo_173402.kml Cardoso, P.

Supplementary material 33 Distribution of *Misumenoides gwarighatensis* Gajbe, 2004 Data type: Distribution
File: oo_173403.kml Cardoso, P.

Supplementary material 34 Distribution of *Misumenops guianensis* (Taczanowski, 1872) Data type: Distribution
File: oo_173404.kml Cardoso, P.

Supplementary material 35 Distribution of *Misumenops ignobilis* (Badcock, 1932) Data type: Distribution
File: oo_173405.kml Cardoso, P.

Supplementary material 36 Distribution of *Oxytate greenae* (Tikader, 1980) Data type: Distibution
File: oo_173406.kml Cardoso, P.

Supplementary material 37 Distribution of *Ozyptila conspurcata* Thorell, 1877 Data type: Distribution
File: oo_173407.kml Cardoso, P.

Supplementary material 38 Distribution of *Ozyptila hardyi* Gertsch, 1953 Data type: Distribution
File: oo_173408.kml Cardoso, P.

Supplementary material 39 Distribution of *Stephanopis yulensis* Thorell, 1881 Data type: Distribution
File: oo_173409.kml Cardoso, P.

Supplementary material 40 Distribution of *Synema adjunctum* O. Pickard-Cambridge, 1891 Data type: Distribution
File: oo_203769.kml Cardoso, P.

Supplementary material 41 Distribution of *Synema hildebrandti* Dahl, 1907 Data type: Distribution
File: oo_173411.kml Cardoso, P.

Supplementary material 42 Distribution of *Thomisus litoris* Strand, 1913 Data type: Distribution
File: oo_173412.kml Cardoso, P.

Supplementary material 43 Distribution of *Tmarus peruvianus* Berland, 1913 Data type: Distribution
File: oo_173413.kml Cardoso, P.

Supplementary material 44 Distribution of *Xysticus kalandadzei* Mcheidze & Utochkin, 1971 Data type: Distribution
File: oo_222729.kml Cardoso, P.

Supplementary material 45 Distribution of *Xysticus tristrami* (O. Pickard-Cambridge, 1891) Data type: Distribution
File: oo_203771.kml Cardoso, P.

Supplementary material 46 Distribution of *Longrita rastellata* Platnick, 2002 Data type: Distribution
File: oo_173416.kml Cardoso, P.

Supplementary material 47 Distribution of *Cavasteron guttulatum* Baehr & Jocqué, 2000 Data type: Distribution
File: oo_173417.kml Cardoso, P.

Supplementary material 48 Distribution of *Storena gujaratensis* Tikader & Patel, 1975 Data type: Distribution
File: oo_173418.kml Cardoso, P.

Supplementary material 49 Distribution of *Zodarion sytchevskajae* (Nenilin & Fet, 1985) Data type: Distribution
File: oo_173419.kml Cardoso, P.

Supplementary material 50 Distribution of *Huntia deepensis* Gray & Thompson, 2001 Data type: Distribution
File: oo_173420.kml Cardoso, P.
